# Access to Spiropyrazolone-butenolides through NHC-Catalyzed [3 + 2]-Asymmetric Annulation of 3-Bromoenals and 1*H*-Pyrazol-4,5-diones

**DOI:** 10.1021/acs.joc.3c00188

**Published:** 2023-05-11

**Authors:** Marta Gil-Ordóñez, Alicia Maestro, José M. Andrés

**Affiliations:** GIR-SintACat-Instituto Universitario CINQUIMA y Departamento de Química Orgánica, Facultad de Ciencias, Universidad de Valladolid, Paseo Belén 7, 47011 Valladolid, Spain

## Abstract

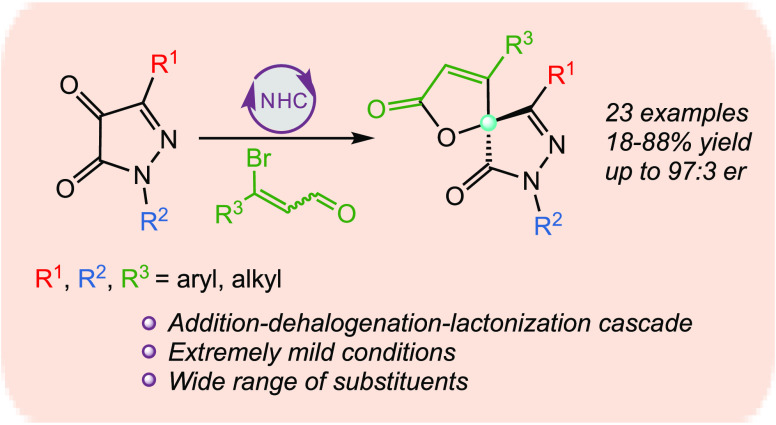

The stereoselective synthesis of spirocyclic pyrazolin-5-ones by N-heterocyclic carbene (NHC) organocatalysis has been less studied so far. For this reason and considering the interest of this class of compounds, here, we present the NHC-catalyzed [3 + 2]-asymmetric annulation of β-bromoenals and 1*H*-pyrazol-4,5-diones that achieves to produce chiral spiropyrazolone-butenolides. The synthesis is general for aryl and heteroaryl β-bromo-α,β-unsaturated aldehydes and 1,3-disubstituted pyrazolones. The spirobutenolides have been obtained in good yields (up to 88%) and enantioselectivities (up to 97:3 er). This constitutes the first described example using pyrazoldiones as the starting materials for this class of spiro compounds.

## Introduction

**Figure 1:**
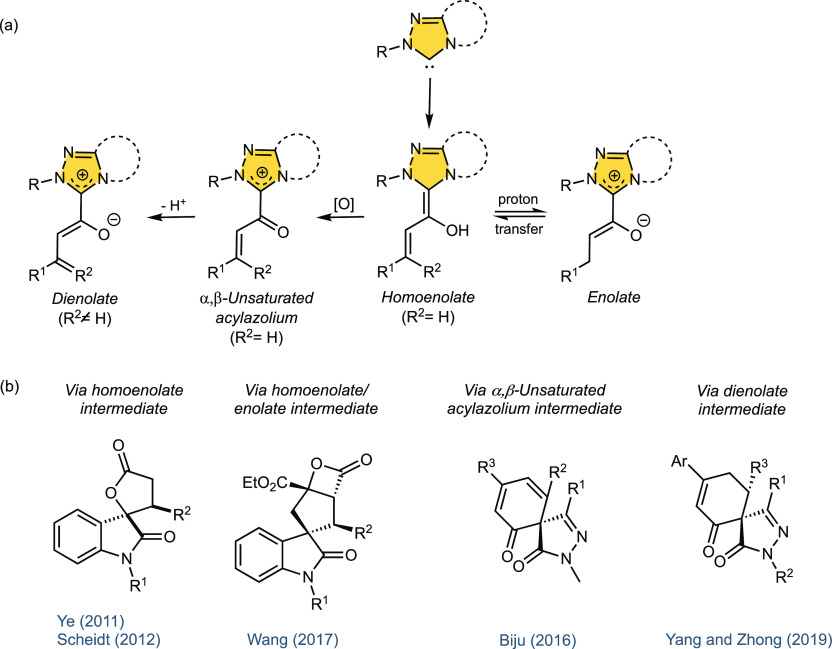
(a) Typical reaction intermediates in NHC catalysis. (b) Some selected examples of chiral spirooxindoles and spiropyrazolones accessible via NHC-mediated pathways.

Since Sheehan and Hunneman^1^ carried out the first enantioselective benzoin reaction more than fifty years ago, asymmetric organocatalysis enabled by N-heterocyclic carbenes (NHCs) has continued to evolve, especially during the last two decades. The progress in the knowledge of the intermediates formed when aldehydes and other carbonyls are activated with NHCs allows us to look at more specific fields such as catalysis via homoenolate,^2^ via α,β-unsaturated acylazolium,^3^ via azolium enolate,^4^ or via azolium dienolate^5^ (Figure 1a). In general, these NHC-catalyzed transformations are operationally simple reactions that proceed at room temperature without the generation of reaction byproducts. If the precursor NHC/base combination is well selected, it is possible to prepare structurally complex molecules from easy the starting materials. In addition, high diastereo- and enantioselectivity levels are possible using chiral NHCs.

The synthesis of new chiral spiroheterocycles remains one of the most important goals for synthetic chemists because they are privileged scaffolds widely occurring in many natural products and drugs. Diverse examples can be found in the literature describing its preparation by NHC catalysis, mainly spirooxindole derivatives (Figure 1b, left). For example, in a pioneering work, Ye et al.^6^ reported the stereoselective synthesis of spirooxindole lactones by NHC-catalyzed homoenolate annulation of enals with isatins. Similar structures were obtained by the Scheidt group,^7^ who used a cooperative catalysis with lithium chloride as Lewis acid. Using in situ generated enolate species, an asymmetric Michael-intramolecular aldol-lactonization cascade reaction to propiolactone-fused spirocyclopentane-oxindoles^8^ was developed by Wang and coworkers. Nonetheless, other spirocyclic heterocycles, such as spiropyrazolones, have been much less studied despite their biologically and pharmacologically relevant properties.^9^ The challenging stereoselective generation of their C-4 quaternary stereocenter is highly desirable because it provides a three-dimensional structure crucial for the behavior of potential drug candidates.^10^ In particular, drawbacks such as the low spatial occupation or the limitation of their interactions with the three-dimensional structure of the target molecules that present the more traditional achiral planar (hetero)aromatic compounds can be minimized. Very few examples describe the NHC-catalyzed asymmetric synthesis of spirocyclic pyrazolones^11^ (Figure 1b, right). The groups of Biju and Yang–Zhong have reported the preparation of pyrazolone spirocyclohexanones by addition of NHC-generated α,β-unsaturated acylazolium^11c^ or vinyl enolate^11a^ intermediates from enals and γ-chloro enals, respectively, to α-arylidene pyrazolinones. Very recently, we have described the first asymmetric synthesis of spirocyclic pyrazolone γ-butyrolactones by an NHC-catalyzed [3 + 2] annulation reaction.^12^

On the other hand, the γ-butenolide moiety is a motif present in a wide range of natural products and biologically active molecules. In the last few years, several reviews have documented the most recent enantioselective synthetic approaches for the construction of these frameworks,^13^ but the development of this field is still in its infancy. In fact, to our knowledge, no reports on the synthesis of chiral spiropyrazolone-butenolides have been disclosed so far.

**Scheme 1:**
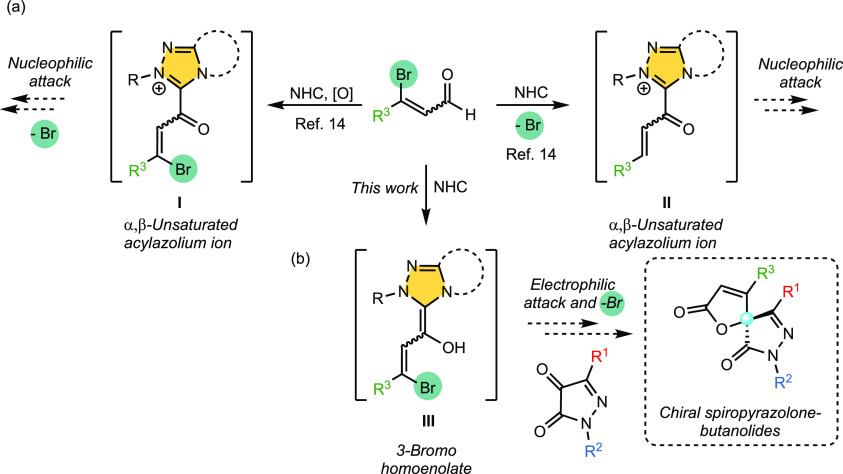
Use of 3-Haloenals under NHC Catalysis

Ma et al. disclosed that the use of 3-haloenals under NHC catalysis allows the selective generation of two types of α,β-unsaturated acylazolium intermediates **I** and **II**, depending on the presence or absence of an external oxidant (Scheme 1a).^14^ However, the use of NHC-bound homoenolates with a bromine atom in the β-position **III** has enabled an addition–dehalogenation–lactonization cascade process.^15^ Since we are interested in the preparation of enantiopure 4,4-disubstituted pyrazol-5-one derivatives, this possibility prompted us to carry out the reaction with pyrazolin-4,5-diones that have not yet been used as the starting materials for the synthesis of spiropyrazolones (Scheme 1b). As a result of this idea, we now present the enantioselective synthesis of novel chiral spiropyrazolone-butenolides via 3-halogen-substituted homoenolates promoted by NHC catalysis.

## Results and Discussion

We initiated our investigation by reacting pyrazolin-4,5-dione **1a** with 3-bromo cinnamaldehyde **2a** (1.5 equiv) in the presence of chiral triazolium precatalysts **A–C** (10 mol %), DBU (1.5 equiv) as base in THF. The reactions were maintained at room temperature for 16 h (Table 1). A screening of NHC precursors (entries 1, 2, and 4–6) indicated that pyroglutamic derivative **A2** and **C2**, a modified Bode precatalyst, provided the highest enantiomeric ratio values for pyrazolone-butenolide **3a** (entries 2 and 6), so we decided to test them under different reaction conditions. Performing the reaction at 50 °C in the presence of **A**2 (entry 3) resulted in less than 5% conversion and diminished er. Switching THF to chloroform, 1,4-dioxane or a THF/*t*BuOH (10:1) mixture was ineffective (entries 7–9), and in hexane, diethyl ether, or a THF/MeCN (1:1), the reaction did not proceed (entries 10–12). Other bases to generate the carbene catalyst did not improve the results. The yield rose to 71% when cesium carbonate was used instead of DBU, but the enantioselectivity decreased (entry 13). The presence of lithium chloride as additive was not beneficial (entry 14), and other bases such as TBD or a mixture of DBU and cesium carbonate provided product **3a** with similar levels of enantioselectivity but inferior yield (entries 15 and 16). The same happened when using *t*BuOK or DMAP (entries 17 and 18). Changes in **1a**/**2a** molar ratio provided very poor yields (entries 19 and 20). Treatment of the triazolium salt **C2** with DBU in chloroform (entry 21) improved slightly the enantioselectivity compared to the reaction in THF. No significant improvement was achieved by performing the reaction at 0 °C (entry 22). Other solvents provided the pyrazolone-butenolide **3a** in better yields, but the enantioselectivity decreased (entries 23–25). Again, a good yield for spiropyrazolone was obtained when the catalyst was formed from **C2** using cesium carbonate (entry 26), but the enantioselectivity was worse. Finally, TBD and DMAP (entries 27 and 28) gave lower yields and enantiomeric ratios. Overall, the best balance between yield and enantiomeric ratio was achieved using a combination of **C2**/DBU/chloroform (entry 21).

**Table 1:**
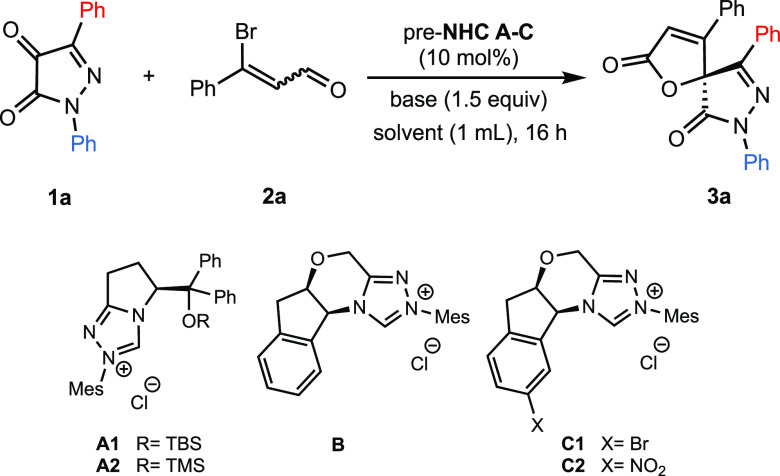
Optimization of Reaction Conditions^a^

entry	pre-NHC	base	solvent	yield (%)^b^	er^c^
1	**A1**	DBU	THF	n.r.	n.r.
2	**A2**	DBU	THF	27	96:4
3^d^	**A2**	DBU	THF	<5	88:12
4	**B**	DBU	THF	56	81:19
5	**C1**	DBU	THF	<5	32:68
6	**C2**	DBU	THF	57	92:8
7	**A2**	DBU	CHCl_3_	14	88:12
8	**A2**	DBU	dioxane	9	95:5
9	**A2**	DBU	THF/*t*BuOH (10:1)	33	88:12
10	**A2**	DBU	Et_2_O	n.r.	n.r.
11	**A2**	DBU	hexane	n.r.	n.r.
12	**A2**	DBU	THF/MeCN (1:1)	n.r.	n.r.
13	**A2**	Cs_2_CO_3_	THF	71	88:12
14^e^	**A2**	Cs_2_CO_3_	THF	<5	89:11
15	**A2**	TBD	THF	20	95:5
16	**A2**	DBU + Cs_2_CO_3_	THF	20	96:4
17	**A2**	*t*BuOK	THF	16	91:9
18	**A2**	DMAP	THF	47	86:14
19^f^	**A2**	DBU	THF	14	98:2
20^g^	**A2**	DBU	THF	27	96:4
21	**C2**	DBU	CHCl_3_	54	94:6
22^h^	**C2**	DBU	CHCl_3_	52	94:6
23	**C2**	DBU	DCE	74	83:17
24	**C2**	DBU	dioxane	74	79:21
25	**C2**	DBU	MeTHF	55	88:12
26	**C2**	Cs_2_CO_3_	CHCl_3_	70	90:10
27	**C2**	TBD	CHCl_3_	29	86:14
28	**C2**	DMAP	CHCl_3_	14	50:50

a Reaction conditions: **1a** (0.06 mmol), **2a** (0.09 mmol), pre-NHC (10 mol %), base (1.5 equiv), solvent (1 mL), at rt for 16 h.

b Yield of **3a** after column chromatography.

c Er values determined via chiral high-performance liquid chromatography (HPLC) analysis.

d Reaction temperature 50 °C.

e LiCl (2 equiv) as additive.

f Molar ratio **1a**/**2a** 1:1.

g Molar ratio **1a**/**2a** 1.5:1.

h Reaction temperature 0 °C.

**Scheme 2:**
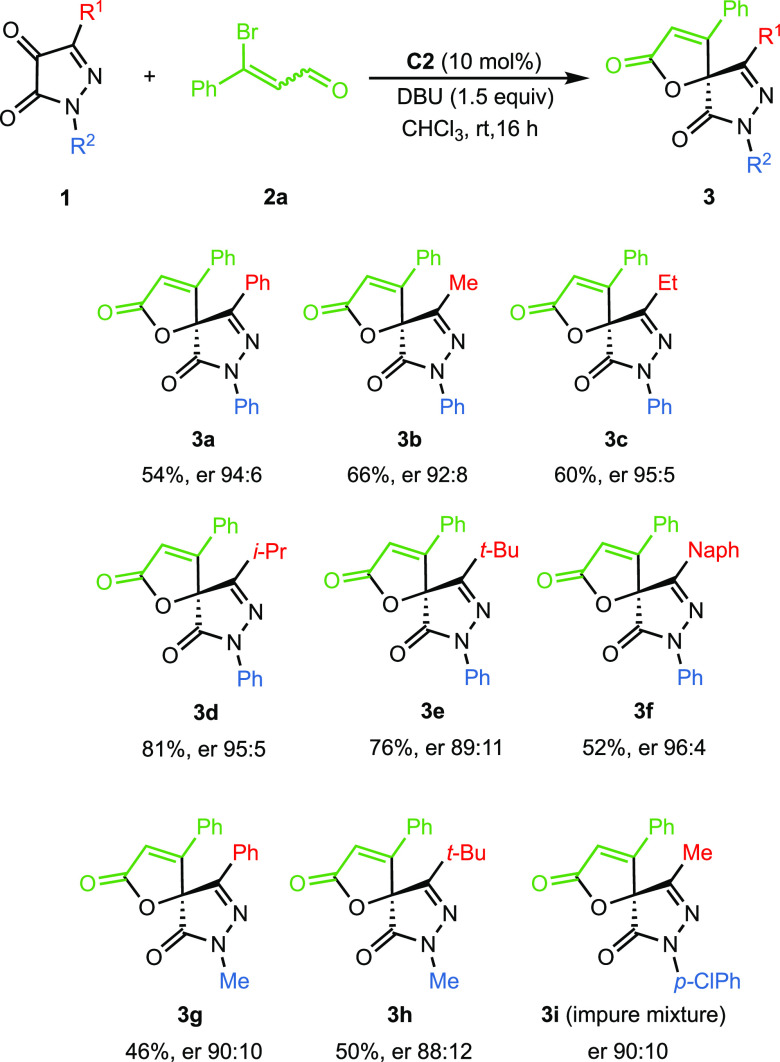
Scope of Reaction with Respect to Pyrazolin-4,5-dione^,b,c^ Reaction conditions: **1a−i** (0.06 mmol), **2a** (0.09 mmol), **C2** (10 mol %), DBU (1.5 equiv), chloroform (1 mL), at rt for 16 h. ^b^Yield of **3** after column chromatography. ^c^Er values determined via chiral HPLC analysis.

Once the optimal reaction conditions were established, we then explored the influence of the substituents of pyrazolin-4,5-dione. To do this, 3-bromo cinnamaldehyde **2a** was reacted with pyrazolin-4,5-diones **1** with different substituents at C-3 and N-1 positions (Scheme 2). Regardless of whether an alkyl or an aryl group was present at C-3, the enantioselectivity was high. In all cases, the spirocycles **3a–f** were obtained in moderate to good yields. NMR analysis of the reaction crude indicated a total conversion of the starting pyrazole-dione, so it is possible that the decrease in the yield is due to the formation of nonidentifiable byproducts. On the other hand, the influence of the N-substituent has also been considered and a slight drop of performance was detected when an *N*-methyl group was present in the butenolide (**3g** and **3h**). Similar result was observed for compound **3i**, with a *p*-chlorophenyl group at the N-1 position, that showed a good enantiomeric ratio, 90:10.^16^

**Scheme 3:**
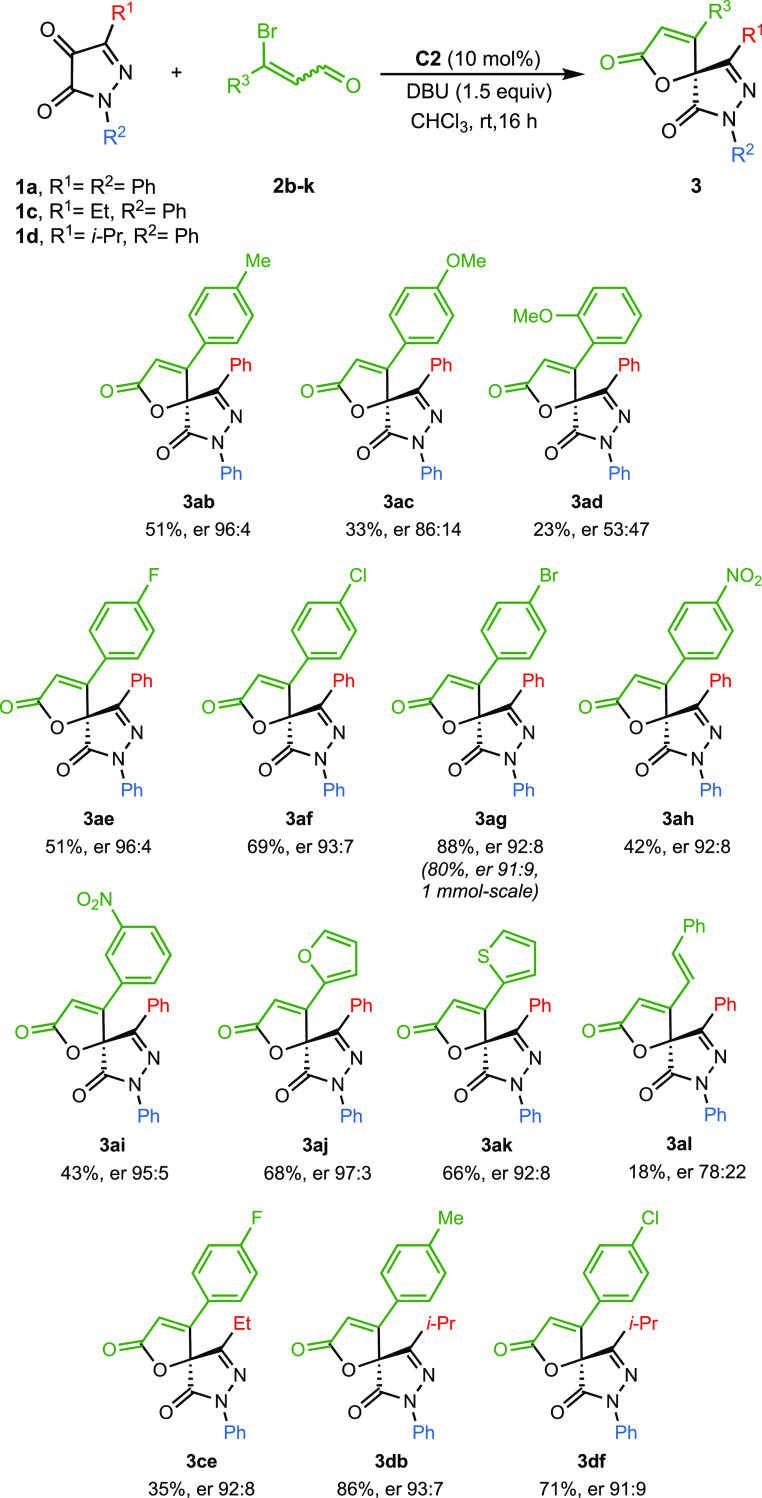
Substrate Scope Involving β-bromoenals^,b,c^ Reaction conditions: **1a**,**c**,**d** (0.06 mmol), **2b-l** (0.09 mmol), **C2** (10 mol %), DBU (1.5 equiv), chloroform (1 mL), at rt for 16 h. ^b^ Yield of **3** after column chromatography. ^c^ Er values determined via chiral HPLC analysis.

Next, the influence of the β-bromoenal on the annulation reaction was evaluated. For this purpose, aryl and heteroaryl β-bromo-α,β-unsaturated aldehydes **2b–l** were used (Scheme 3). The process worked well when the pyrazolin-4,5-dione **1a** was reacted with *p*-substituted cinnamyl aldehydes, and the corresponding spiropyrazolones were isolated (**3ab–3ac** and **3ae**–**3ah**). The enantioselectivities were excellent when electron-withdrawing or donating groups were present on the enal, except for compound **3ac** that gave a slightly lower enantiomeric ratio. However, a quasi-racemic mixture and poor yield were obtained when the enal had a methoxy group in the *ortho*-position of the phenyl ring (**3ad**). On the other hand, the *meta*-substitution of the aromatic ring did not lead to a significant change in either the yield or the enantioselectivity (**3ah** and **3ai**). The reaction of **1a** with 3-bromo-3-furanyl or 3-thienyl acrylaldehyde resulted in the corresponding spiropyrazolones **3aj** and **3ak** in good yield and er. Again, and regardless of the enal substitution, the enantiomeric ratios remained high when the pyrazolin-4,5-dione had an ethyl or isopropyl group at C-3 (**3ce**, **3db,** and **3df**). To further demonstrate the feasibility of our protocol, a scale-up reaction was conducted on a 1-mmol scale for the preparation of spirobutenolide **3ag**, and good yield (367 mg, 80% yield) and similar enantioselectivity (er 91:9) were achieved in the presence of 10 mol % catalyst **C2** (Scheme 3). Finally, the reaction using β-bromo benzylidene crotonaldehyde afforded the spirocyclic butenolide **3al** in low yield and moderate enantioselectivity (18%, er 78:22).

**Scheme 4:**
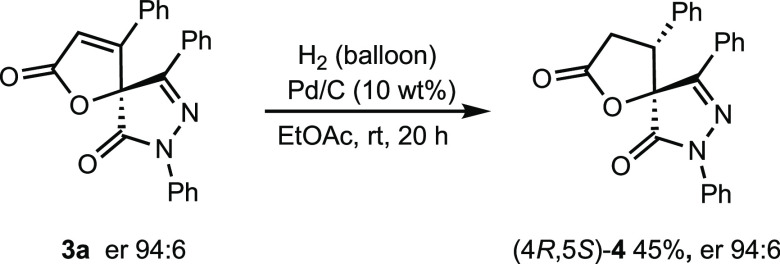
Selective Hydrogenation of SpiroButenolide **3a**

The stereochemistry of the major enantiomer of **3a** was established by chemical correlation with spirocyclic pyrazolone γ-butyrolactone **4**,^12^ obtained by catalytic hydrogenation of **3a** (Scheme 4. See the Supporting Information for details and retention times for (4*R*,5*S*)-**4** and racemic-**4**). The absolute configuration of the other products is expected to be the same by analogy.

**Scheme 5:**
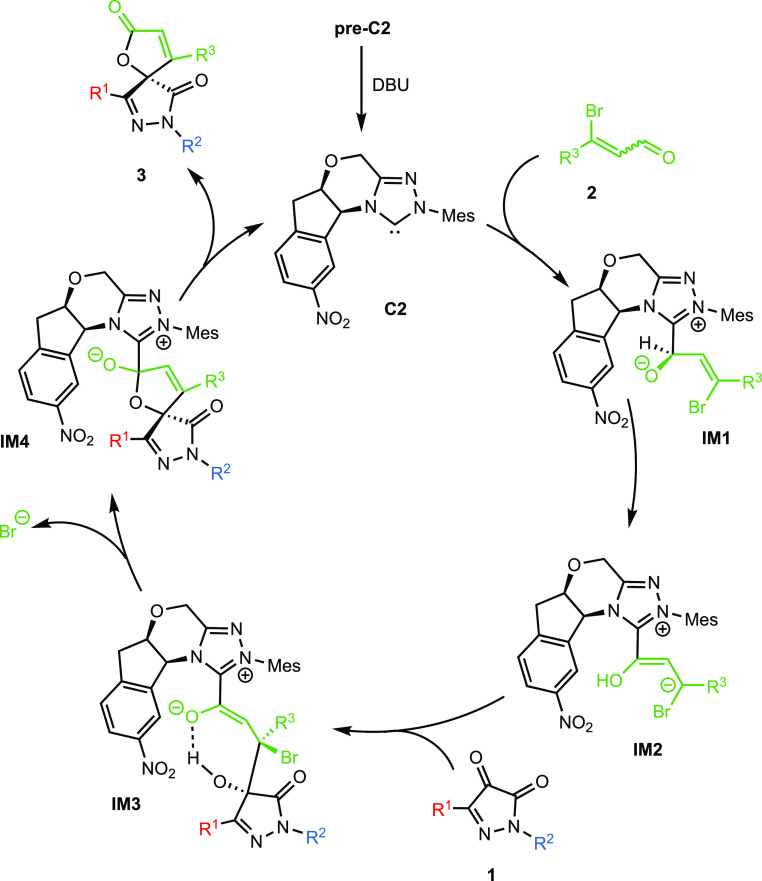
Plausible Catalytic Cycle

A plausible catalytic cycle for the NHC-catalyzed [3 + 2]-annulation reaction is depicted in Scheme 5. In the first stage, the NHC catalyst reacts with the aldehyde moiety of β-bromoenal **2**, giving rise to the intermediate **IM1** which evolves to the Breslow homoenolate **IM2** after base-assisted 1,2-hydrogen migration.^12,17^ In our previous electronic structure calculations, we proved that the Brønsted base used to generate the carbene catalyst assists the [1,2]-proton transfer for the generation of homoenolate. However, it is also worth mentioning that previous computational studies have found that it is the conjugated acid of the base that leads to the lowest energy barrier.^18^ Subsequently, the attack of the *Re* face of pyrazole-dione **1** on the *Re* face of homoenolate would be possible, giving rise to the formation of the intermediate **IM3**. Then, the cyclization and release of the bromide anion originate the butenolide unit in **IM4**. This C–O bond-forming event was found to be the stereoselectivity-determining step by the electronic structure calculations for a related reaction, the asymmetric annulation between pyrazolin-4,5-diones and enals.^12^ In addition, the free energy barrier was lower for the stereoisomer with the (*S*) spiro center, which is the configuration obtained experimentally for **3a** (Scheme 4). Finally, the catalytic cycle is completed with the formation of the butenolide product **3** and the regeneration of the NHC catalyst.

**Scheme 6:**
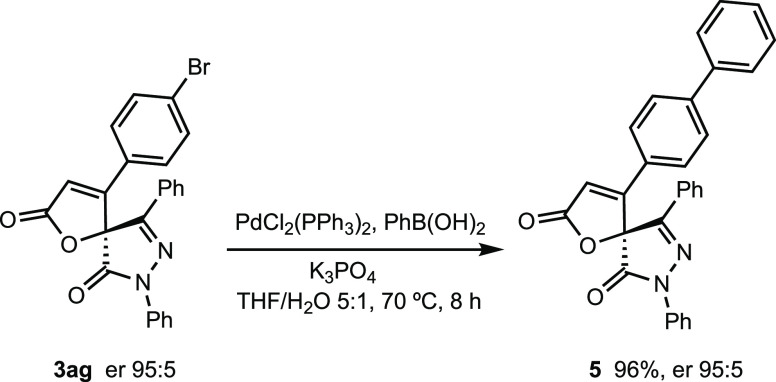
Transformation of Spiropyrazolone-butenolide **3ag**

The synthetic utility of our method was demonstrated by the treatment of *p*-bromophenyl-substituted butenolide **3ag** with phenylboronic acid under Suzuki conditions, and the cross-coupling product **5** was obtained with no erosion of the enantiomeric purity and excellent yield (Scheme 6).

**Scheme 7:**
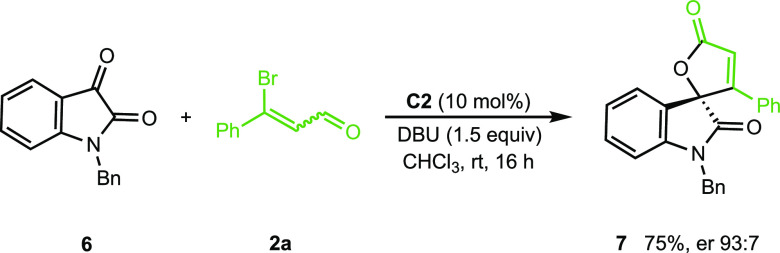
Reaction of *N*-Benzyl Isatin with 3-Bromo Cinnamaldehyde

Finally, although the aim of this work was the preparation of spiropyrazolone-butenolides, we extended our study to other ketones. The reaction of *N*-benzyl isatin **6** with **2a** in the previously established conditions afforded the spirooxindole butenolide **7**(15b) in 75% yield and good enantioselectivity (Scheme 7), thus providing evidence of the versatility of our synthetic methodology.

## Conclusions

In summary, we present an unprecedented strategy for the synthesis of novel chiral spiropyrazolone-butenolides. The key step consists of the use of 3-bromo homoenolates formed from β-bromoenals and a modified Bode catalyst with a nitro substituent on the indene ring. These intermediates trigger an addition–dehalogenation–lactonization cascade by reacting with the pyrazolin-4,5-diones. The process works under extremely mild conditions with a simple procedure and also tolerates a wide range of substituents on both substrates. The S-configuration of the quaternary center created at the C-4 position of the pyrazolone is consistent with our computational studies of the mechanism previously performed in the preparation of γ-butyrolactone derivatives.

## Experimental Section

### General Methods

^1^H NMR (500 MHz), ^13^C NMR (126 MHz), and ^19^F NMR (376 MHz) spectra were recorded in CDCl_3_ as solvent. Chemical shifts for protons are reported in ppm from TMS with the residual CHCl_3_ resonance as an internal reference. Chemical shifts for carbons are reported in ppm from TMS and are referenced to the carbon resonance of the solvent. Data are reported as follows: chemical shift, multiplicity (s = singlet, d = doublet, t = triplet, q = quartet, m = multiplet, and br = broad), coupling constants in hertz, and integration. Specific rotations were measured on a PerkinElmer 341 digital polarimeter using a 1 mL cell with a 1 dm path length, at 589 nm, and concentration is given in g per 100 mL. Infrared spectra were recorded on a PerkinElmer Spectrum One FT-IR spectrometer and are reported in the frequency of absorption (only the structurally most important peaks are given). Flash chromatography was carried out using a silica gel (230–240 mesh). Thin-layer chromatography (TLC) analysis was performed on glass-backed plates coated with a silica gel 60 and F254 indicator and visualized by either UV irradiation or by staining with phosphomolybdic acid solution. Chiral HPLC analysis was performed on a JASCO HPLC system (JASCO PU-2089 and UV-2075 UV/vis detector) with a quaternary pump and on Hewett-Packard 1090 Series II instrument equipped with a quaternary pump, using Phenomenex Lux-amylose-1, Lux-i-amylose-1, and Lux-i-cellulose-5; and Chiralpak OD, IA, and AD-H analytical columns (250 × 4.6 mm). Detection was monitored at 210, 220, 230, and 254 nm. ESI mass spectra were obtained on an Agilent 5973 inert GC/MS system.

Commercially available organic and inorganic compounds were used without further purification. Solvents were dried and stored over microwave-activated 4 Å molecular sieves. Pyrazolin-4,5-diones **1a–i**,^12,19^*N*-benzyl isatin **6**,^20^ β-bromoenals **2a–l,**(21) and triazolium salts used as precatalysts **A–C**(22) were prepared according to the literature procedures. The racemic samples of spiropyrazolone-butenolides were prepared using an equimolar mixture of both enantiomers of precatalyst **B**.

### General Procedure for Spiropyrazolone-butenolides

In a 5-mL heat gun-dried flask equipped with a magnetic stirring bar, the precatalyst **C2** (6 μmol, 0.1 equiv) and the pyrazolin-4,5-dione **1a–i** (0.06 mmol) were weighed. Then, β-bromoenal **2a–l** (0.09 mmol, 1.5 equiv) was added under a N_2_ atmosphere. Dry chloroform (1 mL) was added before the mixture was stirred. Several minutes later, the base (0.09 mmol, 1.5 equiv.) was introduced to the flask. After 16 h, the solvent was straight removed under the reduced pressure and the residue was subjected to column chromatography over a silica gel using a mixture of hexane and ethyl acetate as eluent to give the desired compound.

#### (*S*)-4,7,9-Triphenyl-1-oxa-7,8-diazaspiro[4.4]nona-3,8-diene-2,6-dione (**3a**)

Following the general procedure and after column chromatography using hexane/ethyl acetate 10:1 as eluent, **3a** was isolated as white solid (12.1 mg, 54% yield). mp 137–139 °C (from hexane). [α]_D_^25^ = −94.5 (*c* 0.2, CHCl_3_, er 94:6). ^1^H NMR (500 MHz, CDCl_3_): δ 7.97 (d, *J* = 9.8 Hz, 2H), 7.73 (d, *J* = 9.7 Hz, 2H), 7.50–7.42 (m, 4H), 7.41–7.38 (m, 4H), 7.37–7.33 (m, 2H), 7.30 (tt, *J* = 7.5, 1.1 Hz, 1H), 6.77 (s, 1H). ^13^C {^1^H} NMR (126 MHz, CDCl_3_): δ 170.3, 165.4, 162.3, 153.3, 137.2, 132.3, 131.7, 129.7, 129.2, 129.0, 128.1, 126.7, 124.4, 126.2, 119.2, 116.8, 87.3. IR *v*_max_/cm^–1^ 3114, 3059, 2917, 1808, 1779, 1727, 1596, 1497, 1449, 1321, 1175, 1085. HRMS (ESI-TOF) *m*/*z*: calcd for C_24_H_16_N_2_NaO_3_ [M + Na]^+^, 403.1053; found, 403.1062. HPLC (Chiralcel OD, *n*-hexane/2-propanol 80:20, λ = 254 nm, 0.6 mL/min). *t*_R_ (major) = 16.2 min, *t*_R_ (minor) = 27.7 min (er 94:6).

#### (*S*)-9-Methyl-4,7-diphenyl-1-oxa-7,8-diazaspiro[4.4]nona-3,8-diene-2,6-dione (**3b**)

Following the general procedure and after column chromatography using hexane/ethyl acetate 10:1 as eluent, **3b** was isolated as yellow oil (16.7 mg, 66% yield). [α]_D_^25^ = −78.1 (*c* 0.3, CHCl_3_, er 92:8). ^1^H NMR (500 MHz, CDCl_3_): δ 7.88 (d, *J* = 9.8 Hz, 2H), 7.51–7.40 (m, 5H), 7.38–7.36 (m, 2H), 7.27 (tt, *J* = 7.4, 1.1 Hz, 1H), 6.67 (s, 1H), 2.08 (s, 3H). ^13^C {^1^H} NMR (126 MHz, CDCl_3_): δ 170.1, 165.2, 160.1, 156.2, 137.2, 132.4, 129.9, 129.2, 128.0, 126.5, 126.1, 118.9, 116.9, 87.5, 13.3. IR *v*_max_/cm^–1^ 3109, 3062, 2961, 2921, 2849, 1806, 1770, 1727, 1597, 1481, 1366, 1294, 1182, 1121, 1063, 926, 901. HRMS (ESI-TOF) *m*/*z*: calcd for C_19_H_15_N_2_O_3_ [M + H]^+^, 319.1077; found, 319.1083. HPLC (Chiralcel OD, *n*-hexane/2-propanol 80:20, λ = 254 nm, 0.6 mL/min). *t*_R_ (major) = 17.7 min, *t*_R_ (minor) = 27.6 min (er 92:8).

#### (*S*)-9-Ethyl-4,7-diphenyl-1-oxa-7,8-diazaspiro[4.4]nona-3,8-diene-2,6-dione (**3c**)

Following the general procedure and after column chromatography using hexane/ethyl acetate 10:1 as eluent, **3c** was isolated as yellow oil (14.6 mg, 60% yield). [α]_D_^25^ = −10.7 (*c* 0.4, CHCl_3_, er 95:5). ^1^H NMR (500 MHz, CDCl_3_): δ 7.90 (d, *J* = 8.1 Hz, 2H), 7.50–7.35 (m, 7H), 7.27 (t, *J* = 8.0 Hz, 1H), 6.67 (s, 1H), 2.54–2.44 (m, 1H), 2.33–2.23 (m, 1H), 1.22 (t, *J* = 7.8 Hz, 3H). ^13^C {^1^H} NMR (126 MHz, CDCl_3_): δ 170.2, 165.4, 160.3, 160.2, 137.3, 132.4, 129.8, 129.1, 128.1, 126.5, 126.1, 118.9, 116.7, 87.6, 21.3, 9.3. IR *v*_max_/cm^–1^ 3109, 3062, 2957, 1803, 1781, 1730, 1590, 1575, 1489, 1453, 1348, 1258, 1182, 1124, 1038, 753. HRMS (ESI-TOF) *m*/*z*: calcd for C_20_H_16_KN_2_O_3_ [M + K]^+^, 371.0793; found, 371.0799. HPLC (Chiralcel OD, *n*-hexane/2-propanol 85:15, λ = 210 nm, 1 mL/min). *t*_R_ (major) = 10.7 min, *t*_R_ (minor) = 18.6 min (er 95:5).

#### (*S*)-9-Isopropyl-4,7-diphenyl-1-oxa-7,8-diazaspiro[4.4]nona-3,8-diene-2,6-dione (**3d**)

Following the general procedure and after column chromatography using hexane/ethyl acetate 10:1 as eluent, **3d** was isolated as yellow oil (17.1 mg, 81% yield). [α]_D_^25^ = −74.8 (*c* 0.3, CHCl_3_, er 95:5). ^1^H NMR (500 MHz, CDCl_3_): δ 7.91 (d, *J* = 8.8 Hz, 2H), 7.49–7.35 (m, 7H), 7.27 (t, *J* = 8.0 Hz, 1H), 6.69 (s, 1H), 2.70–2.60 (m, 1H), 1.26 (d, *J* = 6.9 Hz, 3H), 1.10 (d, *J* = 7.0 Hz, 3H). ^13^C {^1^H} NMR (126 MHz, CDCl_3_): δ 170.3, 165.5, 163.3, 160.1, 137.3, 132.4, 129.8, 129.1, 128.3, 126.5, 126.1, 119.0, 116.5, 87.8, 29.2, 20.2. IR *v*_max_/cm^–1^ 3058, 2964, 2928, 1808, 1774, 1730, 1597, 1500, 1377, 1344, 1328, 1240, 1186, 1124, 1045. HRMS (ESI-TOF) *m*/*z*: calcd for C_21_H_18_N_2_NaO_3_ [M + Na]^+^, 369.1210; found, 369.1216. HPLC (Chiralcel OD, *n*-hexane/2-propanol 90:10, 0.8 mL/min, λ = 210 nm). *t*_R_ (major) = 13.3 min, *t*_R_ (minor) = 29.9 min (er 95:5).

#### (*S*)-9-(*tert*-Butyl)-4,7-diphenyl-1-oxa-7,8-diazaspiro[4.4]nona-3,8-diene-2,6-dione (**3e**)

Following the general procedure and after column chromatography using hexane/ethyl acetate 10:1 as eluent, **3e** was isolated as yellow oil (17.8 mg, 76% yield). [α]_D_^25^ = −103.7 (*c* 0.4, CHCl_3_, er 89:11). ^1^H NMR (500 MHz, CDCl_3_): δ 7.88 (d, *J* = 8.6 Hz, 2H), 7.49–7.35 (m, 7H), 7.27 (t, *J* = 8.7 Hz, 1H), 6.69 (s, 1H), 1.20 (s, 9H). ^13^C {^1^H} NMR (126 MHz, CDCl_3_): δ 170.3, 165.6, 164.6, 162.0, 137.3, 132.3, 129.7, 129.1, 128.6, 126.5, 126.1, 118.9, 116.2, 88.1, 36.9, 28.6. IR *v*_max_/cm^–1^ 3098, 2972, 2932, 1817, 1770, 1723, 1601, 1500, 1362, 1294, 1207, 1175, 1067, 1023, 955. HRMS (ESI-TOF) *m*/*z*: calcd for C_22_H_20_N_2_NaO_3_ [M + Na]^+^, 383.1366; found, 383.1372. HPLC (Chiralcel OD, *n*-hexane/2-propanol 80:20, λ = 254 nm, 0.6 mL/min). *t*_R_ (major) = 11.6 min, *t*_R_ (minor) = 22.9 min (er 89:11).

#### (*S*)-9-(Naphthalen-2-yl)-4,7-diphenyl-1-oxa-7,8-diazaspiro[4.4]nona-3,8-diene-2,6-dione (**3f**)

Following the general procedure and after column chromatography using hexane/ethyl acetate 10:1 as eluent, **3f** was isolated as pale yellow oil (11.2 mg, 52% yield). [α]_D_^25^ = −70.1 (*c* 0.2, CHCl_3_, er 96:4). ^1^H NMR (500 MHz, CDCl_3_): δ 8.01–7.98 (m, 4H), 7.87 (d, *J* = 8.6 Hz, 1H), 7.81 (t, *J* = 8.5 Hz, 2H), 7.56–7.48 (m, 4H), 7.42–7.38 (m, 3H), 7.35–7.30 (m, 3H), 6.8 (s, 1H). ^13^C {^1^H} NMR (126 MHz, CDCl_3_): δ 170.3, 165.4, 162.6, 153.1, 137.2, 134.6, 132.8, 132.3, 129.7, 129.2, 129.2, 129.0, 128.2, 128.1, 127.8, 127.0, 126.8, 126.7, 126.5, 126.4, 122.5, 119.2, 116.8, 87.4. IR *v*_max_/cm^–1^ 3066, 2954, 2925, 2860, 1803, 1770, 1730, 1597, 1453, 1308, 1175, 1128, 1059, 966. HRMS (ESI-TOF) *m*/*z*: calcd forC_28_H_18_N_2_NaO_3_ [M + Na]^+^, 453.1210; found, 453.1222. HPLC (Chiralcel OD, *n*-hexane/2-propanol 80:20, λ = 254 nm, 0.6 mL/min). *t*_R_ (major) = 18.3 min, *t*_R_ (minor) = 32.3 min (er 96:4).

#### (*S*)-7-Methyl-4,9-diphenyl-1-oxa-7,8-diazaspiro[4.4]nona-3,8-diene-2,6-dione (**3g**)

Following the general procedure and after column chromatography using hexane/ethyl acetate 10:1 as eluent, **3g** was isolated as yellow oil (11.6 mg, 46% yield). [α]_D_^25^ = −72.0 (*c* 0.2, CHCl_3_, er 90:10). ^1^H NMR (500 MHz, CDCl_3_): δ 7.60 (d, *J* = 9.8 Hz, 2H), 7.45–7.33 (m, 8H), 6.70 (s, 1H), 3.51 (s, 3H). ^13^C {^1^H} NMR (126 MHz, CDCl_3_): δ 170.4, 167.3, 162.4, 151.6, 132.2, 131.3, 129.6, 129.1, 128.2, 126.7, 125.9, 116.7, 86.2, 32.5. IR *v*_max_/cm^–1^ 3109, 3069, 2925, 2853, 1808, 1770, 1727, 1604, 1445, 1391, 1341, 1229, 1095, 1063, 864. HRMS (ESI-TOF) *m*/*z*: calcd for C_19_H_14_N_2_NaO_3_ 1579, [M + Na]^+^, 341.0897; found, 341.0907. HPLC (Lux Amylose-1, *n*-hexane/2-propanol 80:20, λ = 254 nm, 0.6 mL/min). *t*_R_ (minor) = 25.1 min, *t*_R_ (major) = 39.0 min (er 90:10).

#### (*S*)-9-(*tert*-Butyl)-7-methyl-4-phenyl-1-oxa-7,8-diazaspiro[4.4]nona-3,8-diene-2,6-dione (**3h**)

Following the general procedure and after column chromatography using hexane/ethyl acetate 10:1 as eluent, **3h** was isolated as pale yellow oil (13.3 mg, 50% yield). [α]_D_^25^ = −14.3 (*c* 0.3, CHCl_3_, er 88:12). ^1^H NMR (500 MHz, CDCl_3_): δ 7.50–7.40 (m, 3H), 7.33 (d, *J* = 9.7 Hz, 2H), 6.63 (s, 1H), 3.43 (s, 3H), 1.12 (s, 9H). ^13^C {^1^H} NMR (126 MHz, CDCl_3_): δ 170.5, 167.7, 163.9, 162.0, 132.2, 129.6, 128.7, 126.5, 116.0, 87.0, 36.6, 32.2, 28.6. IR *v*_max_/cm^–1^ 3120, 2972, 2936, 2878, 1803, 1777, 1727, 1611, 1500, 1449, 1399, 1283, 1204, 1164, 1121, 1045. HRMS (ESI-TOF) *m*/*z*: calcd for C_17_H_18_N_2_NaO_3_ [M + Na]^+^, 321.1210; found, 321.1216. HPLC (Chiralpak AD-H, *n*-hexane/2-propanol 90:10, λ = 254 nm, 0.7 mL/min). *t*_R_ (minor) = 17.2 min, *t*_R_ (major) = 20.3 min (er 88:12).

#### (*S*)-7,9-Diphenyl-4-(*p*-tolyl)-1-oxa-7,8-diazaspiro[4.4]nona-3,8-diene-2,6-dione (**3ab**)

Following the general procedure and after column chromatography using hexane/ethyl acetate 10:1 as eluent, **3ab** was isolated as pale yellow oil (11.9 mg, 51% yield). [α]_D_^25^ = −134.9 (*c* 0.2, CHCl_3_, er 96:4). ^1^H NMR (500 MHz, CDCl_3_): δ 7.97 (d, *J* = 7.1 Hz, 2H), 7.72 (d, *J* = 8.1 Hz, 2H), 7.50–7.43 (m, 4H), 7.40–7.36 (m, 2H), 7.32–7.29 (m, 2H), 7.15 (d, *J* = 7.8 Hz, 2H), 6.73 (s, 1H), 2.30 (s, 3H). ^13^C {^1^H} NMR (126 MHz, CDCl_3_): δ 170.5, 165.5, 162.2, 153.5, 143.3, 137.3, 131.7, 130.5, 129.2, 129.1, 129.0, 126.6, 126.3, 126.2, 125.3, 119.1, 115.6, 87.2, 21.5. IR *v*_max_/cm^–1^ 3065, 2953, 2921, 2856, 1803, 1774, 1737, 1593, 1492, 1384, 1319, 1182, 1142, 1063. HRMS (ESI-TOF) *m*/*z*: calcd forC_25_H_18_N_2_NaO_3_ [M + Na]^+^, 417.1210; found, 417.1224. HPLC (Chiralcel OD, *n*-hexane/2-propanol 95:5, λ = 254 nm, 0.8 mL/min). *t*_R_ (major) = 25.7 min, *t*_R_ (minor) = 30.5 min (er 96:4).

#### (*S*)-4-(4-Methoxyphenyl)-7,9-diphenyl-1-oxa-7,8-diazaspiro[4.4]nona-3,8-diene-2,6-dione (**3ac**)

Following the general procedure and after column chromatography using hexane/ethyl acetate 10:1 as eluent, **3ac** was isolated as pale pink solid (8.1 mg, 33% yield). mp 67–69 °C (from hexane). [α]_D_^25^ = −120.9 (*c* 0.2, CHCl_3_, er 86:14). ^1^H NMR (500 MHz, CDCl_3_): δ 7.98 (d, *J* = 8.8 Hz, 2H), 7.72 (d, *J* = 8.4 Hz, 2H), 7.51–7.43 (m, 3H), 7.40–7.36 (m, 4H), 7.30 (t, *J* = 7.5 Hz, 1H), 6.84 (d, *J* = 8.0 Hz, 2H), 6.65 (s, 1H), 3.76 (s, 3H). ^13^C {^1^H} NMR (126 MHz, CDCl_3_): δ 170.6, 165.6, 162.8, 161.6, 153.8, 137.3, 131.7, 129.2, 129.0, 128.6, 126.1, 120.6, 119.1, 115.2, 114.0, 87.0, 55.5. IR *v*_max_/cm^–1^ 3105, 3062, 2961, 2918, 2853, 1803, 1781, 1727, 1597, 1496, 1316, 1240, 1063, 901. HRMS (ESI-TOF) *m*/*z*: calcd forC_25_H_18_N_2_NaO_3_ [M + Na]^+^, 433.1159; found, 433.1169. HPLC (Lux i-Cellulose-5, *n*-hexane/2-propanol 70:30, λ = 254 nm, 0.6 mL/min). *t*_R_ (minor) = 47.9 min, *t*_R_ (major) = 81.0 min (er 86:14).

#### (*S*)-4-(2-Methoxyphenyl)-7,9-diphenyl-1-oxa-7,8-diazaspiro[4.4]nona-3,8-diene-2,6-dione (**3ad**)

Following the general procedure and after column chromatography using hexane/ethyl acetate 10:1 as eluent, **3ad** was isolated as white solid (5.6 mg, 23% yield). mp 38–40 °C (from hexane). ^1^H NMR (500 MHz, CDCl_3_): δ 8.01 (d, *J* = 7.7 Hz, 2H), 7.68 (d, *J* = 9.7 Hz, 2H), 7.48 (t, *J* = 7.6 Hz, 1H), 7.42–7.32 (m, 5H), 7.28 (t, *J* = 8.5 Hz, 1H), 7.03 (s, 1H), 6.89 (d, *J* = 8.1 Hz, 2H), 3.71 (s, 3H). ^13^C {^1^H} NMR (126 MHz, CDCl_3_): δ 170.8, 165.8, 158.3, 157.9, 153.2, 137.6, 133.5, 131.3, 129.2, 129.0, 128.5, 126.2, 125.9, 121.4, 119.7, 118.8, 117.6, 111.8, 83.0, 55.1. IR *v*_max_/cm^–1^ 3077, 2956, 2923, 1861, 1806, 1780, 1725, 1600, 1491, 1465, 1384, 1263, 1139, 1025, 941, 754. HRMS (ESI-TOF) *m*/*z*: calcd forC_25_H_18_N_2_NaO_3_ [M + Na]^+^, 433.1159; found, 433.1163. HPLC (Lux i-Cellulose-5, *n*-hexane/2-propanol 70:30, λ = 254 nm, 0.6 mL/min). *t*_R_ = 57.8 min, 63.9 min (er 53:47).

#### (*S*)-4-(4-Fluorophenyl)-7,9-diphenyl-1-oxa-7,8-diazaspiro[4.4]nona-3,8-diene-2,6-dione (**3ae**)

Following the general procedure and after column chromatography using hexane/ethyl acetate 10:1 as eluent, **3ae** was isolated as yellow oil (11.9 mg, 51% yield). [α]_D_^25^ = −13.8 (*c* 0.1, CHCl_3_, er 96:4). ^1^H NMR (500 MHz, CDCl_3_): δ 7.95 (d, *J* = 9.8 Hz, 2H), 7.71 (d, *J* = 7.2 Hz, 2H), 7.50–7.45 (m, 3H), 7.45–7.38 (m, 4H), 7.31 (t, *J* = 7.4 Hz, 1H), 7.05 (t, *J* = 8.4 Hz, 2H), 6.71 (s, 1H). ^13^C {^1^H} NMR (126 MHz, CDCl_3_): δ 170.0, 165.5 (d, *J* = 177.2 Hz), 165.2, 163.8, 153.2, 137.1, 131.8, 129.3, 129.2, 129.0, 128.9, 128.8, 126.5, 126.2, 124.4, 119.1, 117.2, 117.1, 116.7, 87.2. ^19^F NMR (470 MHz, CDCl_3_): δ −106.3. IR *v*_max_/cm^–1^ 3069, 2957, 2925, 2853, 1777, 1734, 1604, 1492, 1445, 1387, 1319, 1294, 1233, 1164, 1142. HRMS (ESI-TOF) *m*/*z*: calcd forC_24_H_16_FN_2_O_3_ [M + H]^+^, 399.1139; found, 399.1147. HPLC (Lux i-Cellulose-5, *n*-hexane/2-propanol 80:20, λ = 230 nm, 0.6 mL/min). *t*_R_ (minor) = 36.7 min, *t*_R_ (major) = 56.2 min (er 96:4).

#### (*S*)-4-(4-Chlorophenyl)-7,9-diphenyl-1-oxa-7,8-diazaspiro[4.4]nona-3,8-diene-2,6-dione (**3af**)

Following the general procedure and after column chromatography using hexane/ethyl acetate 10:1 as eluent, **3af** was isolated as white solid (17 mg, 69% yield). mp 52–54 °C (from hexane). [α]_D_^25^ = −157.7 (*c* 0.1, CHCl_3_, er 93:7). ^1^H NMR (500 MHz, CDCl_3_): δ 7.95 (d, *J* = 9.6 Hz, 2H), 7.70 (d, *J* = 9.7 Hz, 2H), 7.50–7.45 (m, 3H), 7.40 (t, *J* = 6.6 Hz, 1H), 7.35–7.29 (m, 5H), 6.75 (s, 1H). ^13^C {^1^H} NMR (126 MHz, CDCl_3_): δ 169.9, 165.1, 161.0, 153.1, 138.7, 137.1, 131.9, 130.1, 129.3, 129.2, 128.8, 127.9, 126.6, 126.5, 126.2, 119.1, 117.3, 87.1. IR *v*_max_/cm^–1^ 2968, 2925, 2860, 1806, 1774, 1734, 1591, 1489, 1388, 1323, 1301, 1178, 1142. HRMS (ESI-TOF) *m*/*z*: calcd for C_24_H_15_ClN_2_NaO_3_ [M + Na]^+^, 437.0663; found, 437.0674. HPLC (Chiralcel OD, *n*-hexane/2-propanol 95:5, λ = 254 nm, 0.8 mL/min). *t*_R_ (minor) = 26.0 min, *t*_R_ (major) = 32.9 min (er 93:7).

#### (*S*)-4-(4-Bromophenyl)-7,9-diphenyl-1-oxa-7,8-diazaspiro[4.4]nona-3,8-diene-2,6-dione (**3ag**)

Following the general procedure and after column chromatography using hexane/ethyl acetate 10:1 as eluent, **3ag** was isolated as yellow oil (23.9 mg, 88% yield). [α]_D_^25^ = −52.7 (*c* 0.5, CHCl_3_, er 92:8). ^1^H NMR (500 MHz, CDCl_3_): δ 7.95 (d, *J* = 7.9 Hz, 2H), 7.70 (d, *J* = 9.0 Hz, 2H), 7.51–7.42 (m, 5H), 7.40–7.38 (m, 2H), 7.31 (t, *J* = 7.5 Hz, 1H), 7.24–7.23 (m, 1H), 6.76 (s, 1H). ^13^C {^1^H} NMR (126 MHz, CDCl_3_): δ 169.9, 165.1, 161.1, 153.0, 137.1, 133.1, 131.9, 129.3, 129.2, 128.8, 128.0, 127.2, 127.0, 126.5, 126.1, 119.1, 117.3, 87.1. IR *v*_max_/cm^–1^ 3063, 2960, 2923, 2857, 1806, 1777, 1733, 1593, 1494, 1387, 1299, 1178, 1141, 1071, 1009. HRMS (ESI-TOF) *m*/*z*: calcd forC_24_H_15_BrN_2_NaO_3_ [M + Na]^+^, 481.0158; found, 481.0169. HPLC (Chiralcel OD, *n*-hexane/2-propanol 85:15, λ = 254 nm, 0.8 mL/min). *t*_R_ (minor) = 23.8 min, *t*_R_ (major) = 32.4 min (er 92:8).

#### (*S*)-4-(4-Nitrophenyl)-7,9-diphenyl-1-oxa-7,8-diazaspiro[4.4]nona-3,8-diene-2,6-dione (**3ah**)

Following the general procedure and after column chromatography using hexane/ethyl acetate 10:1 as eluent, **3ah** was isolated as white solid (10.6 mg, 42% yield). mp 48–50 °C (from hexane). [α]_D_^25^ = −30.7 (*c* 0.2, CHCl_3_, er 92:8). ^1^H NMR (500 MHz, CDCl_3_): δ 8.02 (d, *J* = 8.8 Hz, 2H), 7.94 (d, *J* = 9.7 Hz, 2H), 7.71 (d, *J* = 9.5 Hz, 2H), 7.53 (d, *J* = 8.3 Hz, 2H), 7.49 (t, *J* = 7.3 Hz, 3H), 7.42 (t, *J* = 7.7 Hz, 2H), 7.32 (d, *J* = 7.4 Hz, 1H), 6.89 (s, 1H). ^13^C {^1^H} NMR (126 MHz, CDCl_3_): δ 169.1, 164.7, 159.8, 152.4, 149.5, 136.9, 133.8, 132.1, 129.4, 129.3, 128.6, 127.8, 126.7, 126.1, 124.8, 120.4, 119.0, 87.3. IR *v*_max_/cm^–1^ 3109, 1815, 1779, 1717, 1598, 1525, 1489, 1348, 1323, 1178, 1138, 1058, 909. HRMS (ESI-TOF) *m*/*z*: calcd for C_24_H_15_N_3_NaO_5_ [M + Na]^+^, 448.0904; found, 448.0915. HPLC (Chiralcel OD, *n*-hexane/2-propanol 80:20, λ = 254 nm, 0.6 mL/min). *t*_R_ (minor) = 33.6 min, *t*_R_ (major) = 50.4 min (er 92:8).

#### (*S*)-4-(3-Nitrophenyl)-7,9-diphenyl-1-oxa-7,8-diazaspiro[4.4]nona-3,8-diene-2,6-dione (**3ai**)

Following the general procedure and after column chromatography using hexane/ethyl acetate 10:1 as eluent, **3ai** was isolated as white solid (13.4 mg, 43% yield). mp 184–186 °C (from hexane/acetate). [α]_D_^25^ = – 35.8 (*c* 0.2, CHCl_3_, er 95:5). ^1^H NMR (500 MHz, CDCl_3_): δ 8.29–8.27 (m, 2H), 7.75–7.73 (m, 2H), 7.70–7.67 (m, 1H), 7.61–7.58 (m, 1H), 7.58–7.52 (m, 4H), 7.45–7.42 (m, 3H), 7.31 (tt, *J* = 7.1, 1.2 Hz, 1H), 6.90 (s, 1H). ^13^C {^1^H} NMR (126 MHz, CDCl_3_): δ 169.2, 164.8, 159.6, 152.6, 148.8, 136.9, 132.1, 131.0, 130.6, 129.6, 129.4, 129.1, 128.7, 128.7, 126.8, 126.5, 121.7, 119.5, 119.4, 119.2, 87.2. IR *v*_max_/cm^–1^ 2971, 2923, 2846, 1776, 1732, 1597, 1538, 1487, 1381, 1381, 1351, 1326, 1139, 1069, 956. HRMS (ESI-TOF) *m*/*z*: calcd for C_24_H_15_N_3_NaO_5_ [M + Na]^+^, 448.0904; found, 448.0899. HPLC (Chiralcel OD, *n*-hexane/2-propanol 75:25, λ = 254 nm, 1 mL/min). *t*_R_ (major) = 17.0 min, *t*_R_ (minor) = 20.3 min (er 95:5).

#### (*S*)-4-(Furan-2-yl)-7,9-diphenyl-1-oxa-7,8-diazaspiro[4.4]nona-3,8-diene-2,6-dione (**3aj**)

Following the general procedure and after column chromatography using hexane/ethyl acetate 10:1 as eluent, **3aj** was isolated as white solid (14.8 mg, 68% yield). mp 155–157 °C (from hexane). [α]_D_^25^ = −84.5 (*c* 0.2, CHCl_3_, er 97:3). ^1^H NMR (500 MHz, CDCl_3_): δ 7.98 (d, *J* = 8.5 Hz, 2H), 7.71 (d, *J* = 7.9 Hz, 2H), 7.53–7.44 (m, 4H), 7.42–7.38 (m, 2H), 7.31 (t, *J* = 6.5 Hz, 1H), 6.70 (s, 1H), 6.60 (s, 1H), 6.43 (s, 1H). ^13^C {^1^H} NMR (126 MHz, CDCl_3_): δ 170.5, 165.4, 153.4, 150.2, 147.1, 143.6, 131.7, 131.2, 129.2, 129.1, 128.8, 126.3, 126.2, 119.1, 115.1, 113.3, 112.0, 85.6. IR *v*_max_/cm^–1^ 3148, 3115, 3075, 2957, 2932, 2862, 1797, 1775, 1735, 1628, 1592, 1500, 1383, 1316, 1170, 1070, 1026, 905. HRMS (ESI-TOF) *m*/*z*: calcd for C_22_H_15_N_2_O_4_ [M + Na]^+^, 371.1026; found, 371.1030. HPLC (Chiralcel IA, *n*-hexane/2-propanol 90:10, λ = 254 nm, 0.5 mL/min). *t*_R_ (major) = 42.9 min, *t*_R_ (minor) = 147.7 min (er 97:3).

#### (*S*)-7,9-Diphenyl-4-(thiophen-2-yl)-1-oxa-7,8-diazaspiro[4.4]nona-3,8-diene-2,6-dione (**3ak**)

Following the general procedure and after column chromatography using hexane/ethyl acetate 10:1 as eluent, **3ak** was isolated as white solid (15.0 mg, 66% yield). mp 130–132 °C (from hexane). [α]_D_^25^ = −150.7 (*c* 0.3, CHCl_3_, er 92:8). ^1^H NMR (500 MHz, CDCl_3_): δ 7.98 (d, *J* = 9.3 Hz, 2H), 7.74 (d, *J* = 8.8 Hz, 2H), 7.52–7.45 (m, 4H), 7.24–7.38 (m, 2H), 7.31 (t, *J* = 7.3 Hz, 1H), 7.23 (d, *J* = 3.8 Hz, 1H), 7.01 (dd, *J* = 4.7, 3.9 Hz, 1H), 6.57 (s, 1H). ^13^C {^1^H} NMR (126 MHz, CDCl_3_): δ 170.2, 165.3, 155.2, 153.6, 137.2, 132.0, 131.8, 130.6, 129.4, 129.3, 129.2, 129.1, 128.8, 126.4, 126.2, 119.1, 113.6, 86.7. IR *v*_max_/cm^–1^ 3115, 2965, 2921, 2851, 1801, 1768, 1742, 1592, 1482, 1386, 1173, 1148, 1085, 1067, 946. HRMS (ESI-TOF) *m*/*z*: calcd for C_22_H_14_N_2_NaO_3_S [M + Na]^+^, 409.0617; found, 409.0624. HPLC (Chiralcel OD, *n*-hexane/2-propanol 95:5, λ = 254 nm, 0.5 mL/min). *t*_R_ (major) = 56.3 min, *t*_R_ (minor) = 63.2 min (er 92:8).

#### (*S*)-7,9-Diphenyl-4-styryl-1-oxa-7,8-diazaspiro[4.4]nona-3,8-diene-2,6-dione (**3al**)

Following the general procedure and after column chromatography using hexane/ethyl acetate 10:1 as eluent, **3al** was isolated as pale yellow oil (8.5 mg, 18% yield). [α]_D_^25^ = −75.7 (*c* 0.2, CHCl_3_, er 78:22). ^1^H NMR (500 MHz, CDCl_3_): δ 8.00 (d, *J* = 9.8 Hz, 2H), 7.73 (d, *J* = 9.7 Hz, 2H), 7.52–7.46 (m, 3H), 7.42 (t, *J* = 8.3 Hz, 2H), 7.33–7.30 (m, 6H), 6.94 (d, *J* = 16.5 Hz, 1H), 6.77 (dd, *J* = 16.5, 0.7 Hz, 1H), 6.48 (s, 1H). ^13^C {^1^H} NMR (126 MHz, CDCl_3_): δ 170.7, 165.5, 159.5, 153.6, 140.0, 137.2, 134.3, 131.8, 130.6, 129.3, 129.2, 129.0, 128.8, 127.8, 126.4, 126.2, 119.2, 116.3, 115.8, 87.0. IR *v*_max_/cm^–1^ 2924, 2854, 1804, 1774, 1727, 1621, 1588, 1494, 1315, 1293, 1169, 1143, 1070, 899. HRMS (ESI-TOF) *m*/*z*: calcd for C_26_H_18_N_2_NaO_3_ [M + H]^+^, 429.1210; found, 429.1219. HPLC (Chiralcel OD, *n*-hexane/2-propanol 90:10, λ = 220 nm, 0.5 mL/min). *t*_R_ (major) = 36.3 min, *t*_R_ (minor) = 28.7 min (er 78:22).

#### (*S*)-9-Ethyl-4-(4-fluorophenyl)-7-phenyl-1-oxa-7,8-diazaspiro[4.4]nona-3,8-diene-2,6-dione (**3ce**)

Following the general procedure and after column chromatography using hexane/ethyl acetate 10:1 as eluent, **3ce** was isolated as yellow oil (9.2 mg, 35% yield). [α]_D_^25^ = −45.7 (*c* 0.2, CHCl_3_, er 92:8). ^1^H NMR (500 MHz, CDCl_3_): δ 7.90 (d, *J* = 8.8 Hz, 2H), 7.46 (t, *J* = 7.7 Hz, 2H), 7.39–7.36 (m, 2H), 7.28 (t, *J* = 7.3 Hz, 1H), 7.11 (t, *J* = 8.2 Hz, 2H), 6.61 (s, 1H), 2.54–2.44 (m, 1H), 2.31–2.23 (m, 1H), 1.22 (t, *J* = 7.5 Hz, 3H). ^13^C {^1^H} NMR (126 MHz, CDCl_3_): δ 170.0, 166.2, 164.5 (d, *J* = 177.2 Hz), 160.2, 159.1, 137.2, 129.2, 128.9, 128.8, 126.2, 124.4, 118.9, 117.4, 117.1, 116.5, 87.5, 21.3, 9.3. ^19^F NMR (470 MHz, CDCl_3_): δ −106.4. IR *v*_max_/cm^–1^ 2986, 2913, 2855, 1808, 1783, 1728, 1598, 1500, 1457, 1391, 1348, 1239, 1181, 1163, 1120, 1051. HRMS (ESI-TOF) *m*/*z*: calcd for C_20_H_15_FN_2_NaO_3_ [M + Na]^+^, 373.0959; found, 373.0961. HPLC (Chiralcel OD, *n*-hexane/2-propanol 95:5, λ = 254 nm, 1 mL/min). *t*_R_ (minor) = 21.3 min, *t*_R_ (major) = 24.6 min (er 92:8).

#### (*S*)-9-Isopropyl-7-phenyl-4-(*p*-tolyl)-1-oxa-7,8-diazaspiro[4.4]nona-3,8-diene-2,6-dione (**3db**)

Following the general procedure and after column chromatography using hexane/ethyl acetate 10:1 as eluent, **3db** was isolated as yellow oil (18.8 mg, 86% yield). [α]_D_^25^ = −93.0 (*c* 0.4, CHCl_3_, er 93:7). ^1^H NMR (500 MHz, CDCl_3_): δ 7.90 (d, *J* = 9.8 Hz, 2H), 7.46 (t, *J* = 7.4 Hz, 2H), 7.29–7.25 (m, 3H), 7.21–7.19 (m, 2H), 6.63 (s, 1H), 2.70–2.61 (m, 1H), 2.35 (s, 3H), 1.26 (d, *J* = 6.9 Hz, 3H), 1.10 (d, *J* = 7.0 Hz, 3H). ^13^C {^1^H} NMR (126 MHz, CDCl_3_): δ 170.5, 165.3, 163.5, 160.8, 143.3, 137.4, 130.5, 129.1, 126.5, 126.0, 125.5, 118.9, 115.3, 87.7, 29.1, 21.5, 20.2. IR *v*_max_/cm^–1^ 2975, 2874, 1803, 1774, 1727, 1597, 1500, 1380, 1326, 1182, 1128, 1049, 904. HRMS (ESI-TOF) *m*/*z*: calcd for C_22_H_20_N_2_NaO_3_ [M + Na]^+^, 383.1366; found, 383.1378. HPLC (Lux i-Cellulose-5, *n*-hexane/2-propanol 80:20, λ = 210 nm, 0.8 mL/min). *t*_R_ (minor) = 28.1 min, *t*_R_ (major) = 38.9 min (er 93:7).

#### (*S*)-4-(4-chlorophenyl)-9-isopropyl-7-phenyl-1-oxa-7,8-diazaspiro[4.4]nona-3,8-diene-2,6-dione (**3df**)

Following the general procedure and after column chromatography using hexane/ethyl acetate 10:1 as eluent, **3df** was isolated as yellow oil (16.6 mg, 71% yield). [α]_D_^25^ = −65.1 (*c* 0.3, CHCl_3_, er 91:9). ^1^H NMR (500 MHz, CDCl_3_): δ 7.89 (d, *J* = 9.5 Hz, 2H), 7.46 (t, *J* = 7.6 Hz, 2H), 7.40–7.38 (m, 2H), 7.30–7.28 (m, 3H), 6.67 (s, 1H), 2.70–2.60 (m, 1H), 1.26 (d, *J* = 6.9 Hz, 3H), 1.11 (d, *J* = 6.9 Hz, 3H). ^13^C {^1^H} NMR (126 MHz, CDCl_3_): δ 170.0, 165.3, 163.1, 159.4, 138.8, 137.2, 130.2, 129.2, 127.8, 126.7, 126.2, 118.9, 116.9, 87.7, 29.2, 20.2. IR *v*_max_/cm^–1^ 3108, 2979, 2939, 2880, 1808, 1772, 1731, 1588, 1489, 1375, 1342, 1181, 1093, 927. HRMS (ESI-TOF) *m*/*z*: calcd for C_21_H_17_ClN_2_NaO_3_ [M + Na]^+^, 403.0820; found, 403.0821. HPLC (Lux i-Cellulose-5, *n*-hexane/2-propanol 80:20, λ = 210 nm, 0.6 mL/min). *t*_R_ (minor) = 31.9 min, *t*_R_ (major) = 47.8 min (er 91:9).

### Procedure for Scale-Up Synthesis of Spiropyrazolone-butenolide **3ag**

In a-50 mL heat gun-dried flask equipped with a magnetic stirring bar, the precatalyst **C2** (42 mg, 0.1 mmol, 0.1 equiv) and the pyrazolin-4,5-dione **1a** (254 mg, 1 mmol) were weighed. Then, β-bromoenal **2g** (435 mg, 1.5 mmol, 1.5 equiv) was added under a N_2_ atmosphere. Dry chloroform (15 mL) was added before the mixture was stirred. Several minutes later, the base (0.24 mL, 1.5 mmol, 1.5 equiv) was introduced to the flask. After 16 h, the solvent was straight removed under the reduced pressure and the residue was subjected to column chromatography over a silica gel using hexane/ethyl acetate 10:1 as eluent to give **3ag**, as a yellow oil (367 mg, 80% yield).

### Catalytic Hydrogenation of Spiropyrazolone-butenolide **3a**

To a solution of spirocyclic butenolide **3a** (34.8 mg, 0.091 mmol) in ethyl acetate (2 mL), Pd/C (10 wt %) was added. The mixture was stirred under hydrogen atmospheric pressure for 20 h. After the removal of the palladium on carbon, the solvent was removed and the crude product was purified by a column chromatography (hexane/ethyl acetate 10:1) affording compound **4** as a single diastereomer (15.7 mg, 45% yield).

#### (4*R*,5*S*)-4,7,9-Triphenyl-1-oxa-7,8-diazaspiro[4.4]non-8-ene-2,6-dione (**4**)

Analytical data were consistent with the reported data.^12^^1^H NMR (500 MHz, CDCl_3_): δ 7.98–8.00 (m, 2H), 7.54–7.59 (m, 3H), 7.43–7.45 (m, 2H), 7.22–7.31 (m, 5H), 7.14–7.18 (m, 1H), 7.11–7.13 (m, 2H), 4.15 (dd, *J* = 13.8, 8.2 Hz, 1H), 3.82 (dd, *J* = 14.0, 13.8 Hz, 1H), 2.93 (dd, *J* = 17.1, 8.2 Hz, 1H). ^13^C {^1^H} NMR (126 MHz, CDCl_3_): δ 173.5, 168.7, 153.2, 136.5, 131.5, 130.5, 129.4, 129.1, 128.8, 128.7, 128.6, 127.6, 126.8, 126.1, 119.5, 87.8, 49.0, 30.3. HPLC (Lux Amylose-1, *n*-hexane/2-propanol 70:30, λ = 254 nm, 0.8 mL/min): *t*_R_ (minor) = 6.4 min, *t*_R_ (major) = 7.8 min (er 94:6).

### Transformation of Spiropyrazolone-butenolide **3ag**

To a solution of spirocyclic butenolide **3ag** (38.3 mg, 0.083 mmol), phenylboronic acid (15.3 mg, 0.125 mmol), and K_3_PO_4_ (35.3 mg, 0.166 mmol) in THF/H_2_O 5:1 (1.2 mL) under a N_2_ atmosphere, PdCl_2_(PPh_3_)_2_ (10 mol %) was added. After refluxing for 8 h, the solvent was removed under the reduced pressure. The crude mixture was purified by a column chromatography (hexane/ethyl acetate 10:1) affording **5** as a white solid (36.8 mg, 96% yield).

#### (*S*)-4-([1,1′-Biphenyl]-4-yl)-7,9-diphenyl-1-oxa-7,8-diazaspiro[4.4]nona-3,8-diene-2,6-dione (**5**)

mp 66–68 °C (from hexane/acetate). [α]_D_^25^ = −116.0 (*c* 0.4, CHCl_3_, er 95:5). ^1^H NMR (500 MHz, CDCl_3_): δ 7.99 (d, *J* = 8.8 Hz, 2H), 7.75 (d, *J* = 8.1 Hz, 2H), 7.58 (d, *J* = 8.3 Hz, 2H), 7.52–7.48 (m, 5H), 7.47–7.46 (m, 2H), 7.44–7.37 (m, 5H), 7.31 (t, *J* = 8.0 Hz, 1H), 6.80 (s, 1H). ^13^C {^1^H} NMR (126 MHz, CDCl_3_): δ 170.3, 165.4, 161.7, 153.5, 145.2, 139.1, 137.3, 133.1, 131.8, 129.2, 129.0, 128.4, 128.3, 127.0, 126.8, 126.4, 126.3, 119.2, 116.3, 81.2. IR *v*_max_/cm^–1^ 3073, 3030, 2927, 2851, 1804, 1774, 1723, 1599, 1559, 1442, 1384, 1180, 1143, 1070, 1005. HRMS (ESI-TOF) *m*/*z*: calcd for C_30_H_21_N_2_O_3_ [M + H]^+^, 457.1547; found, 457.1528. HPLC (Lux i-Cellulose 5, *n*-hexane/2-propanol 85:15, λ = 254 nm, 0.8 mL/min). *t*_R_ (minor) = 56.4 min, *t*_R_ (major) = 85.4 min (er 95:5).

### Procedure for Spirooxindole-butenolide **7**

In a 5-mL heat gun-dried flask equipped with a magnetic stirring bar, the precatalyst **C2** (6 μmol, 0.1 equiv) and *N*-benzyl isatin **6** (0.06 mmol) were weighed. Then, β-bromoenal **2a** (0.09 mmol, 1.5 equiv) was added under a N_2_ atmosphere. Dry chloroform (1 mL) was added before the mixture was stirred. Several minutes later, DBU (0.09 mmol, 1.5 equiv) was introduced to the flask. After 16 h, the solvent was straight removed under the reduced pressure and the residue was purified by a column chromatography (hexane/ethyl acetate, 10:1) affording compound **7** as a white solid (16.6 mg, 75%).^15b^

#### (*S*)-1′-Benzyl-3-phenyl-5*H*-spiro[furan-2,3′-indoline]-2′,5-dione (**7**)

[α]_D_^25^ = +15.0 (*c* 0.4, CHCl_3_, er 93:7). ^1^H NMR (500 MHz, CDCl_3_): δ 7.37–7.33 (m, 2H), 7.30–7.28 (m, 3H), 7.24–7.22 (m, 2H), 7.20–7.17 (m, 3H), 7-09-7.04 (m, 3H), 6.88 (d, *J* = 7.9 Hz, 1H), 6.67 (s, 1H), 5.13 (d, *J* = 15.4 Hz, 1H), 4.76 (d, *J* = 15.5 Hz, 1H). ^13^C {^1^H} NMR (126 MHz, CDCl_3_): δ 171.3, 170.0, 163.0, 143.5, 134.7, 131.8, 131.4, 129.1, 129.0, 128.8, 128.1, 127.6, 127.2, 125.1, 124.0, 123.8, 117.0, 110.4, 86.5, 44.6. HPLC (Chiralcel OD, *n*-hexane/2-propanol 85:15, λ = 254 nm, 0.4 mL/min). *t*_R_ (major) = 77.7 min, *t*_R_ (minor) = 87.8 min (er 93:7).

## Data Availability

The data underlying this study are available in the published article and its Supporting Information.

## Supporting Information Available

Copies of ^1^H, ^13^C, ^19^F NMR spectra, and HPLC profiles of all new compounds free of charge are available (PDF)
