# Supplementary material for: Access to Spiropyrazolone-butenolides through NHC-Catalyzed [3 + 2]-Asymmetric Annulation of 3-Bromoenals and 1H-Pyrazol-4,5-diones
Source: J Org Chem. 2023 May 11;88(11):6890–900. doi: 10.1021/acs.joc.3c00188 (PMC10242763; doi:10.1021/acs.joc.3c00188)
Supplement: Supplementary file 1 — jo3c00188_si_001.pdf [file jo3c00188_si_001.pdf]

Access to Spiropyrazolone-Butenolides through NHC-Catalyzed [3+2]-Asymmetric Annulation of 3-Bromoenals and 1*H*-Pyrazol-4,5-diones

Marta Gil-Ordóñez,[a] Alicia Maestro,[a]* and José M. Andrés[a]*

[a]GIR-SintACat-Instituto Universitario CINQUIMA y Departamento de Química Orgánica, Facultad de Ciencias, Universidad de Valladolid, Paseo Belén 7, 47011-Valladolid, Spain
E-mail: alicia.maestro@uva.es; jmandres@uva.es

Table of Contents

Copies of ^{1}H NMR, $^{13}C\{^{1}H\}$ NMR, ^{19}F NMR and HPLC profiles……………………………………………….. S2

Copies of ^{1}H NMR, $^{13}C\{^{1}H\}$ NMR and HPLC profiles of 3a

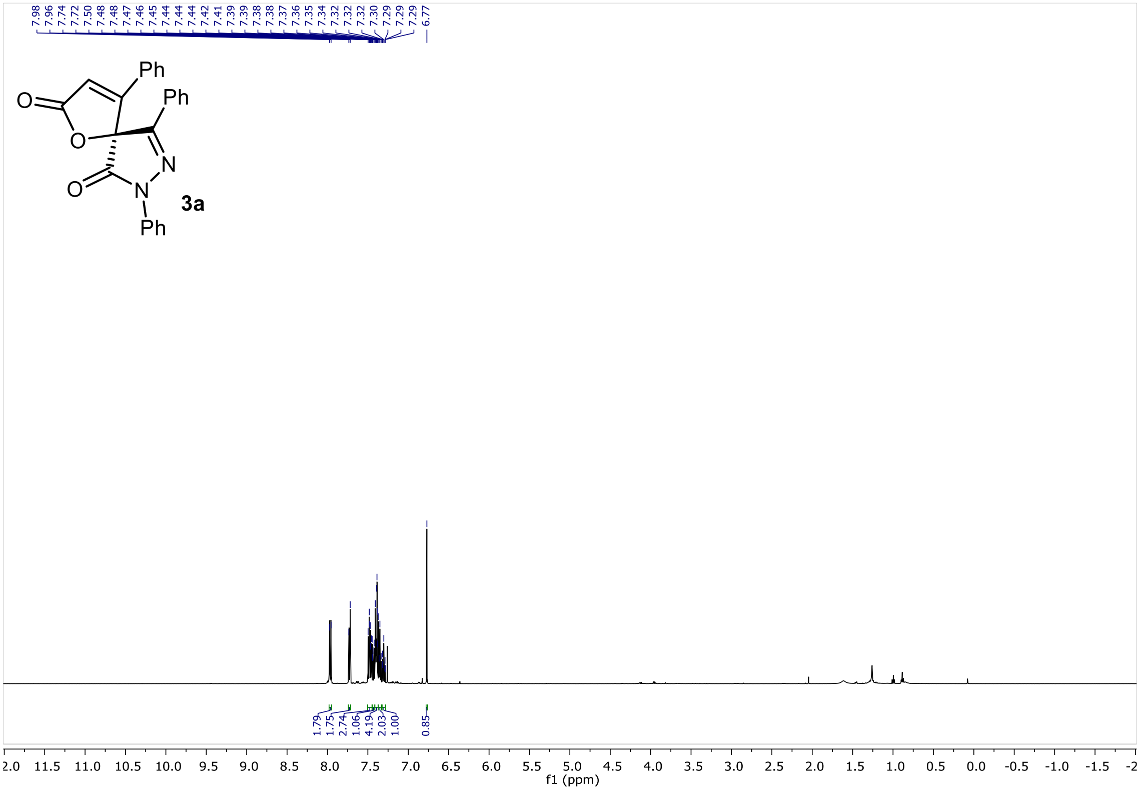

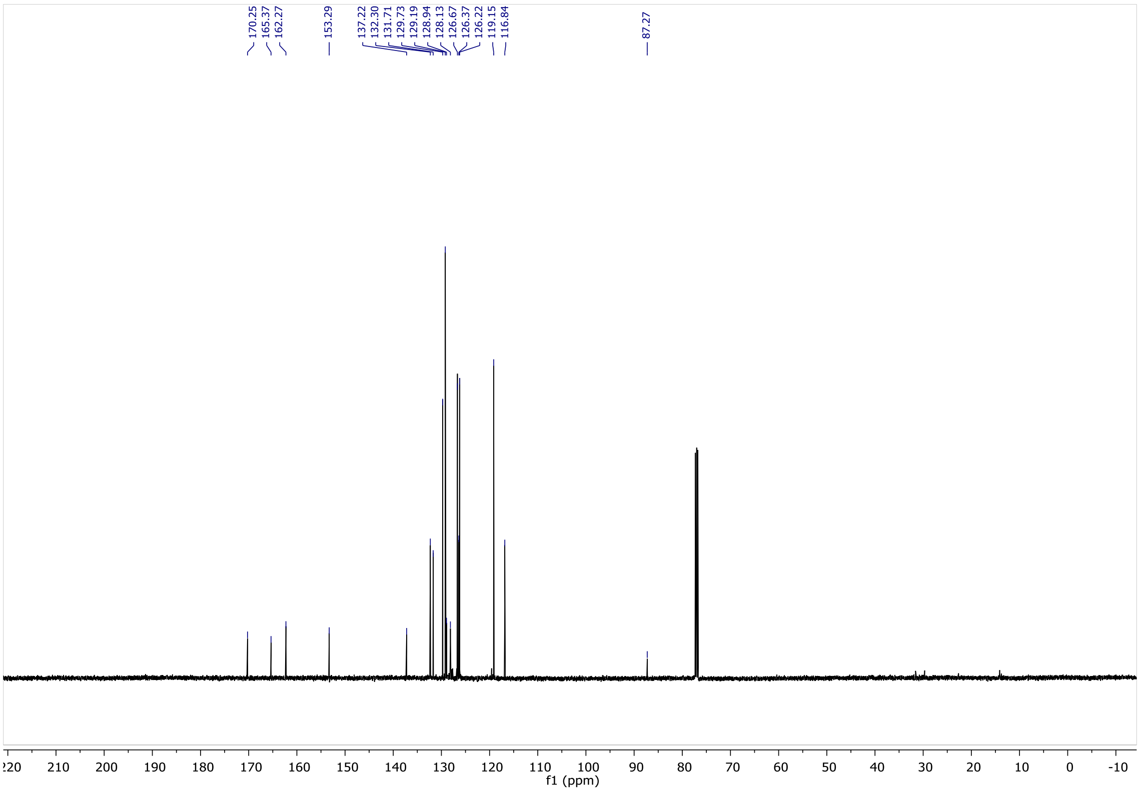

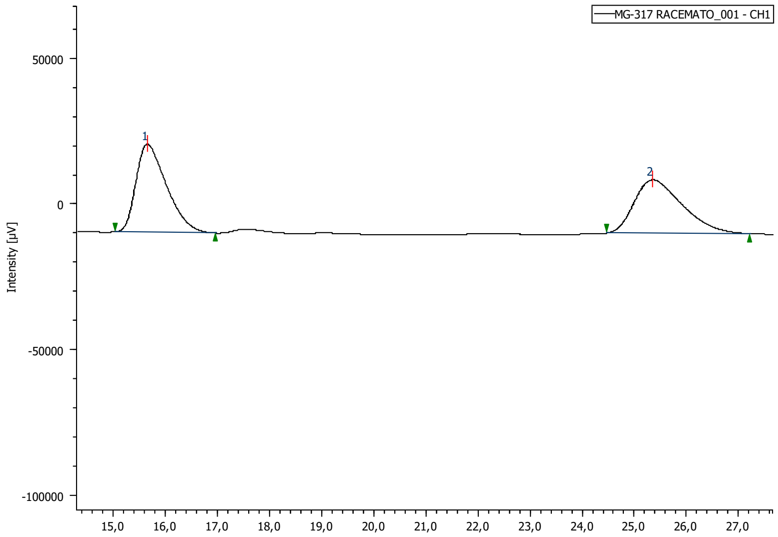

	Time	Area	Area%
1	15,650	1231287	**51,245**
2	25,342	1171439	**48,755**

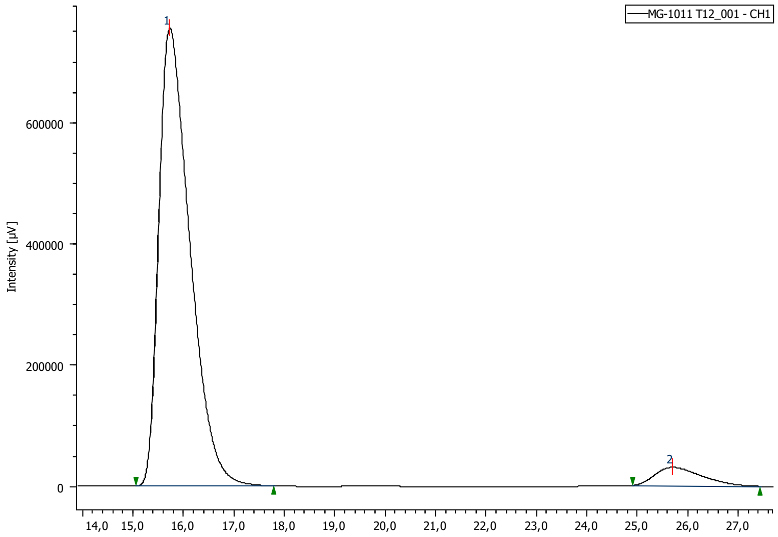

	Time	Area	Area%
1	15,725	32226586	**94,247**
2	25,683	1967089	**5,753**

Copies of ^{1}H NMR, $^{13}C\{^{1}H\}$ NMR and HPLC profiles of 3b

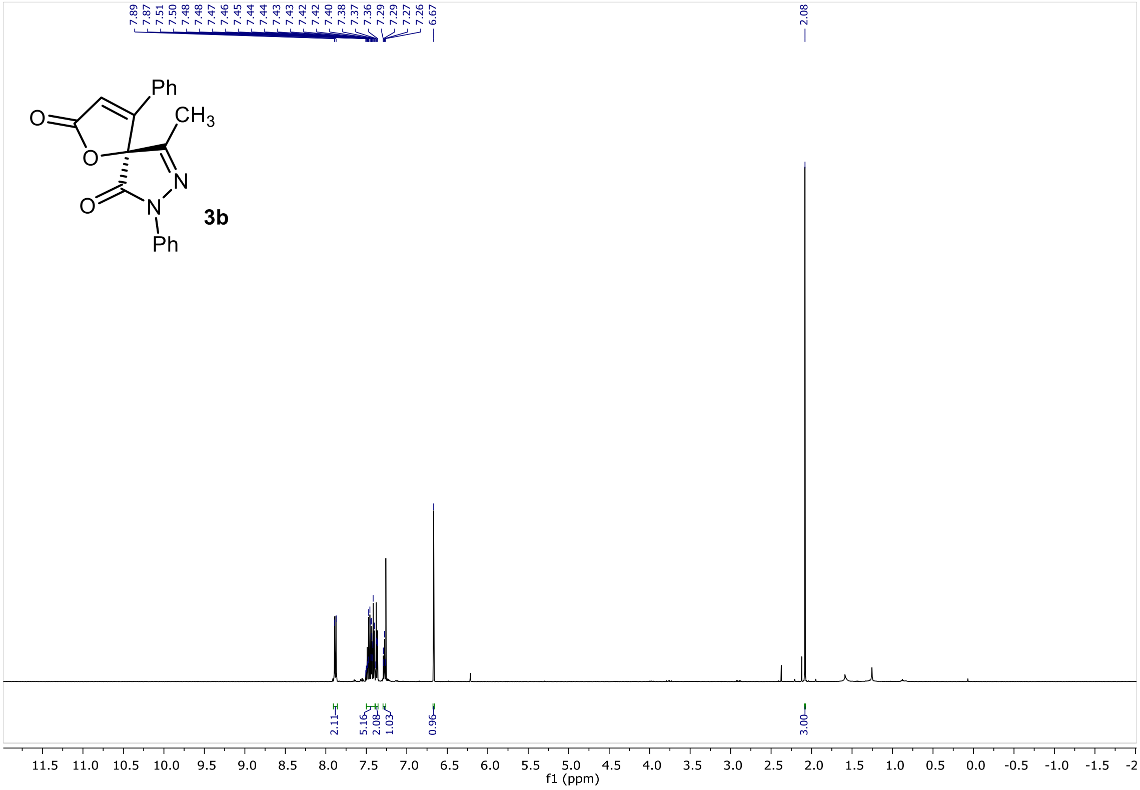

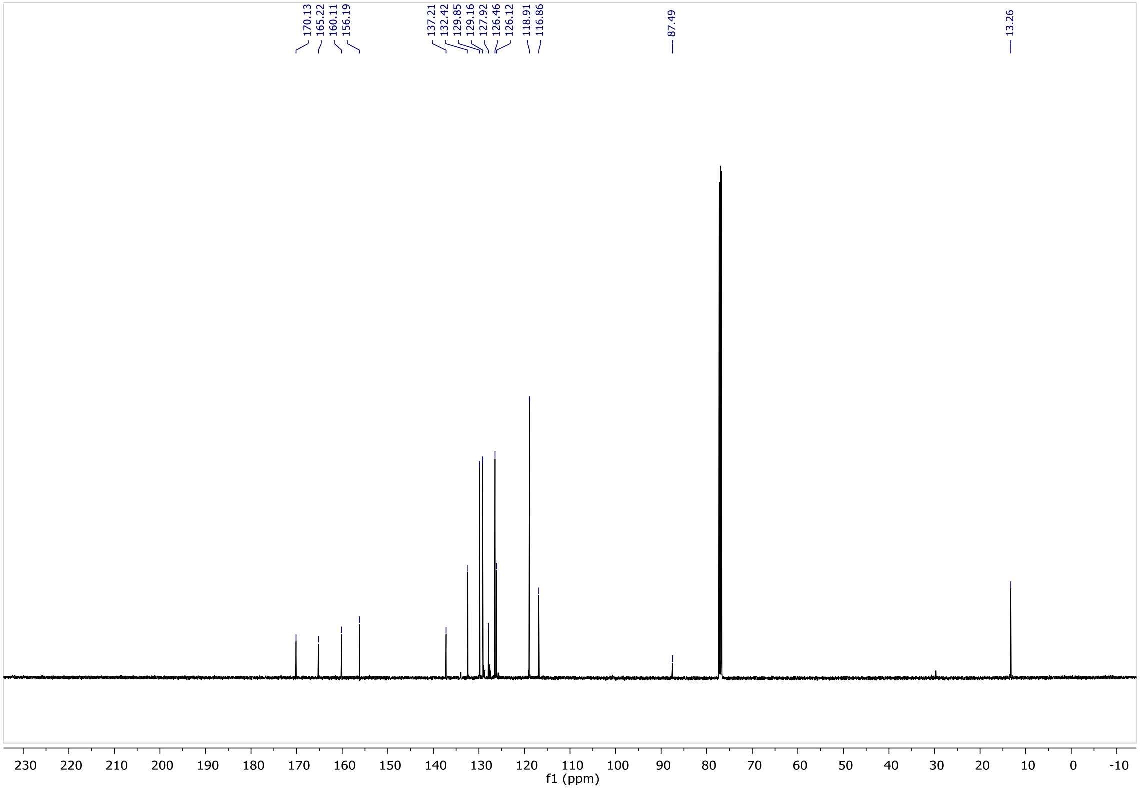

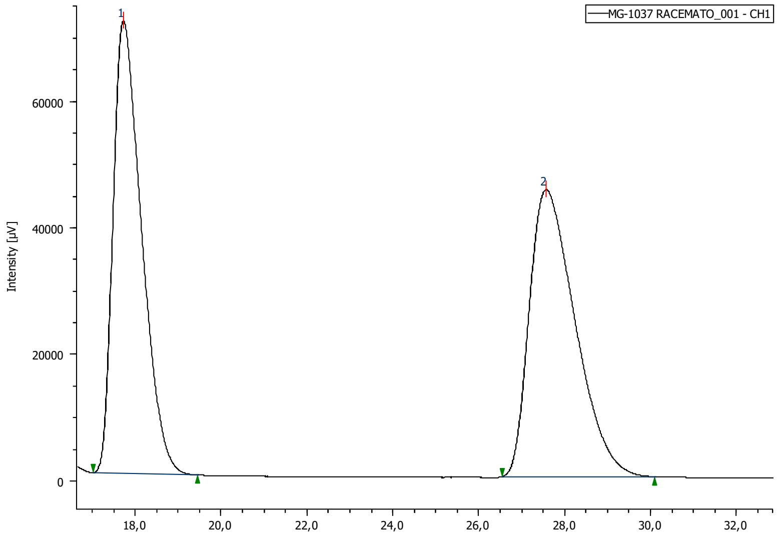

	Time	Area	Area%
1	17,733	3287551	**49,536**
2	27,558	3349153	**50,464**

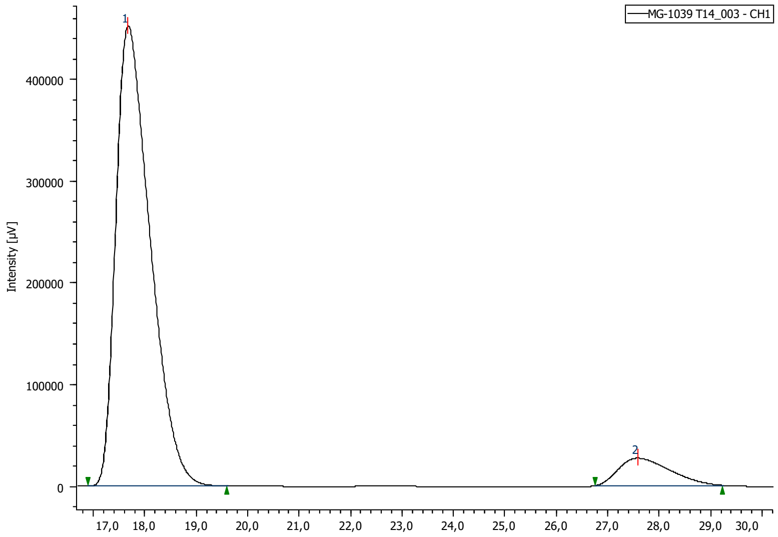

	Time	Area	Area%
1	17,675	20906390	**91,813**
2	27,567	1864217	**8,187**

Copies of ^{1}H NMR, ^{13}C{^{1}H} NMR and HPLC profiles of 3c

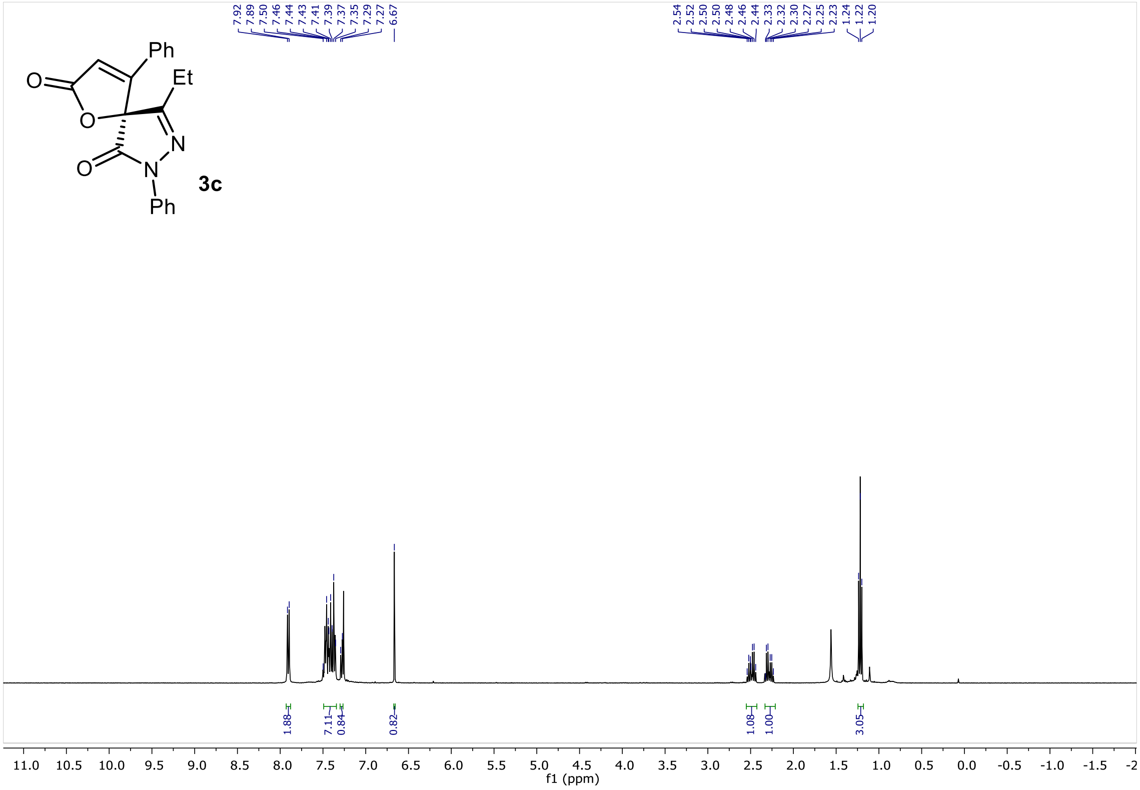

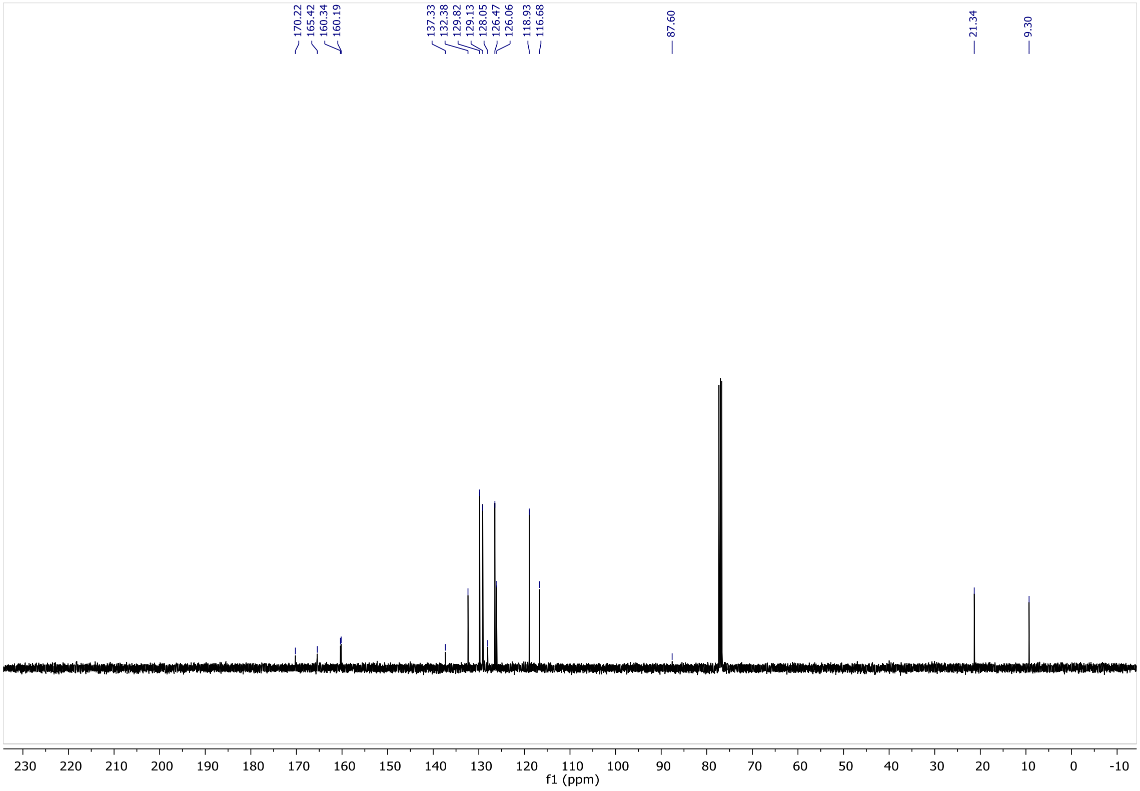

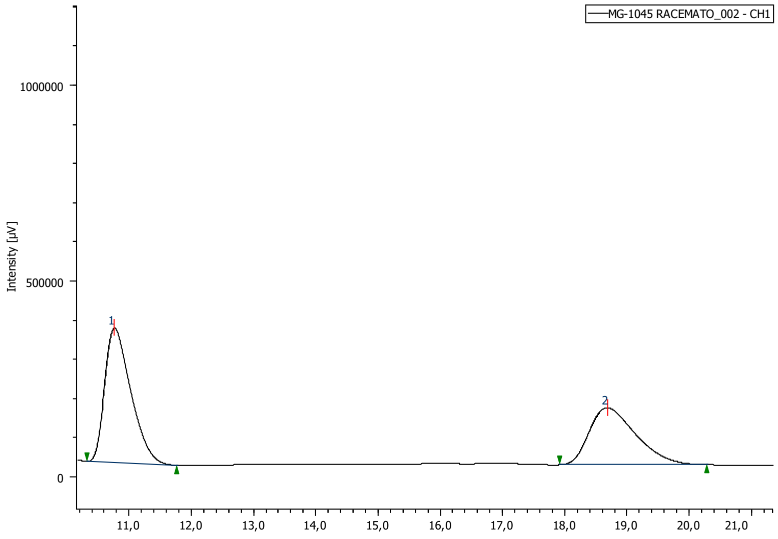

	Time	Area	Area%
1	10,767	10009865	**57,156**
2	18,675	7503219	**42,844**

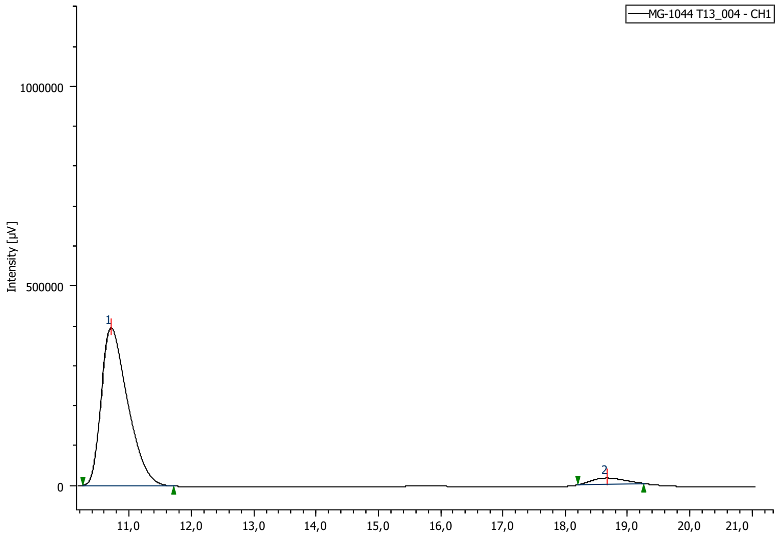

	Time	Area	Area%
1	10,717	11682171	**95,303**
2	18,658	575755	**4,697**

Copies of ^{1}H NMR, $^{13}C\{^{1}H\}$ NMR and HPLC profiles of 3d

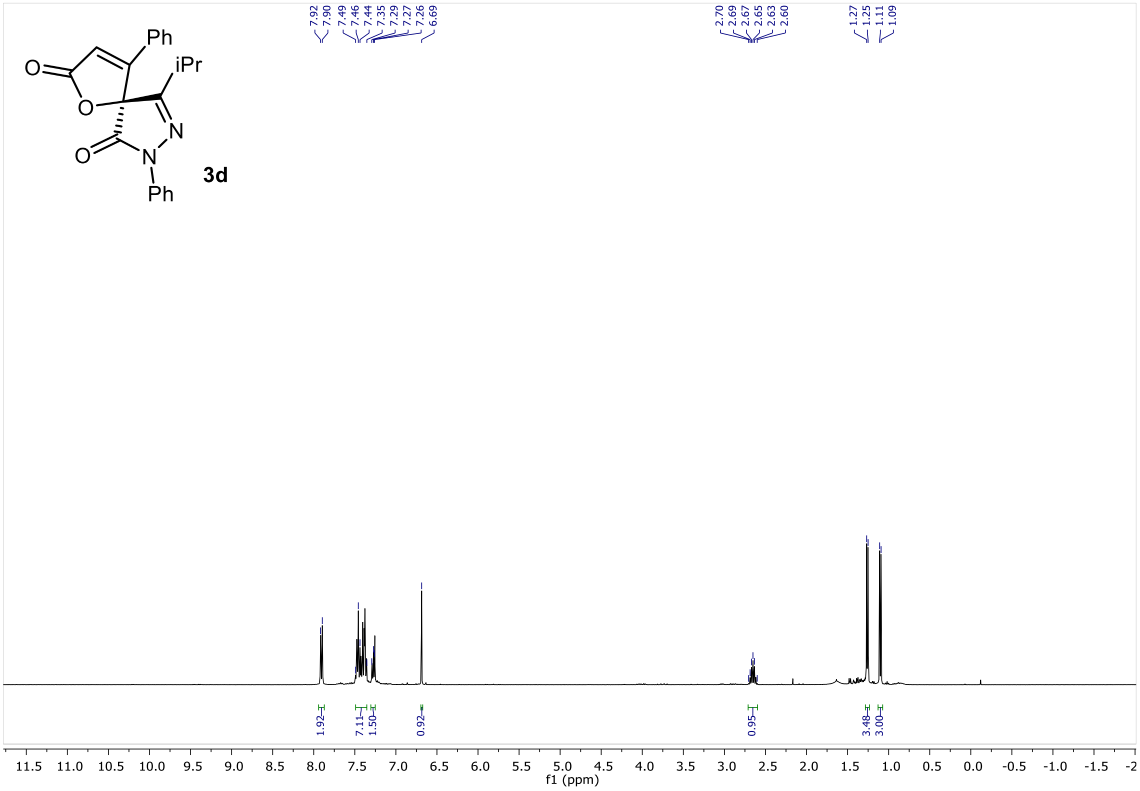

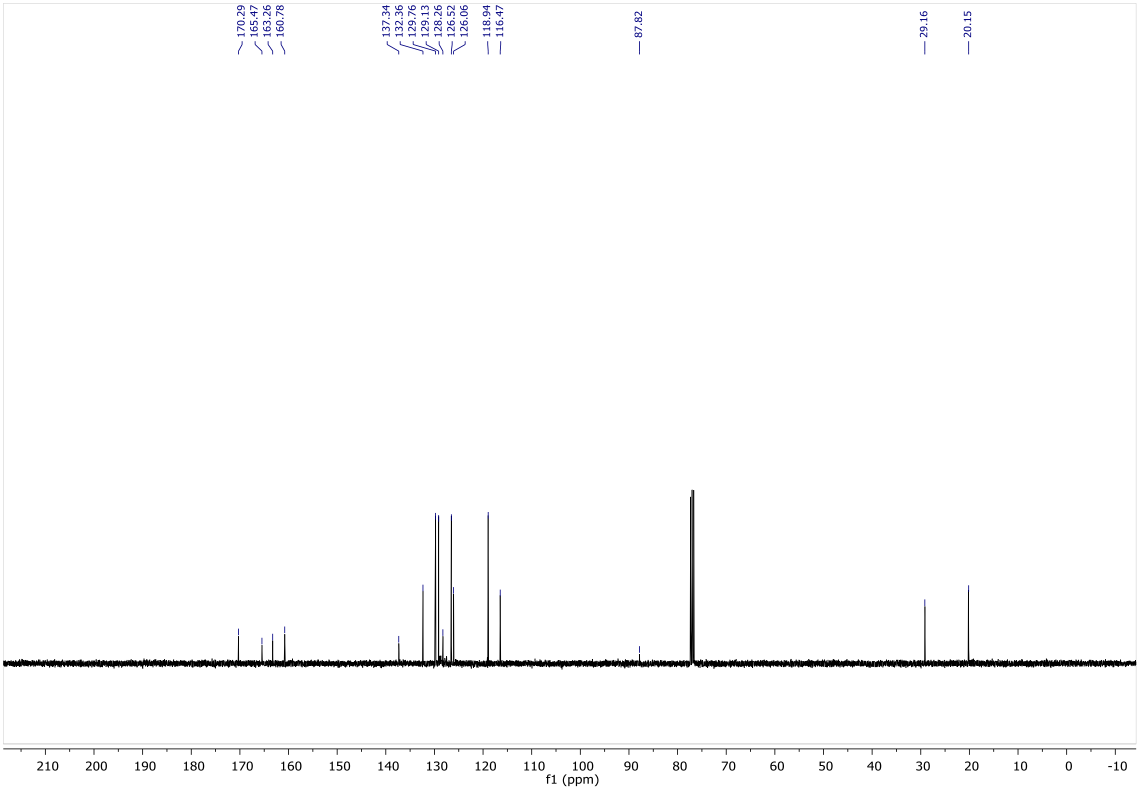

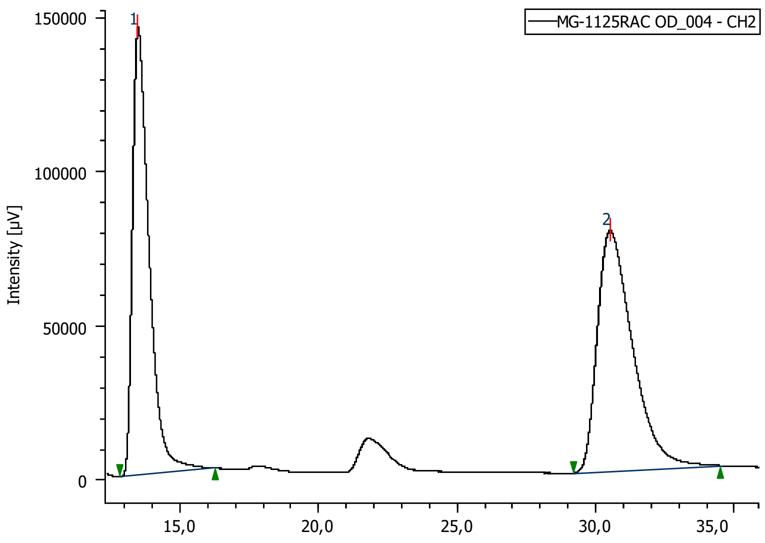

	Time	Area	Area%
1	13,493	6032370	**46,639**
2	30,490	6901937	**53,361**

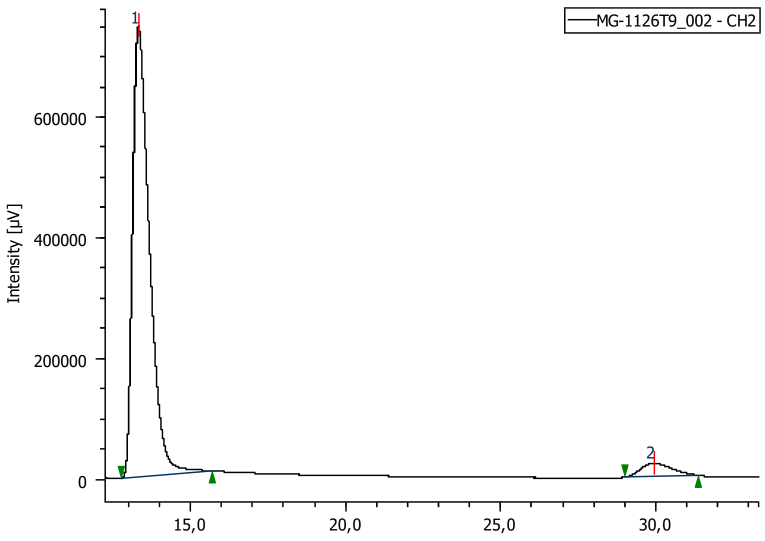

	Time	Area	Area%
1	13,335	28025847	**94,820**
2	29,893	1530975	**5,180**

Copies of ^{1}H NMR, $^{13}C\{^{1}H\}$ NMR and HPLC profiles of 3e

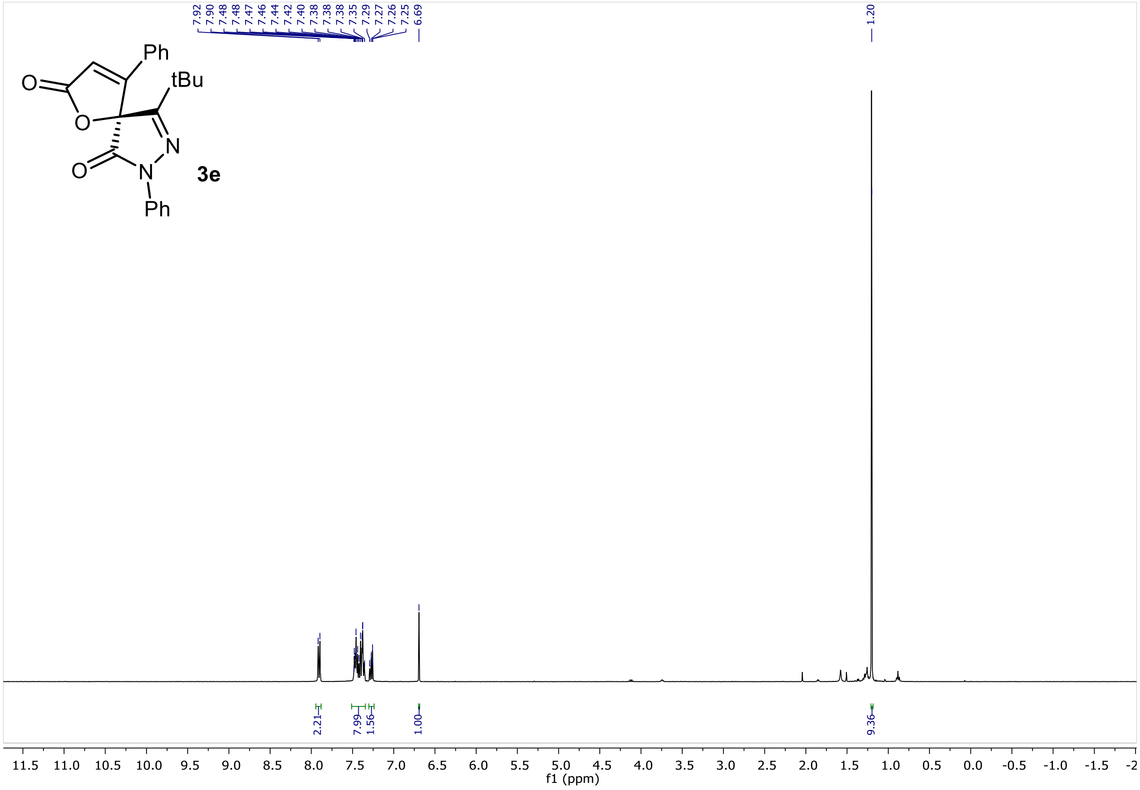

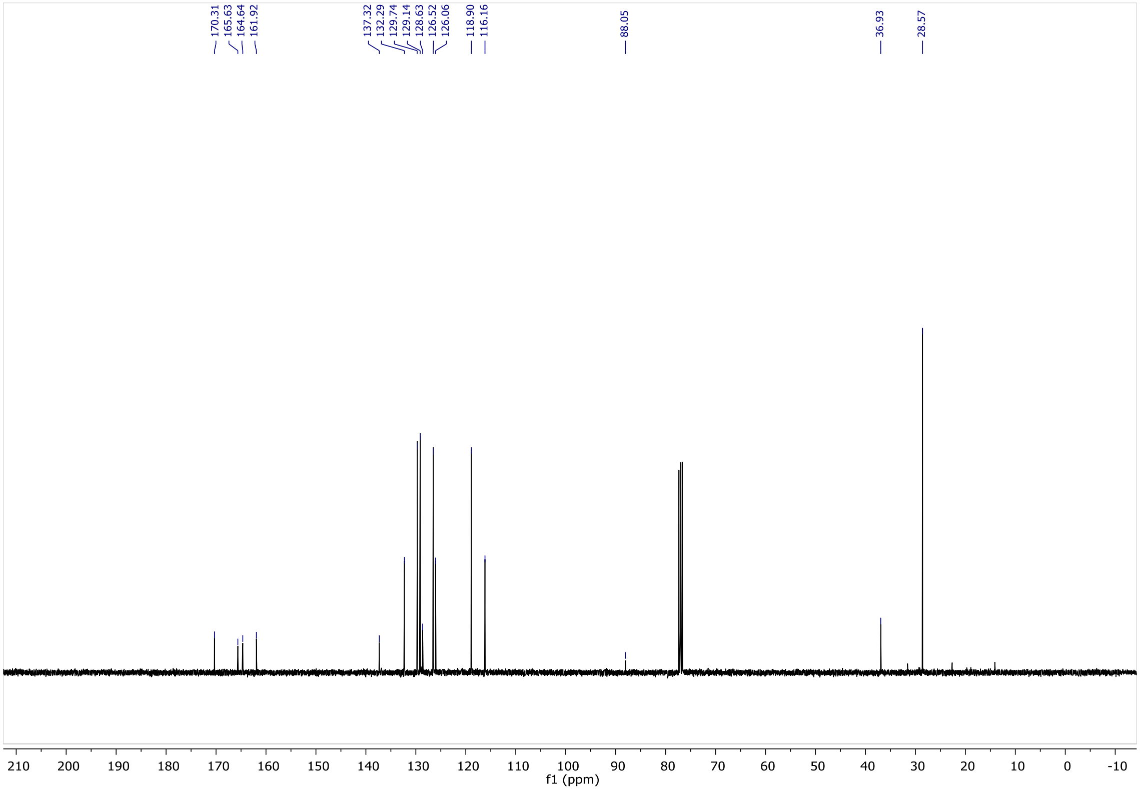

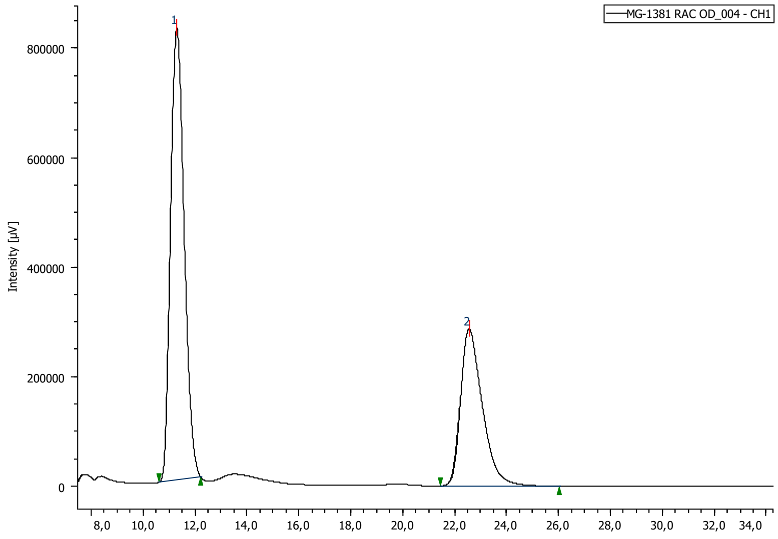

	t_R	Area	Area%
1	11,300	29832540	**64,200**
2	22,542	16635243	**35,800**

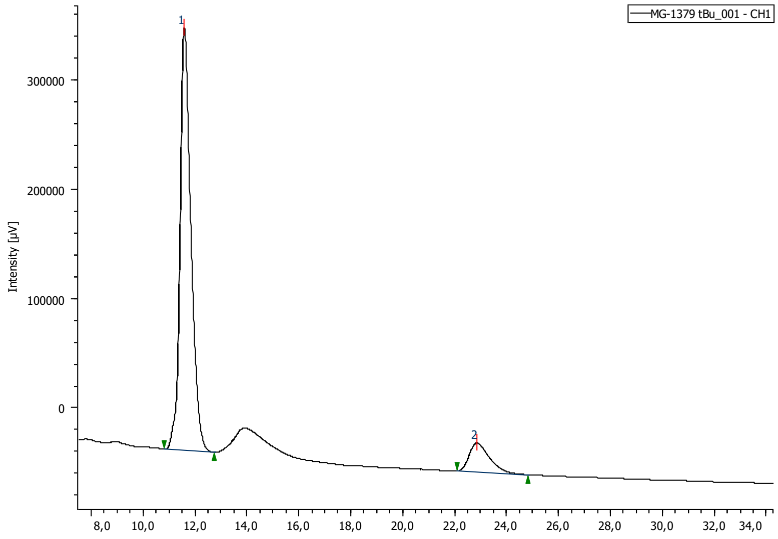

	t_R	Area	Area%
1	11,592	11252323	**88,649**
2	22,850	1440798	**11,351**

Copies of ^{1}H NMR, ^{13}C{^{1}H} NMR and HPLC profiles of 3f

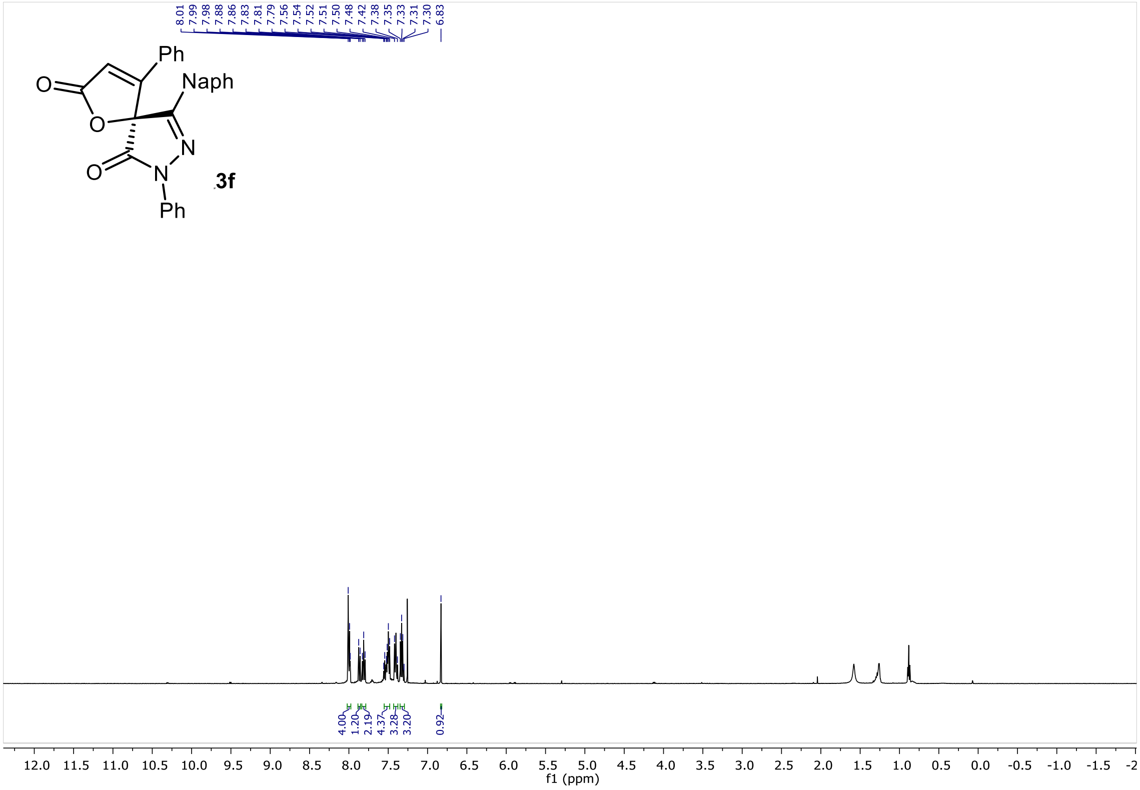

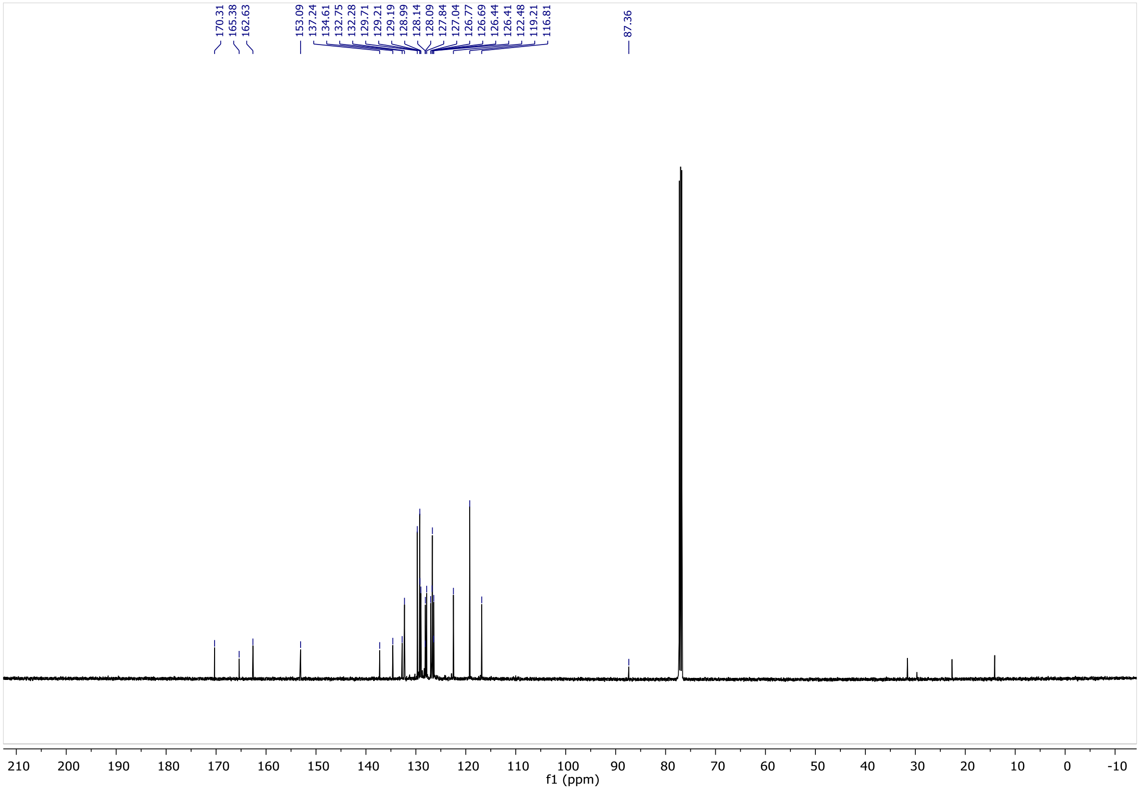

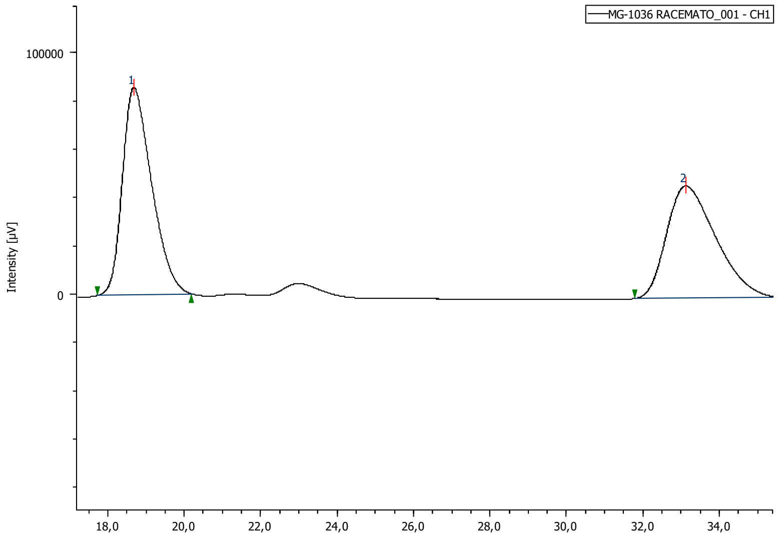

	Time	Area	Area%
1	18,683	4551435	**52,417**
2	33,108	4131772	**47,583**

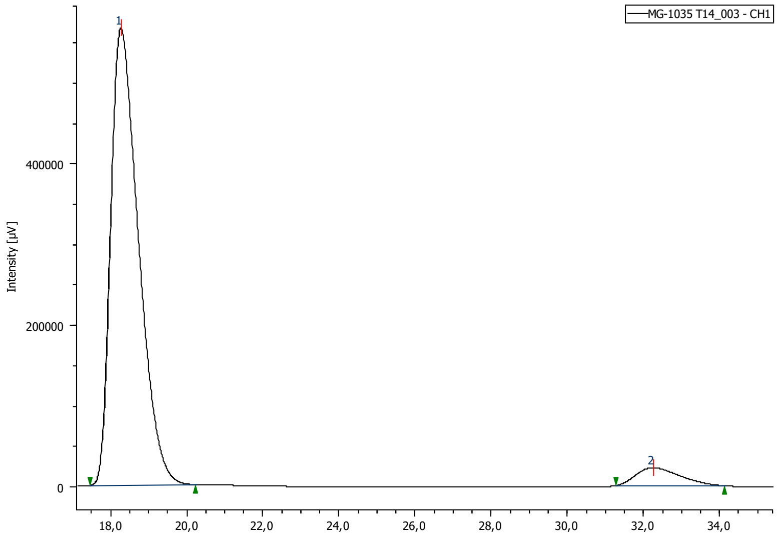

	Time	Area	Area%
1	18,275	28606733	**94,142**
2	32,233	1780061	**5,858**

Copies of ^{1}H NMR, ^{13}C{^{1}H} NMR and HPLC profiles of 3g

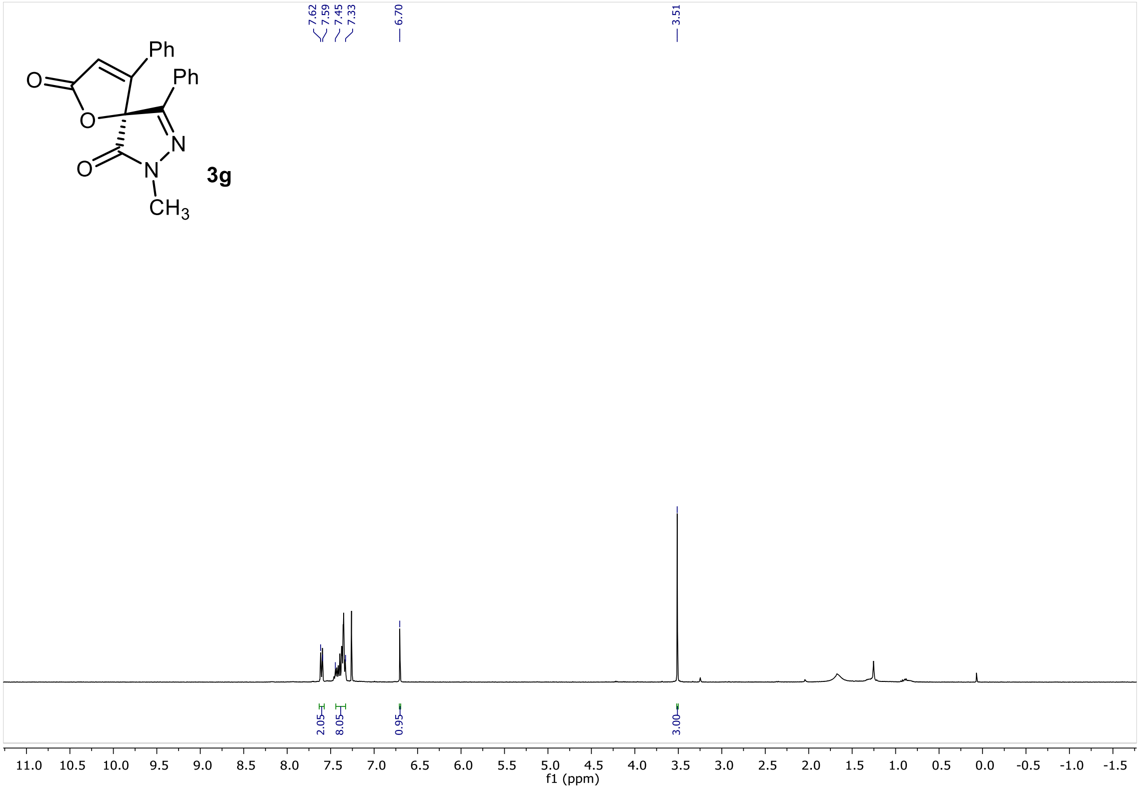

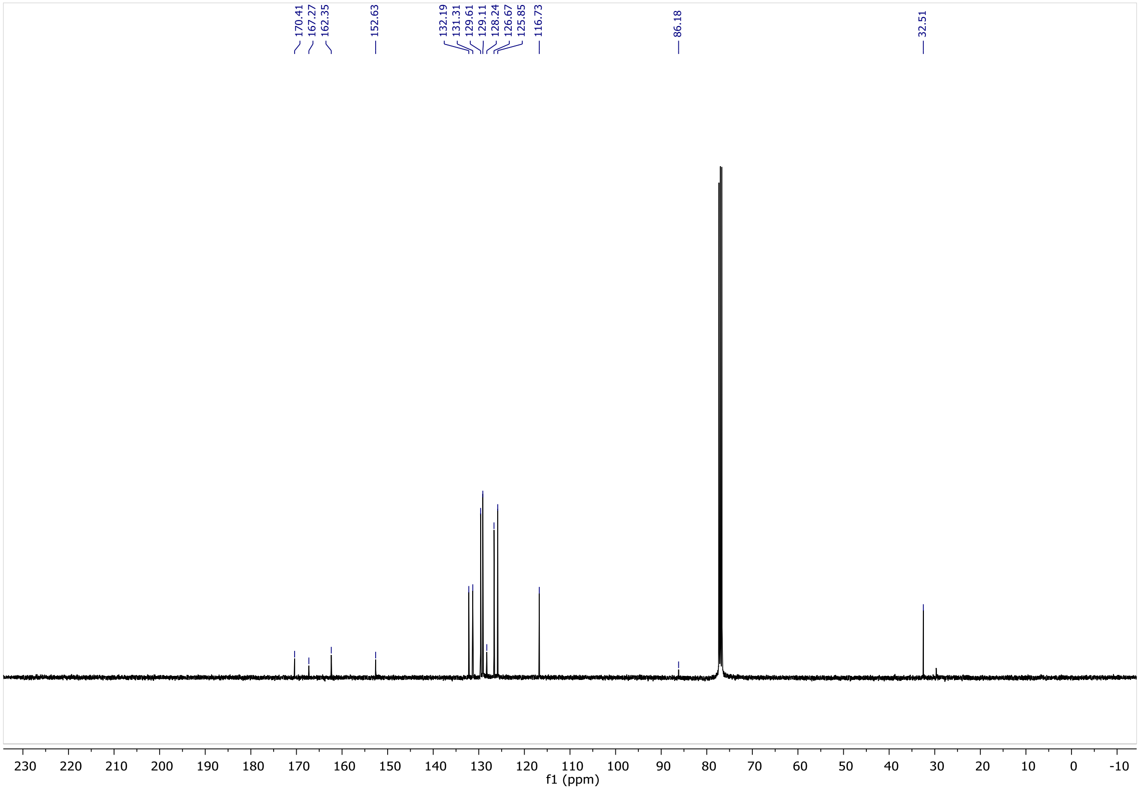

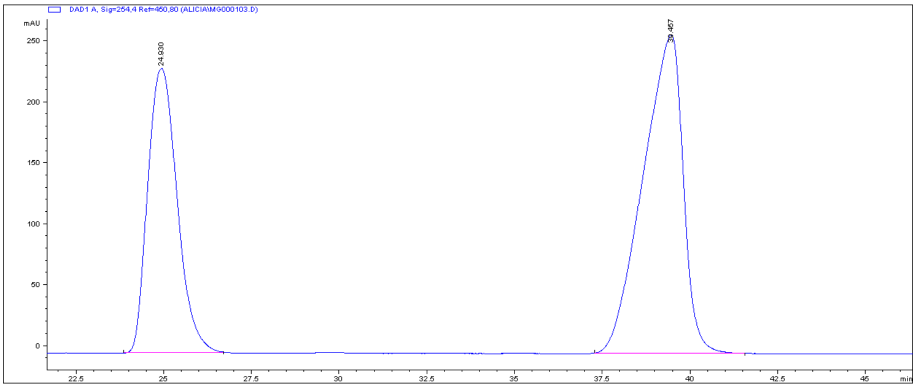

	Time	Area	Area%
1	24.93	13617.1	**39.588**
2	39.457	20779.9	**60.412**

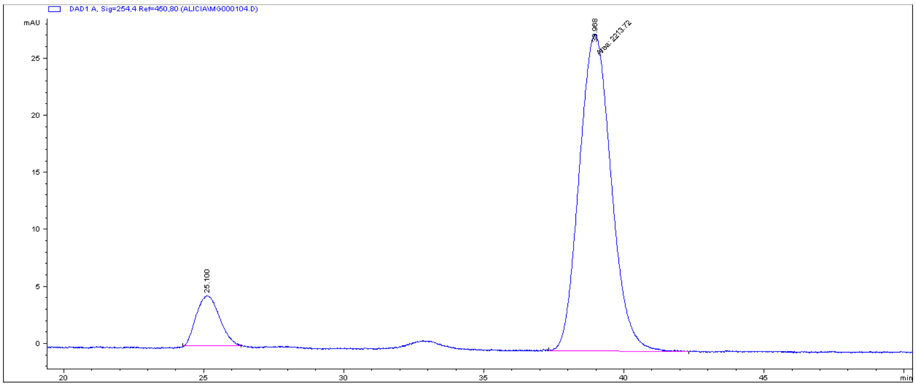

	Time	Area	Area%
1	25.1	259.1	**10.479**
2	38.968	2213.7	**89.521**

Copies of ^1H NMR, $^{13}C\{^1H\}$ NMR and HPLC profiles of 3h

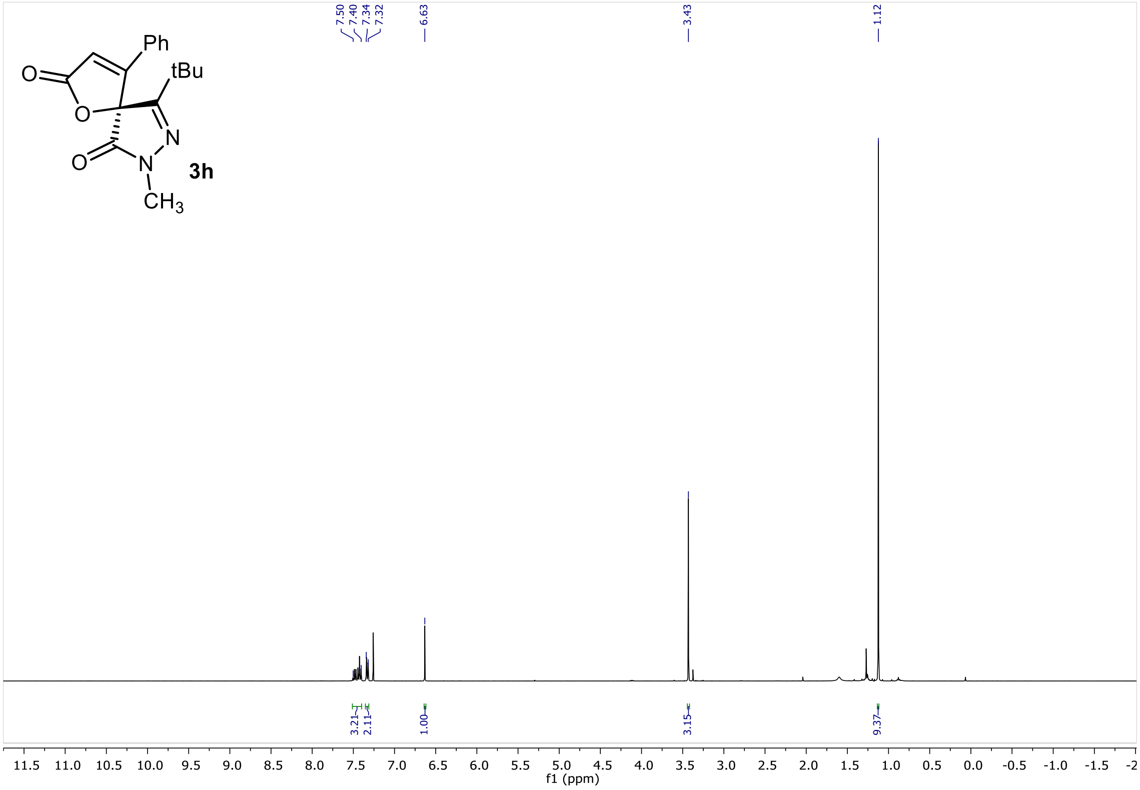

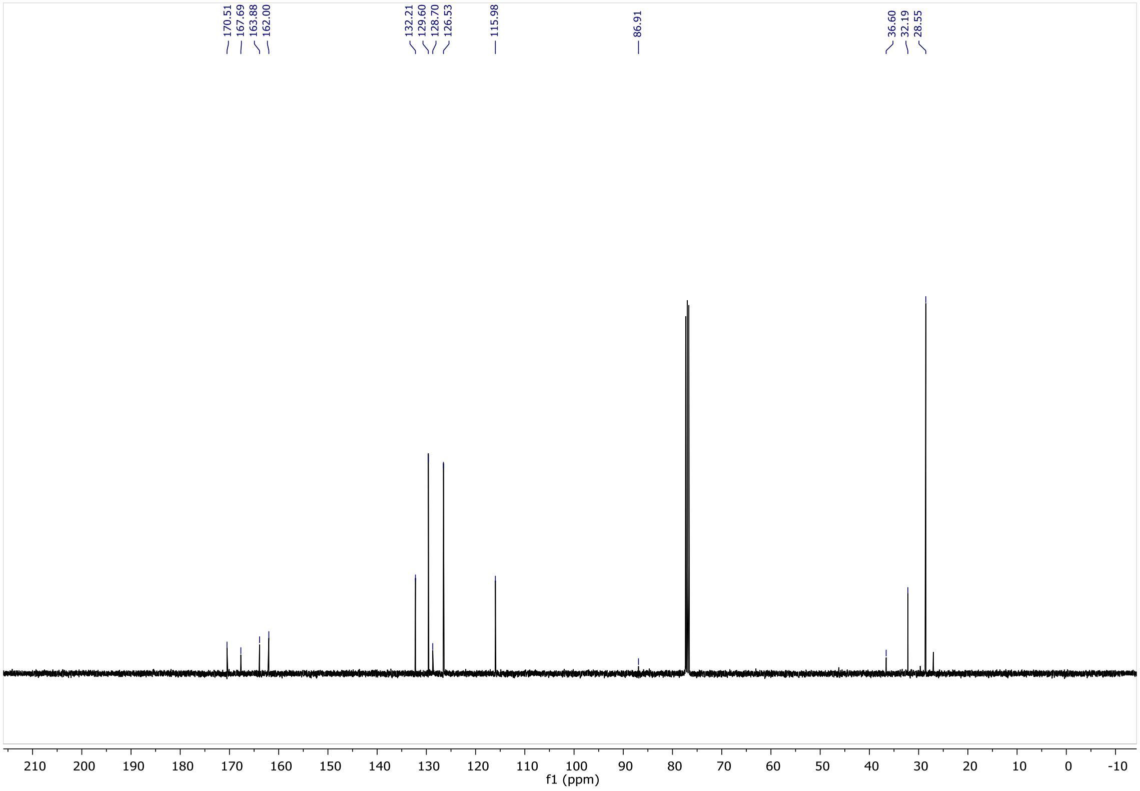

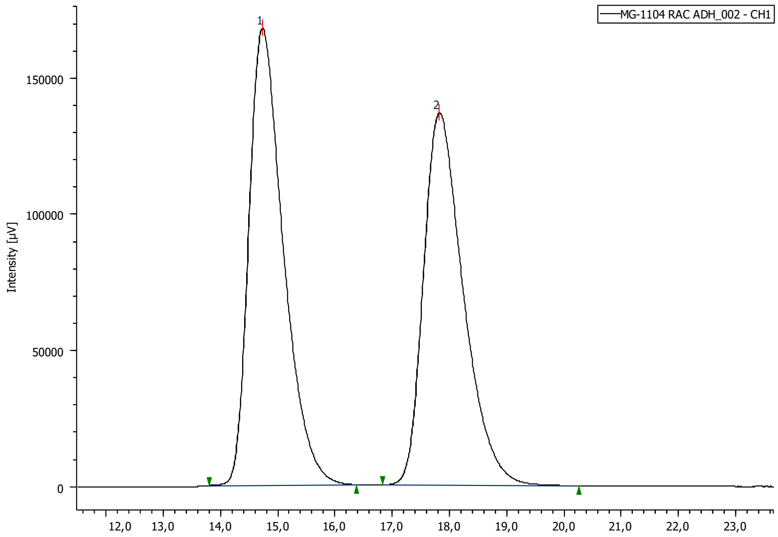

	Time	Area	Area%
1	14,733	6990526	**51,422**
2	17,817	6603954	**48,578**

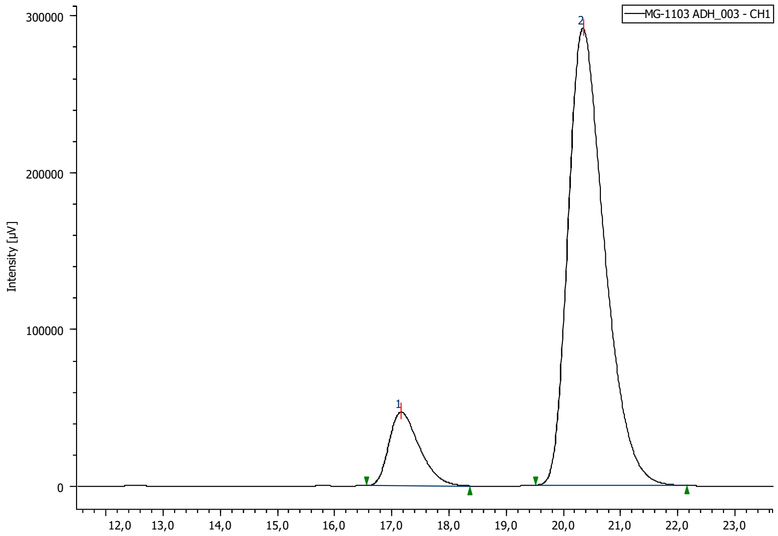

	Time	Area	Area%
1	17,158	1729804	**11,851**
2	20,333	12866053	**88,149**

Copies of HPLC profiles of 3i

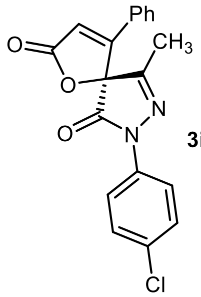

3i

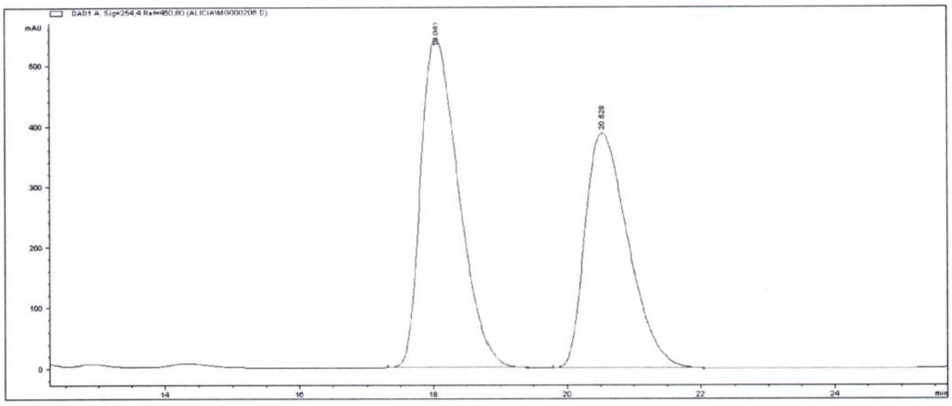

	Time	Area	Area%
1	18.041	21418.6	**55.243**
2	20.528	17353	**44.757**

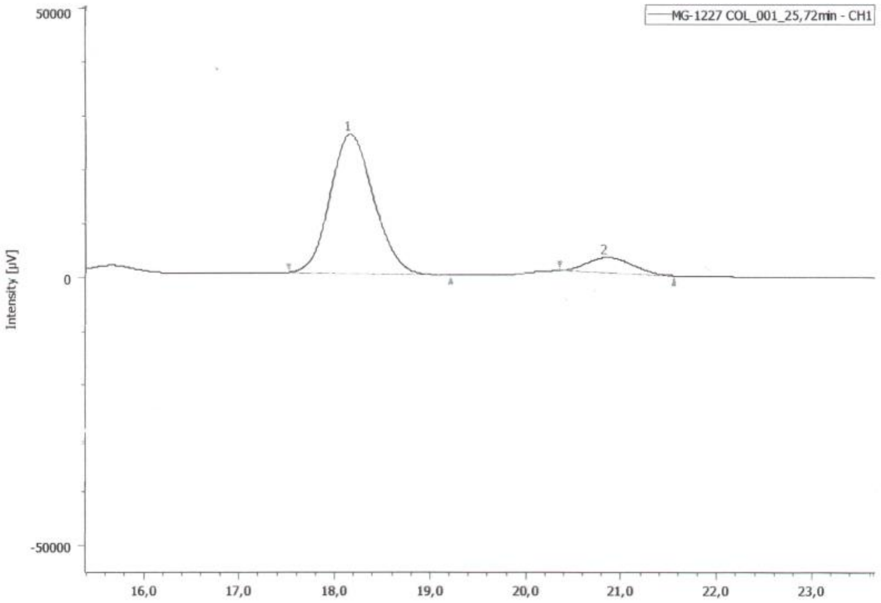

	Time	Area	Area%
1	18.158	811285	**89.721**
2	20.842	92944	**10.279**

Copies of ^{1}H NMR, ^{13}C{^{1}H} NMR and HPLC profiles of 3ab

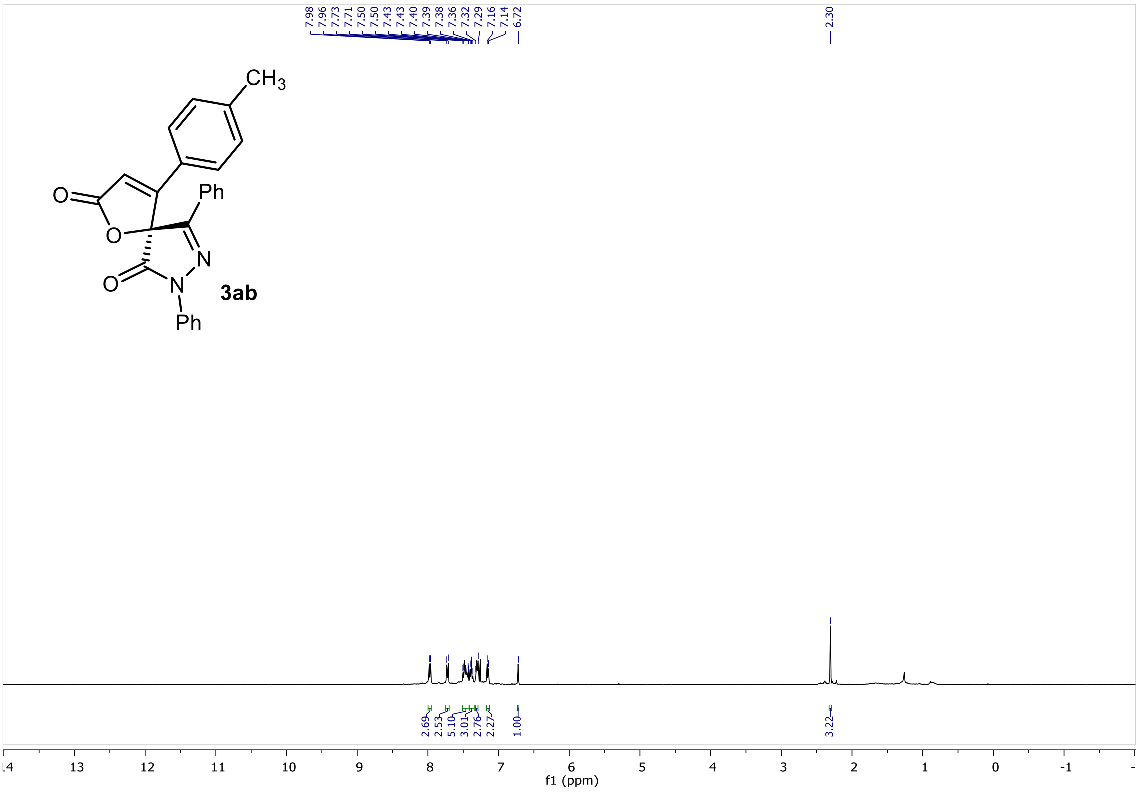

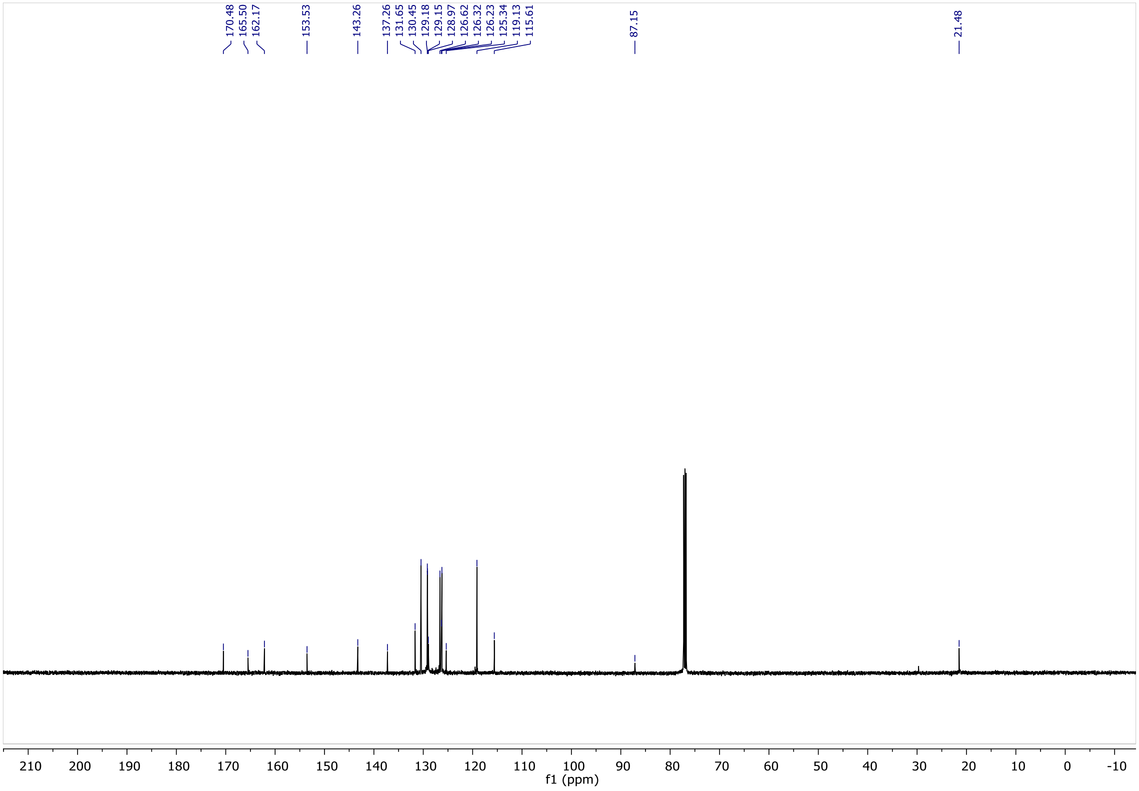

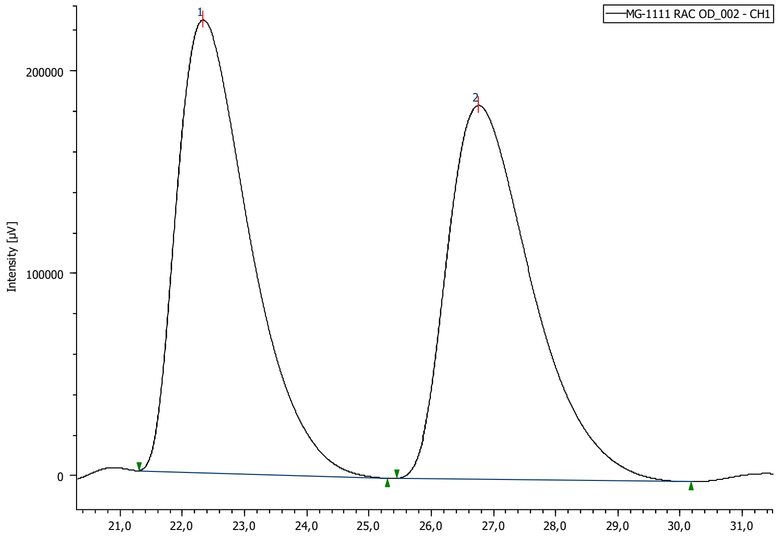

	t_R	Area	Area%
1	22,325	18267109	**51,107**
2	26,750	17475601	**48,893**

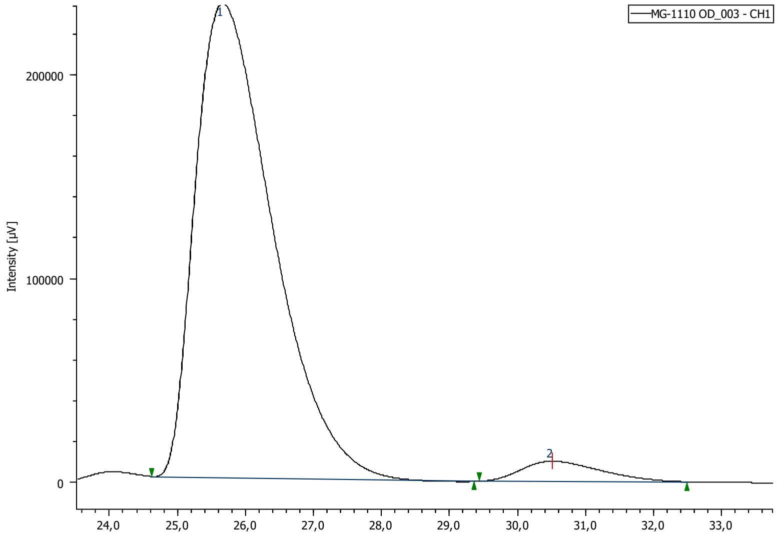

	t_R	Area	Area%
1	25,667	18365420	**95,801**
2	30,508	804898	**4,199**

Copies of ^{1}H NMR, $^{13}C\{^{1}H\}$ NMR and HPLC profiles of 3ac

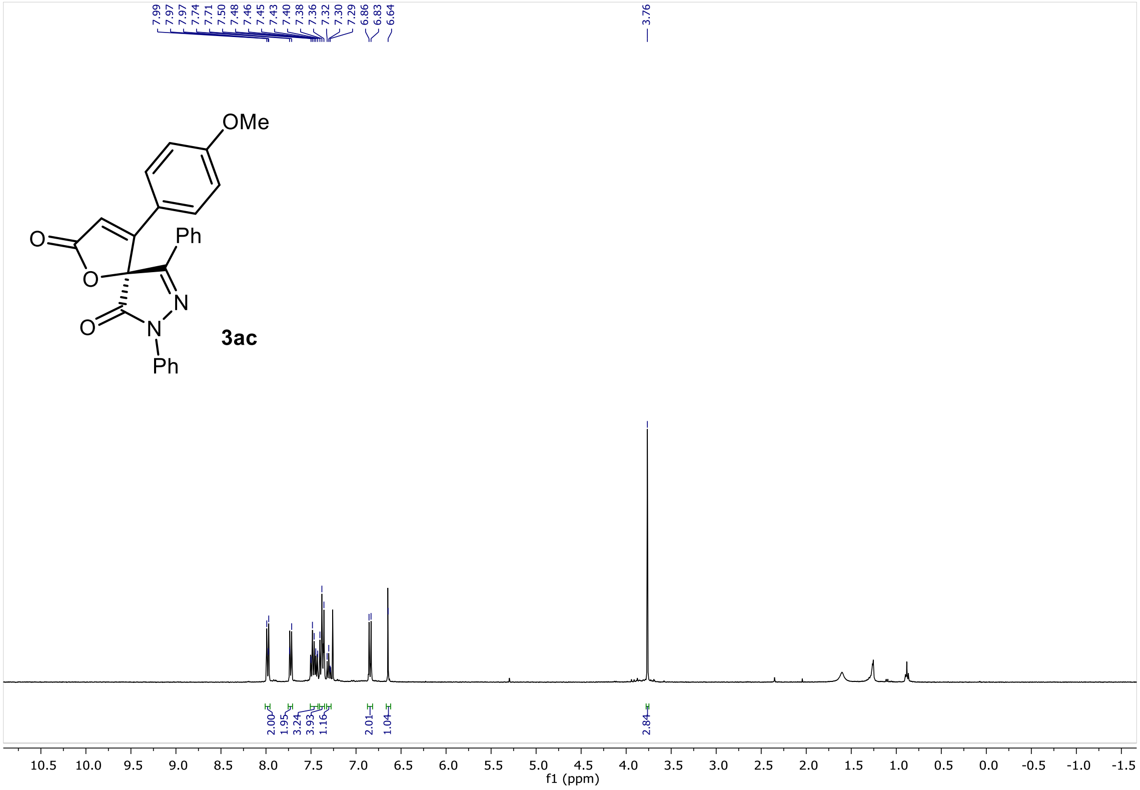

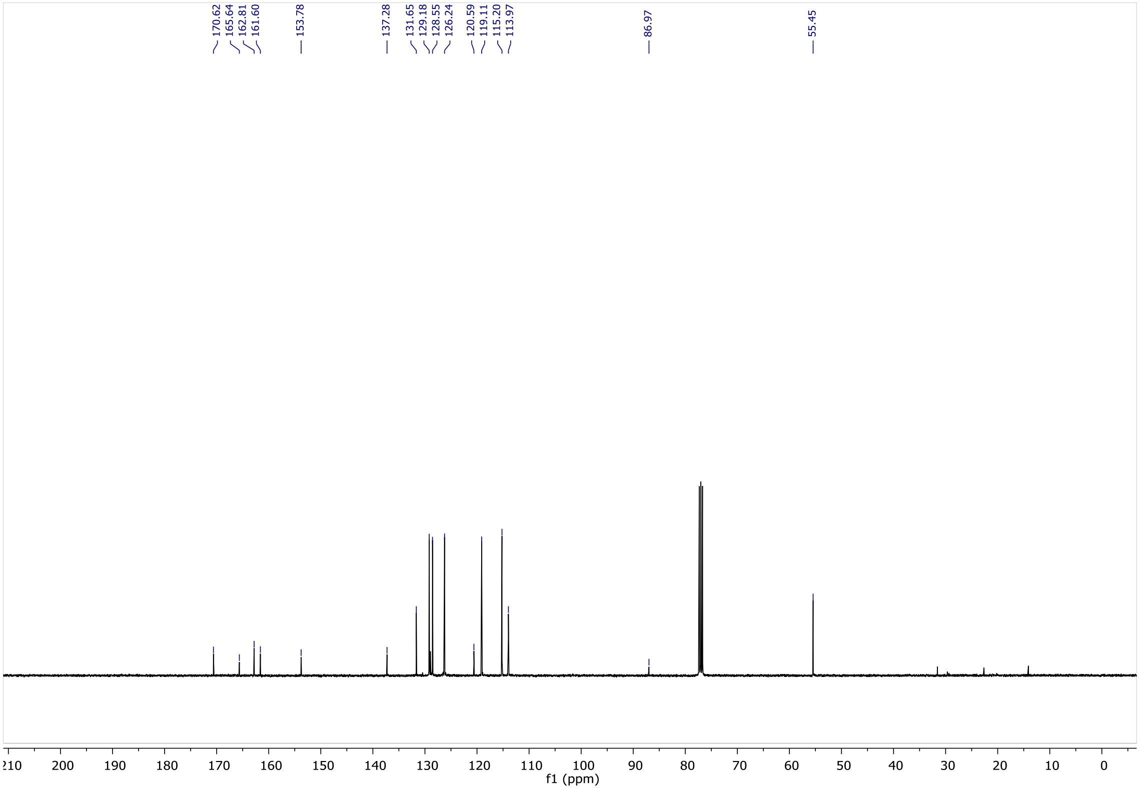

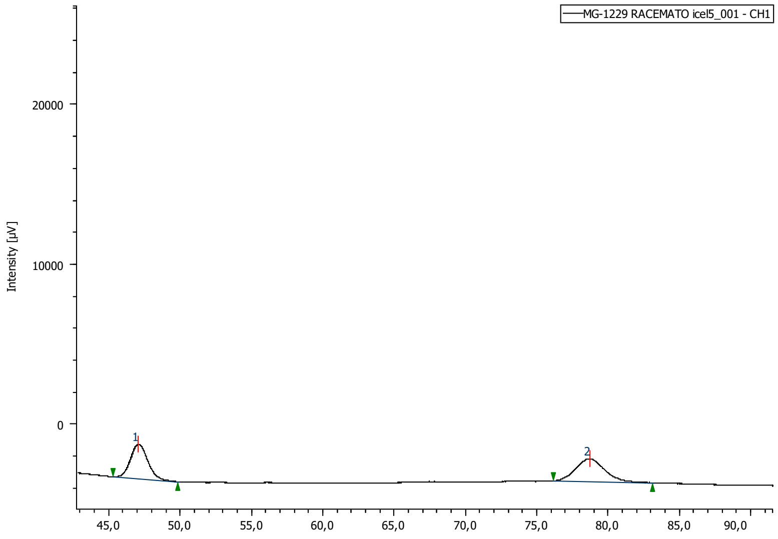

	Time	Area	Area%
1	47,075	182663	**47,733**
2	78,650	200013	**52,267**

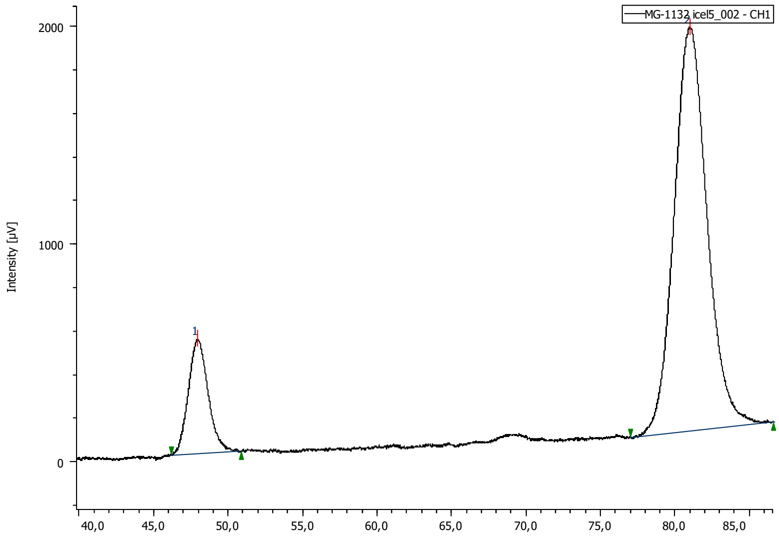

	Time	Area	Area%
1	47,942	47895	**14,402**
2	80,975	284675	**85,598**

Copies of ^{1}H NMR, ^{13}C{^{1}H} NMR and HPLC profiles of 3ad

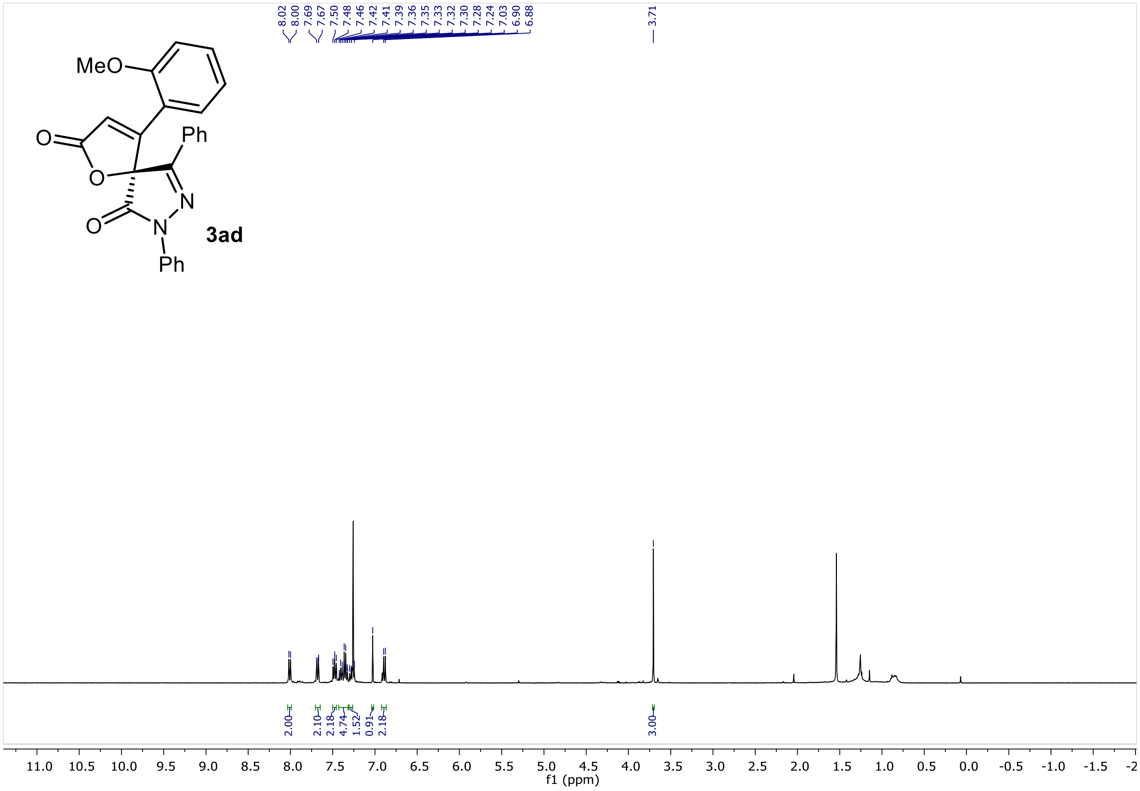

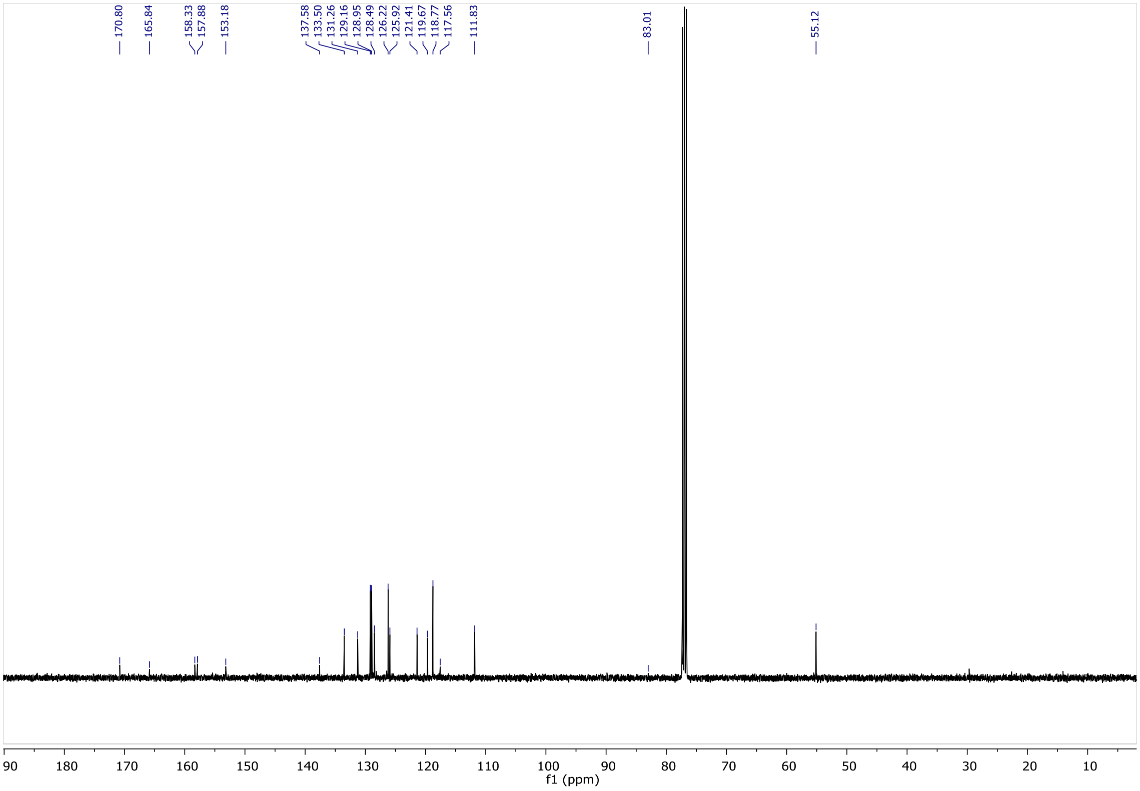

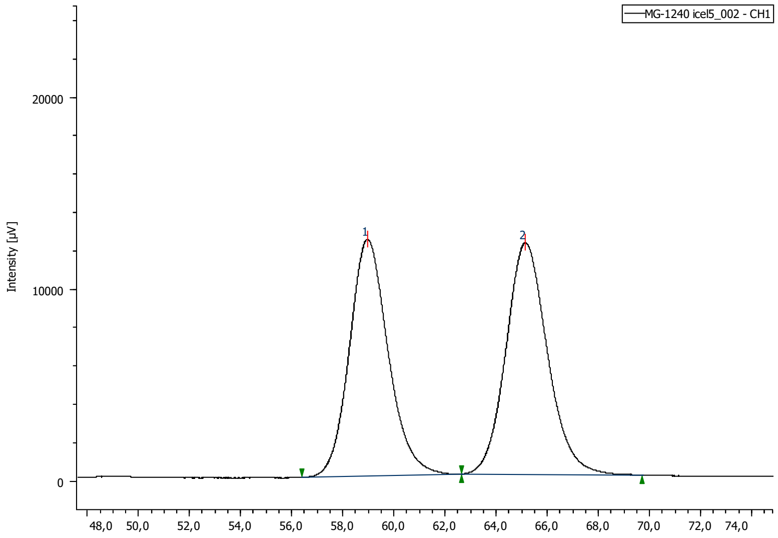

	Time	Area	Area%
1	58,950	1295555	**48,798**
2	65,117	1359361	**51,202**

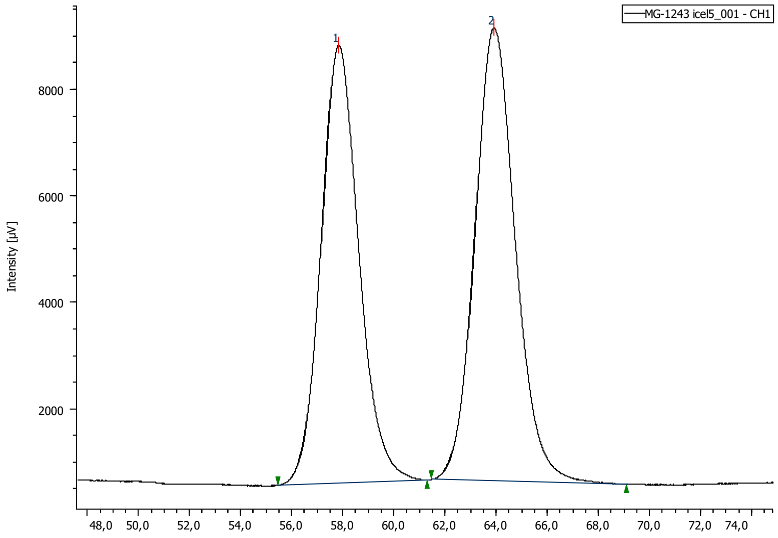

	Time	Area	Area%
1	57,817	844891	**47,392**
2	63,883	937893	**52,608**

Copies of ^{1}H NMR, ^{13}C{^{1}H} NMR, ^{19}F NMR and HPLC profiles of 3ae

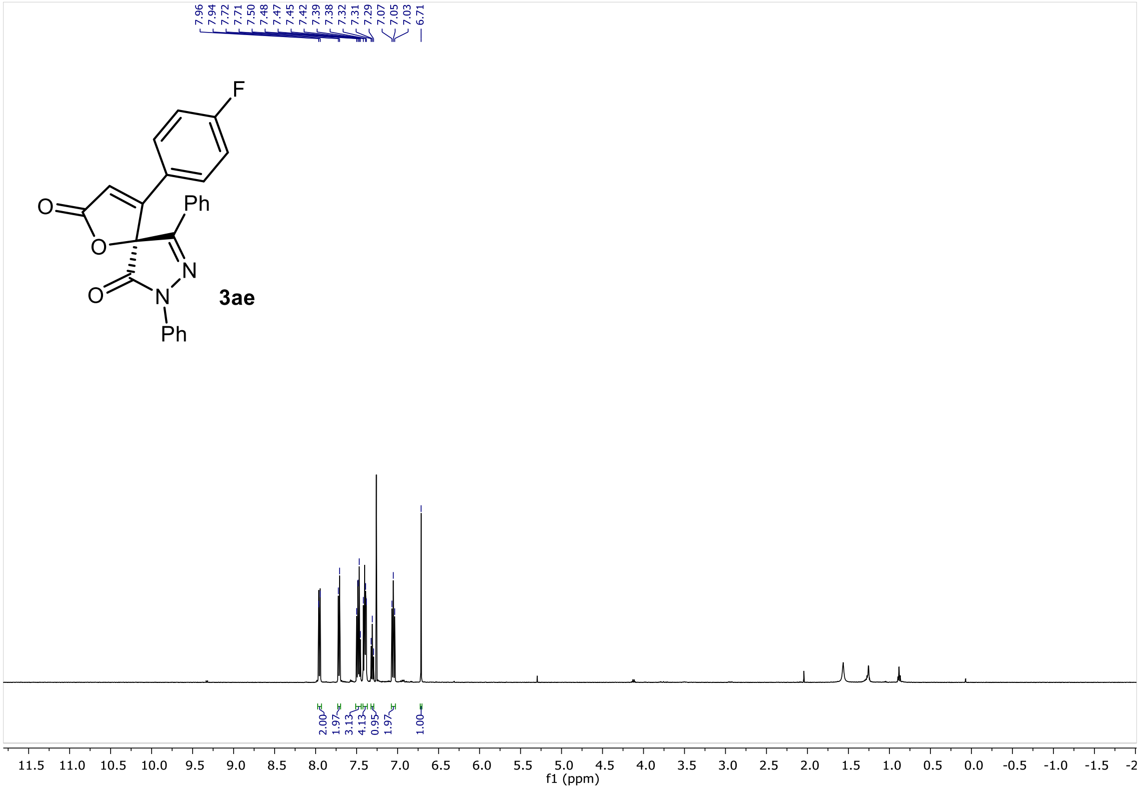

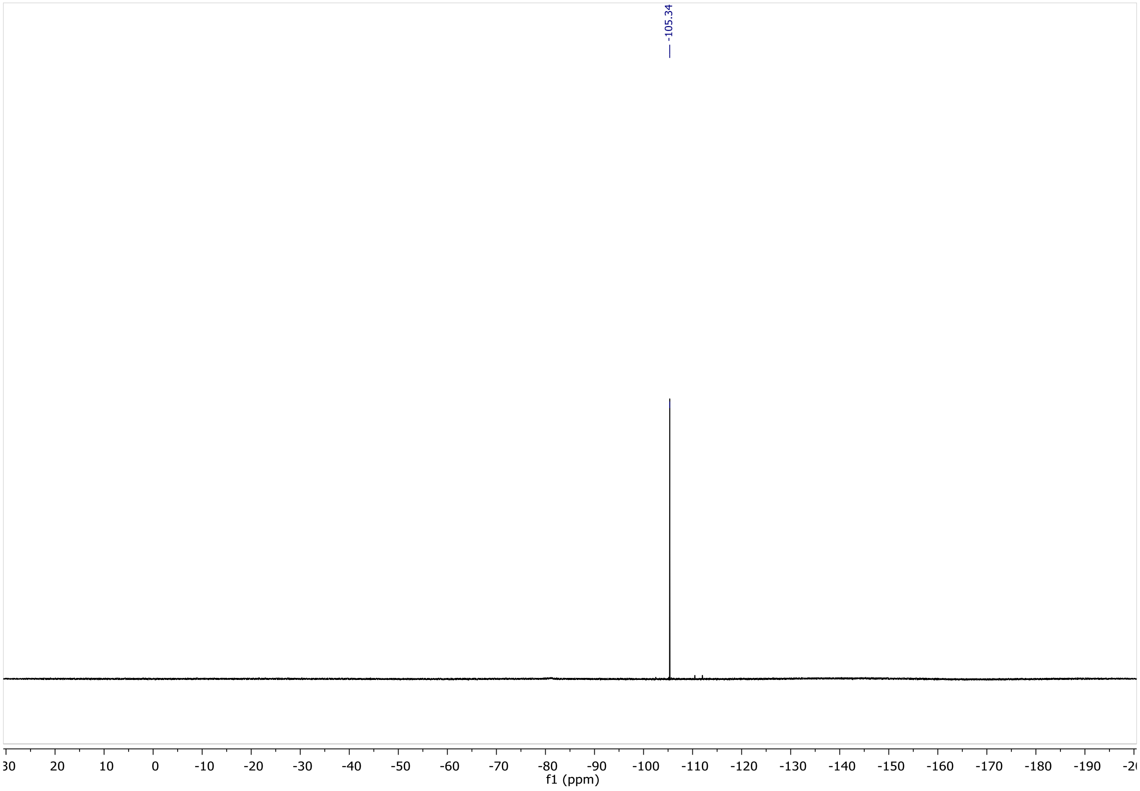

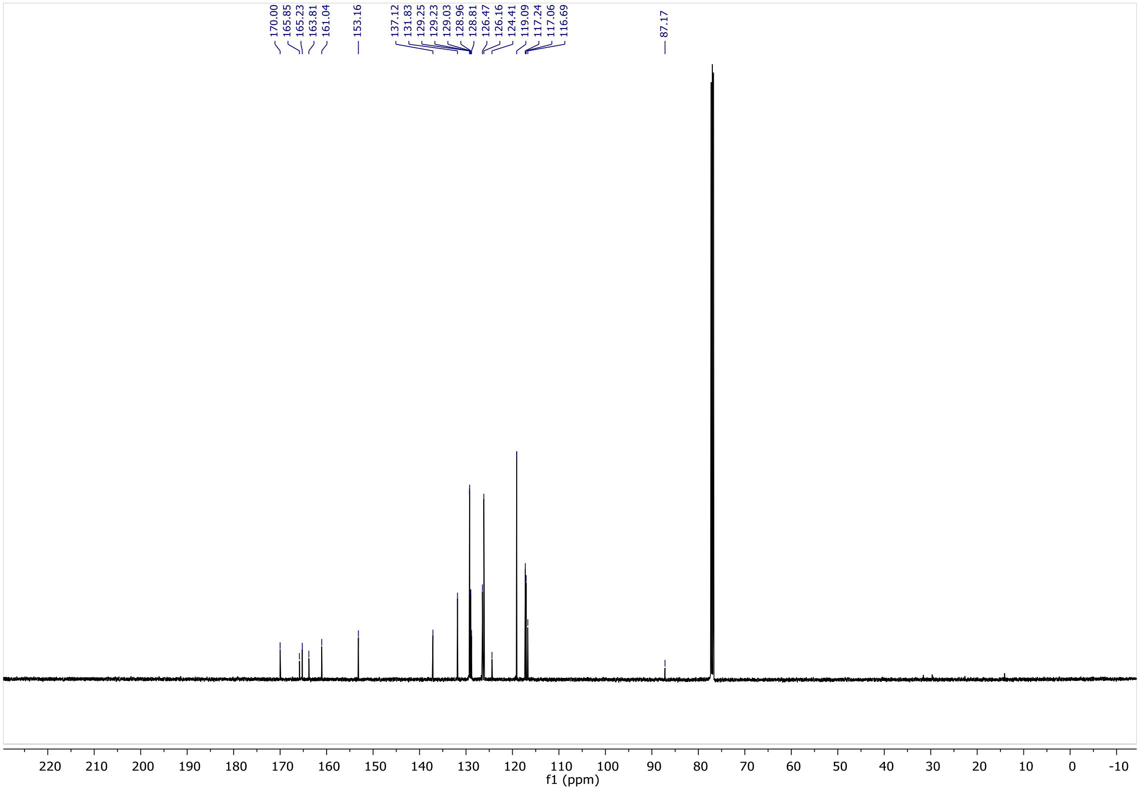

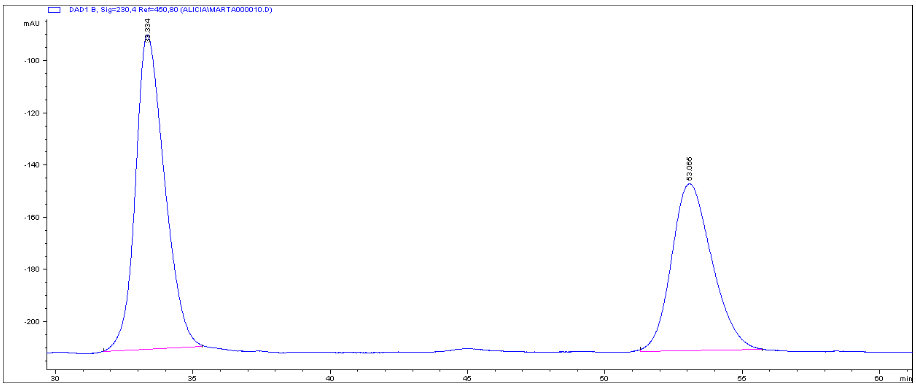

	Time	Area	Area%
1	33.328	12032	**57.489**
2	53.07	8897	**42.511**

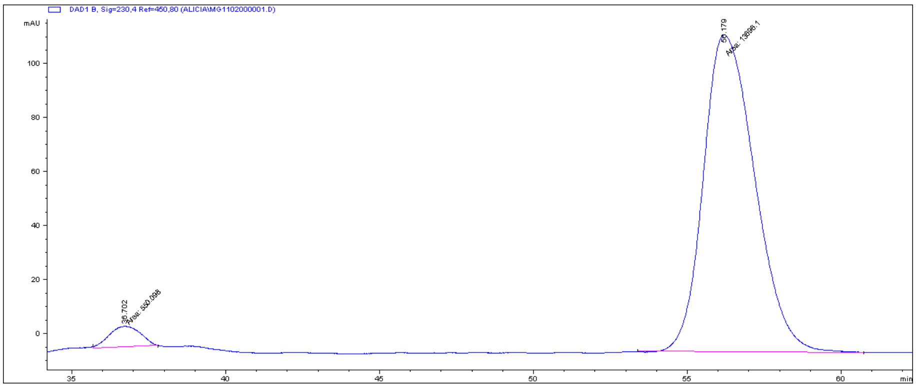

	Time	Area	Area%
1	36.702	550.1	**3.861**
2	56.179	13696.1	**96.139**

Copies of ^{1}H NMR, ^{13}C{^{1}H} NMR and HPLC profiles of 3af

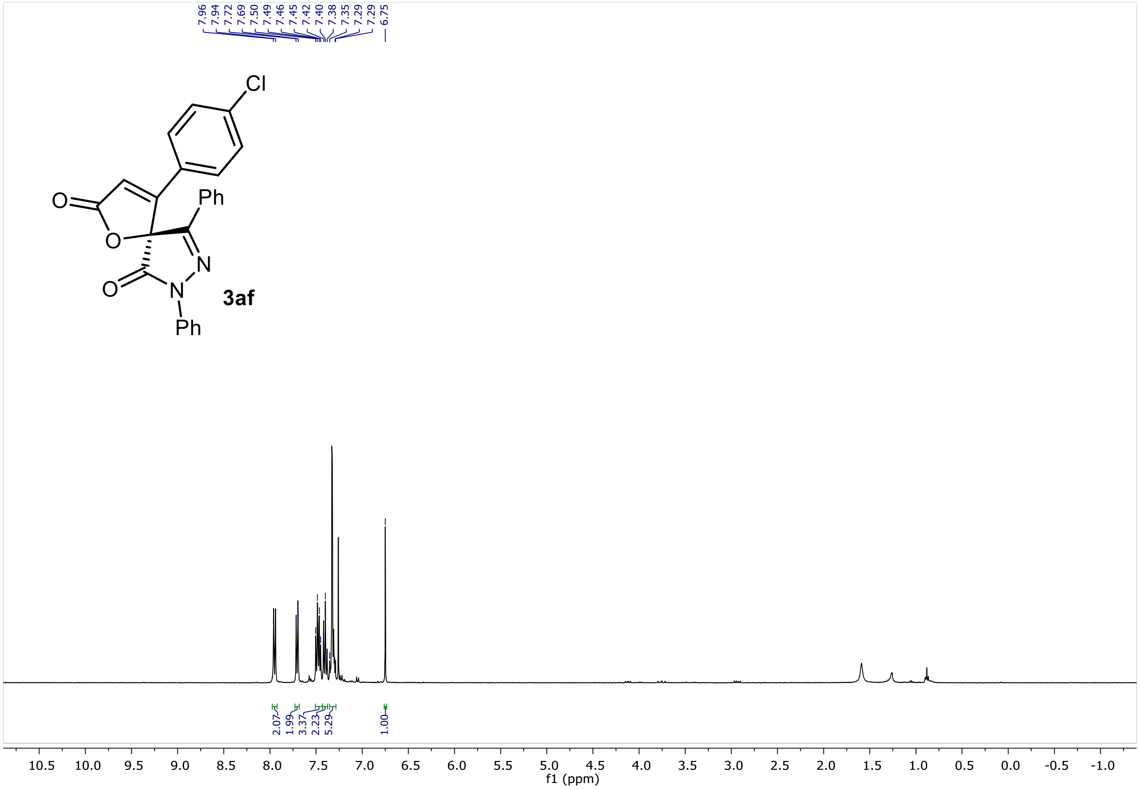

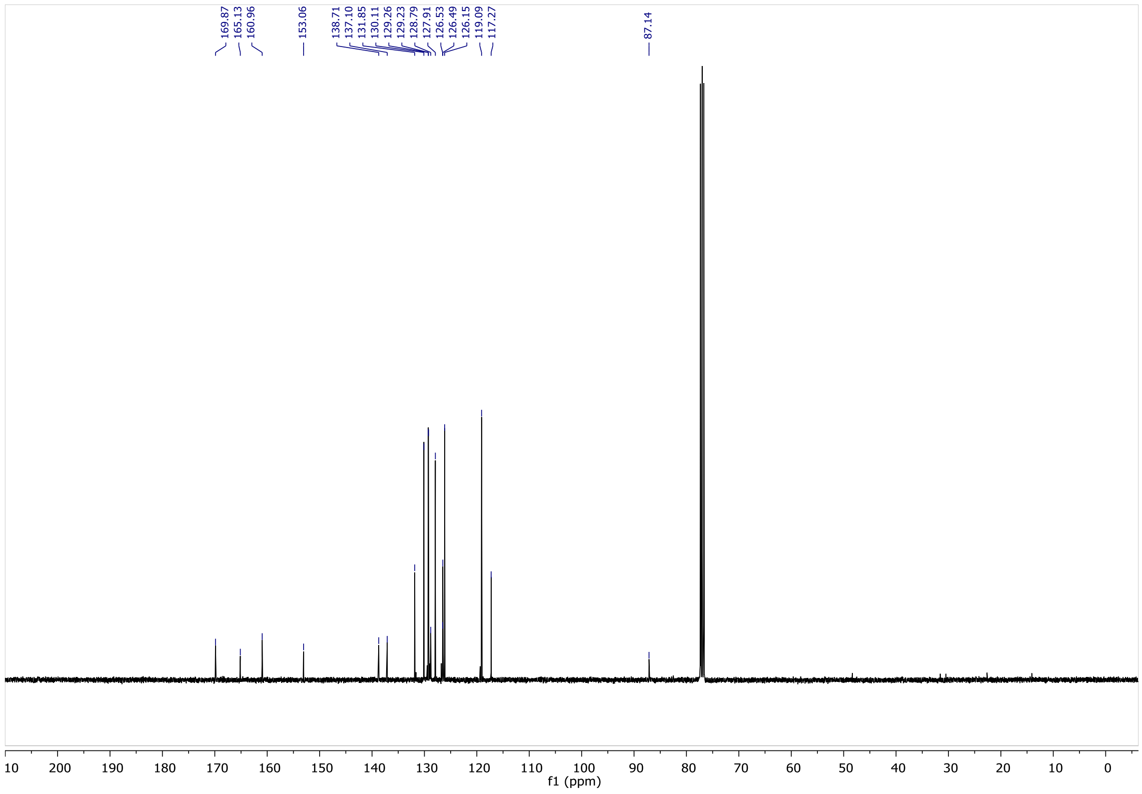

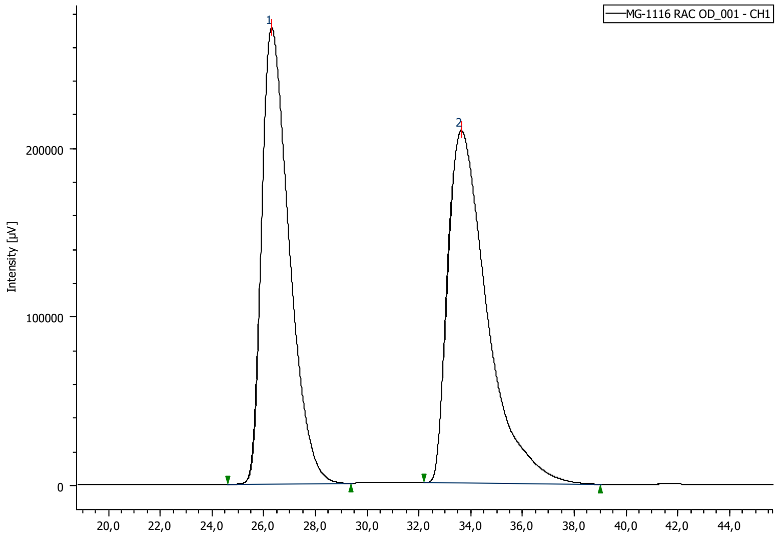

	Time	Area	Area%
1	26,300	19909821	**47,324**
2	33,617	22161830	**52,676**

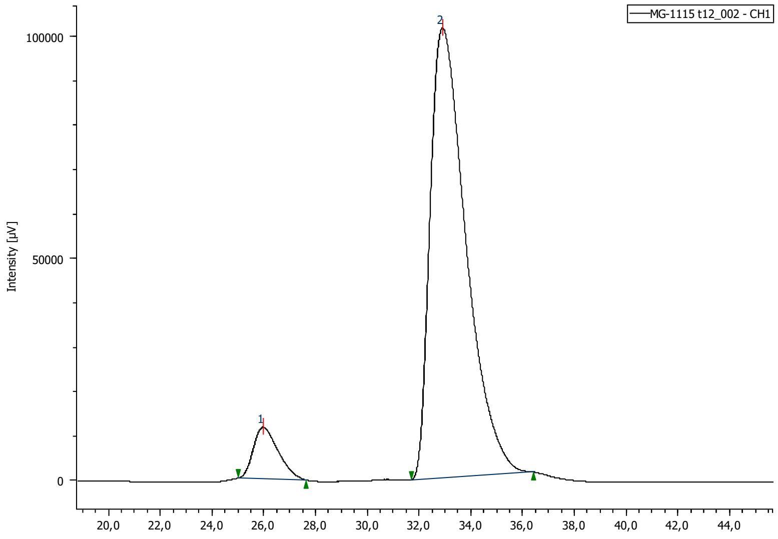

	Time	Area	Area%
1	25,967	770044	**7,332**
2	32,892	9732222	**92,668**

Copies of ^{1}H NMR, ^{13}C{^{1}H} NMR and HPLC profiles of 3ag

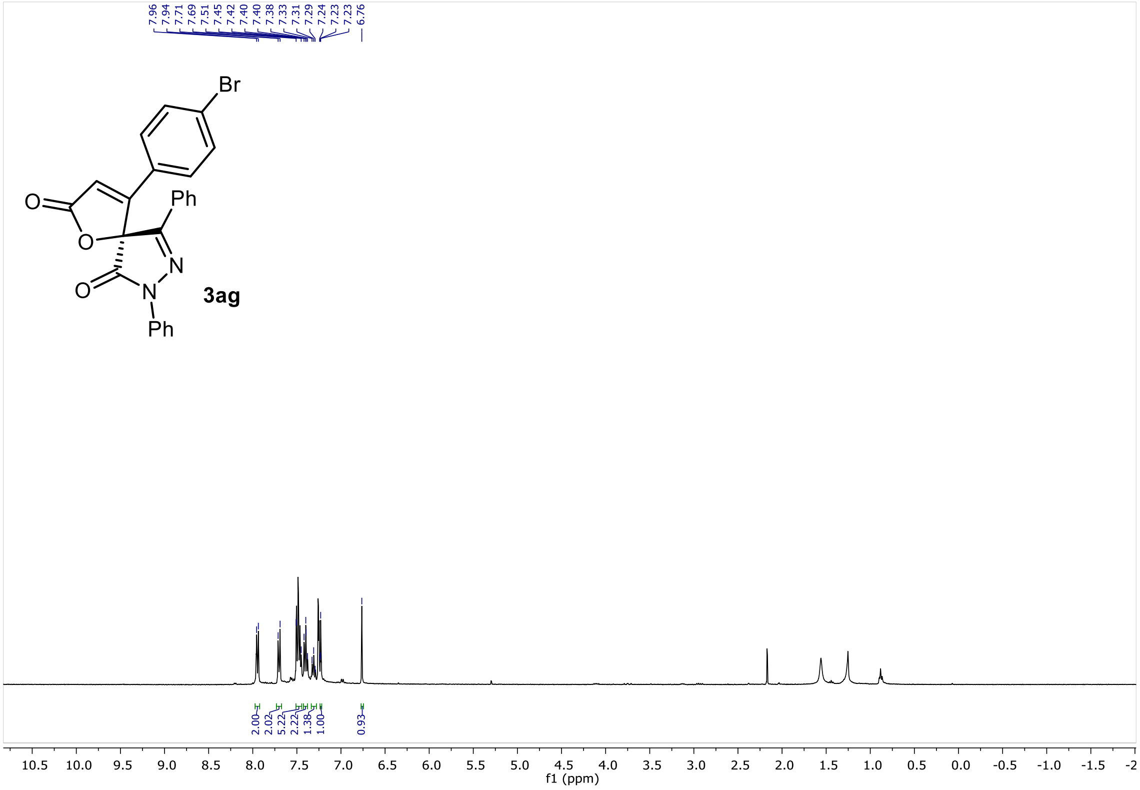

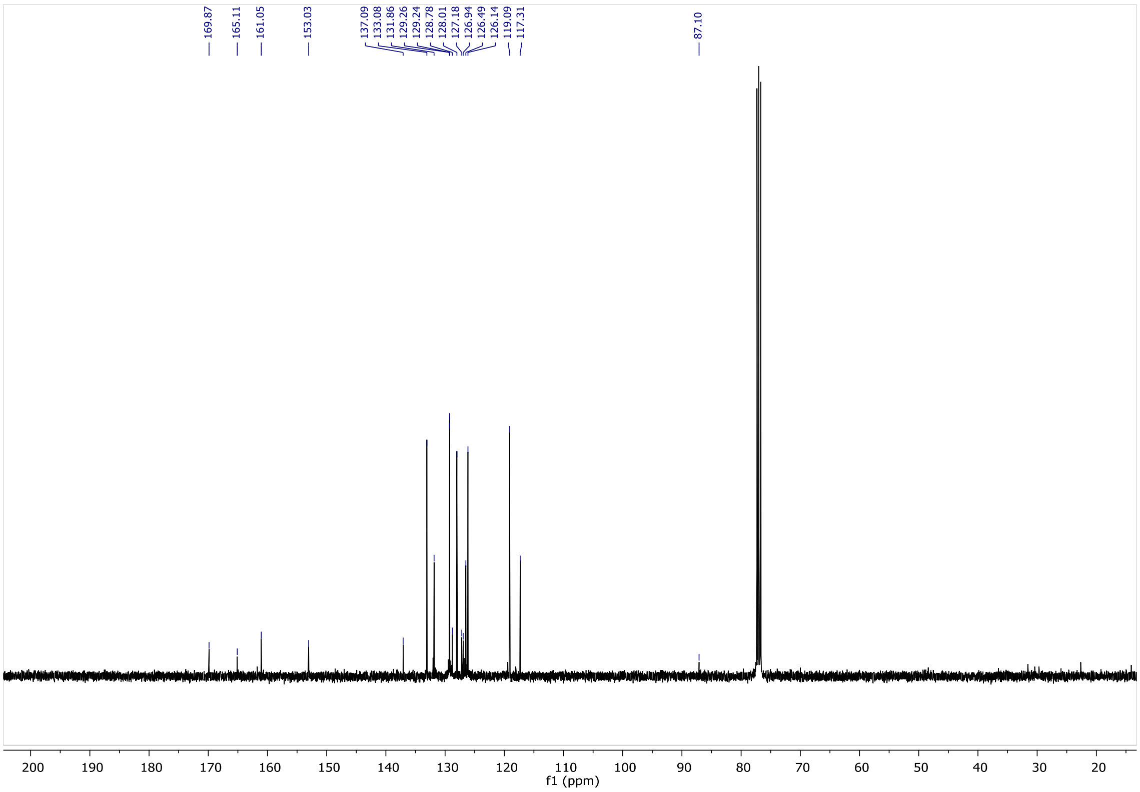

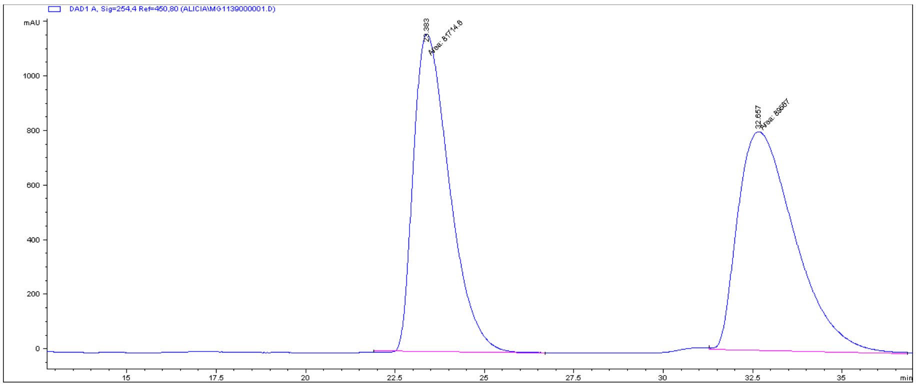

	Time	Area	Area%
1	23.383	81714.6	**47.708**
2	32.657	89567	**52.292**

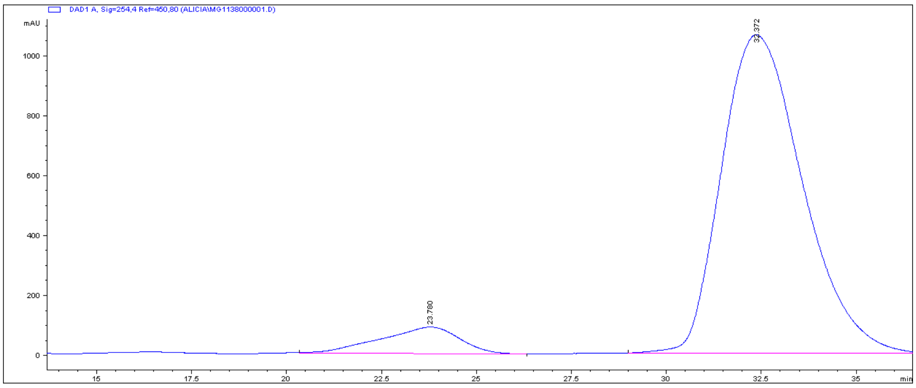

#	Time	Area	Area%
1	23.78	13223.9	**7.737**
2	32.372	157687.3	**92.263**

Copies of ^{1}H NMR, ^{13}C{^{1}H} NMR and HPLC profiles of 3ah

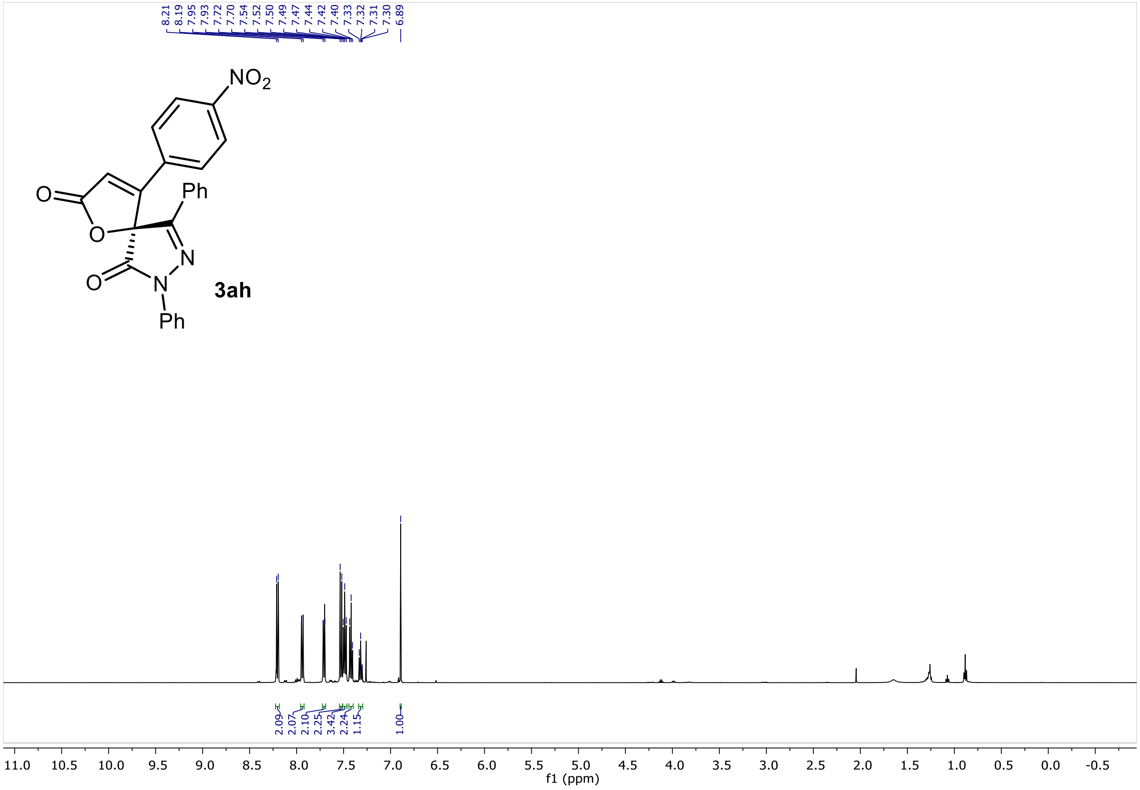

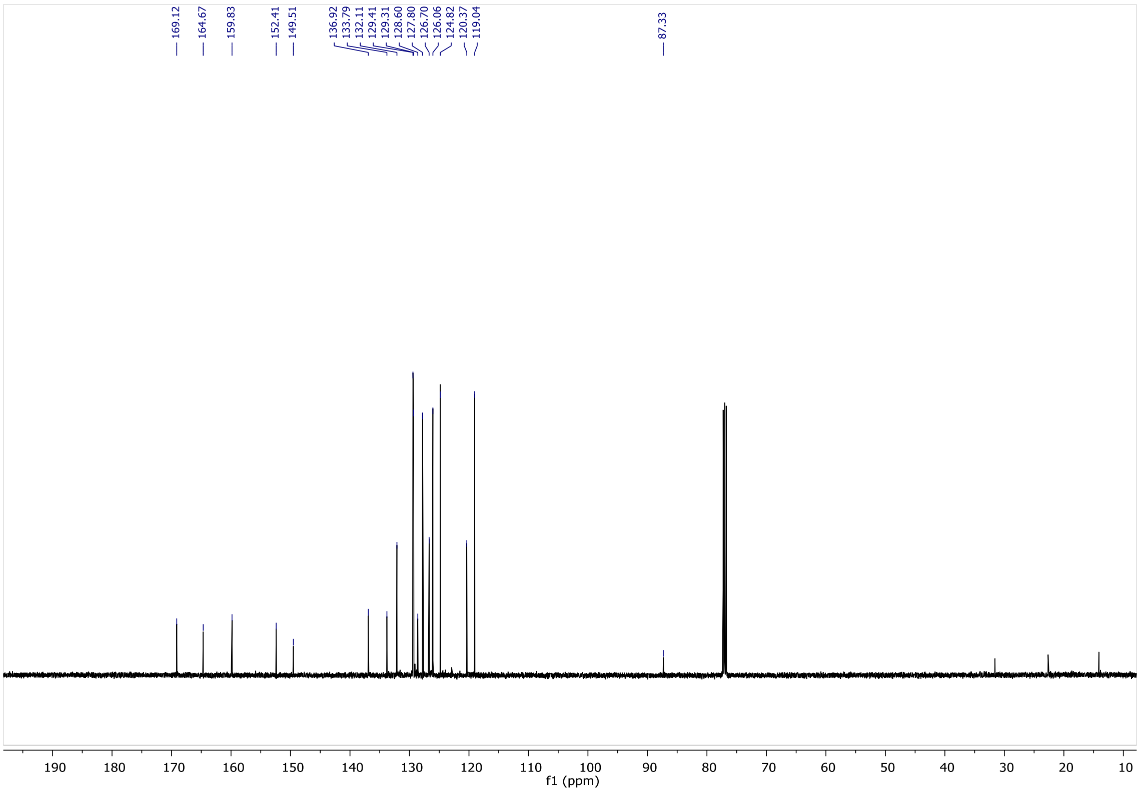

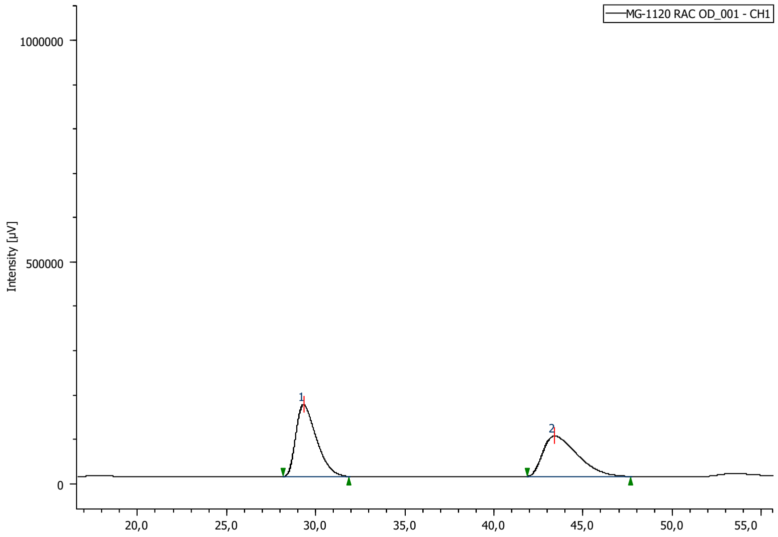

	Time	Area	Area%
1	29,300	12994628	**51,467**
2	43,383	12253764	**48,533**

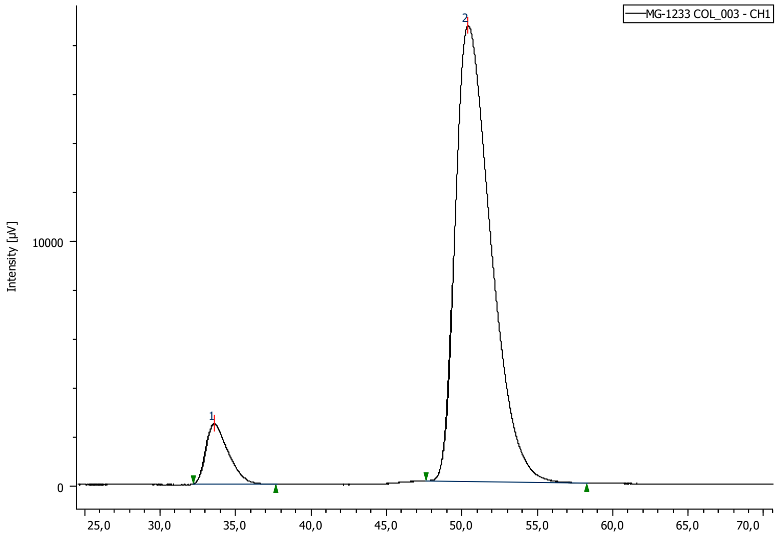

	Time	Area	Area%
1	33,583	256346	**7,766**
2	50,383	3044423	**92,234**

Copies of ^{1}H NMR, $^{13}C\{^{1}H\}$ NMR and HPLC profiles of 3ai

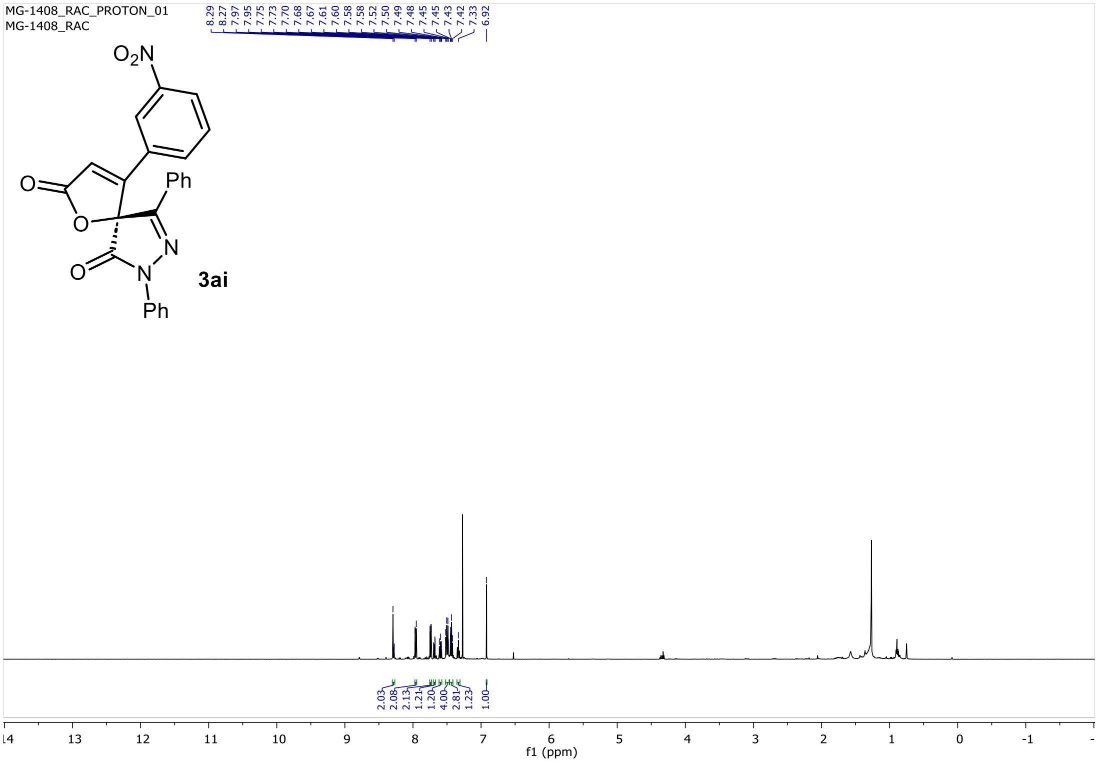

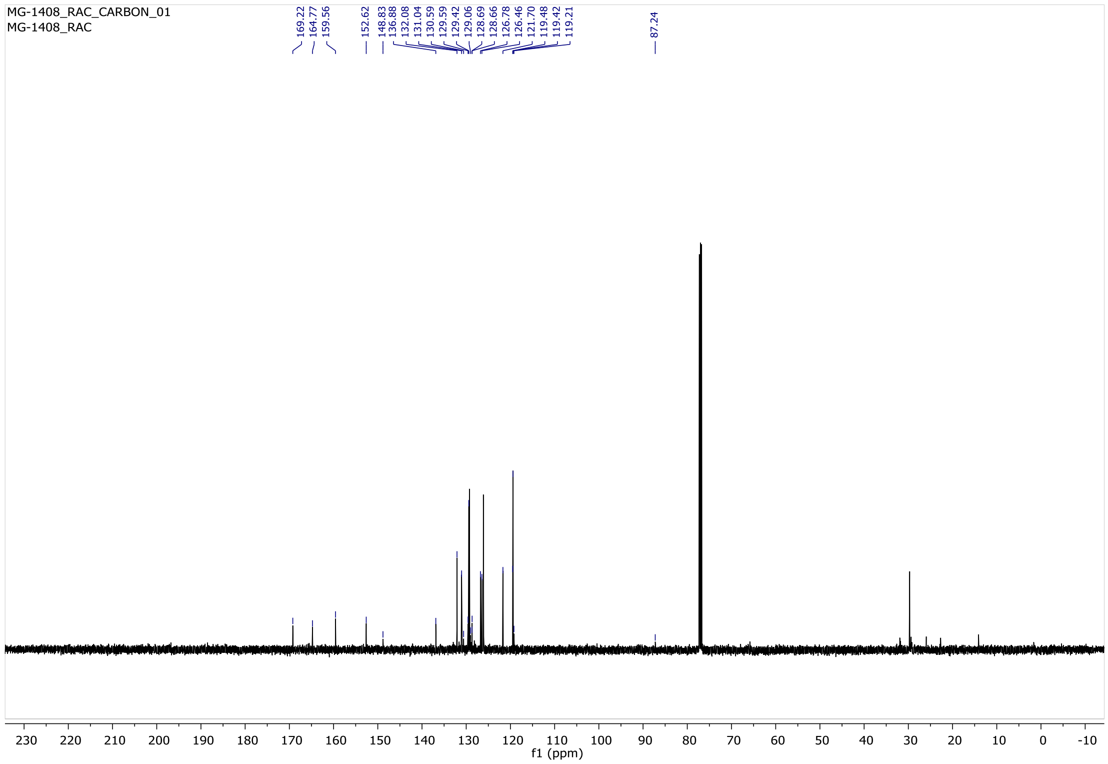

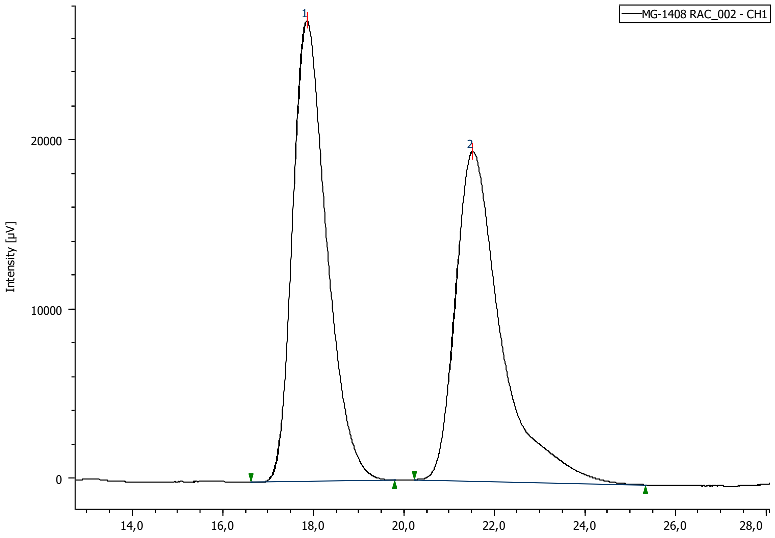

	t_R	Area	Area%
1	17,850	1421804	**51,127**
2	21,517	1359124	**48,873**

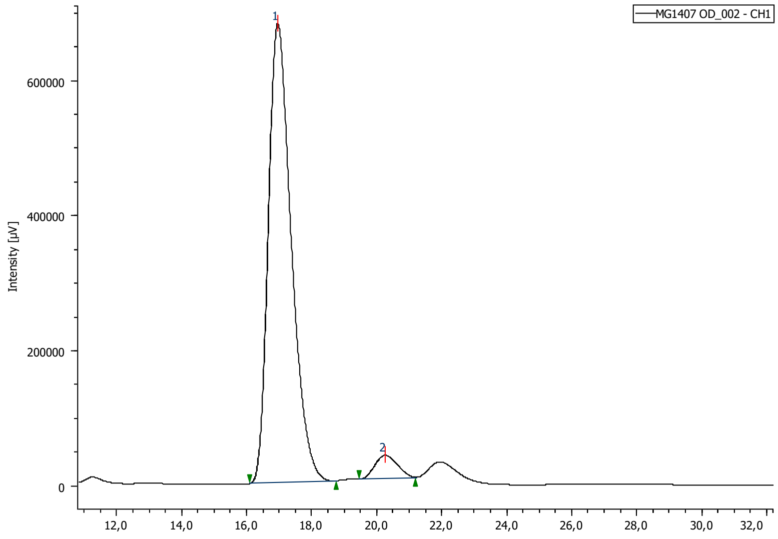

	t_R	Area	Area%
1	16,950	33222690	**95,334**
2	20,250	1626061	**4,666**

Copies of ^{1}H NMR, ^{13}C{^{1}H} NMR and HPLC profiles of 3aj

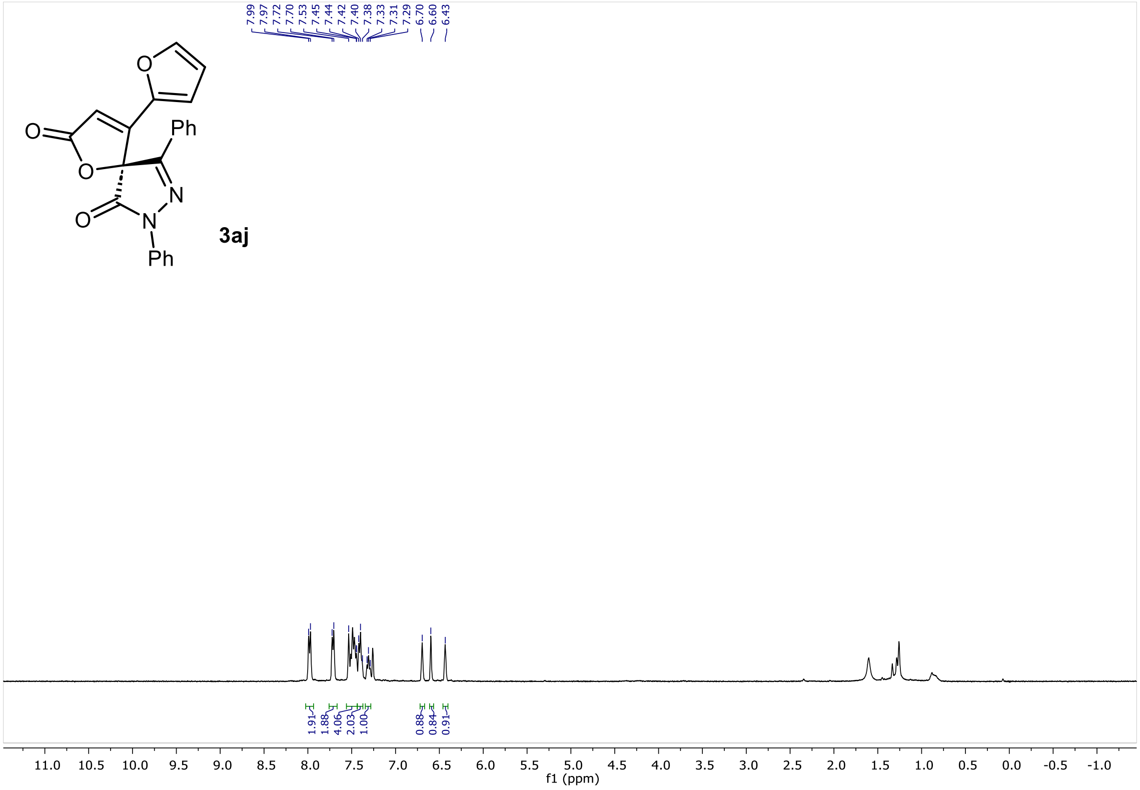

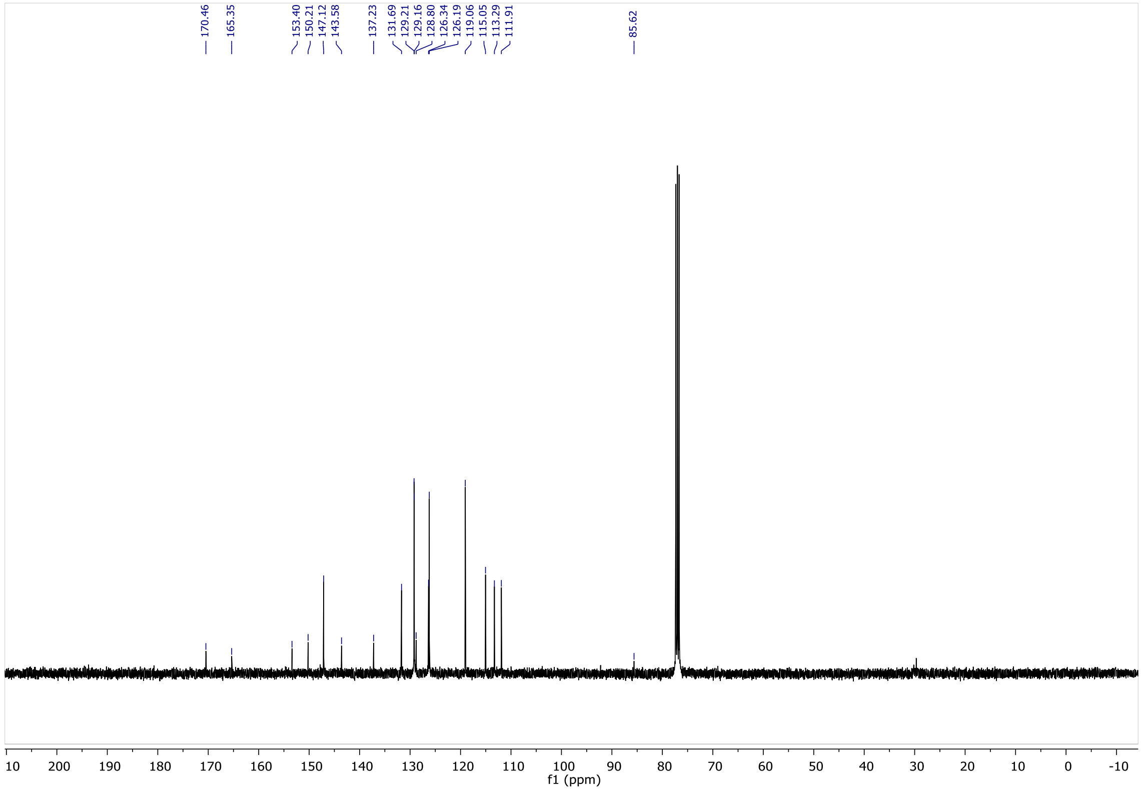

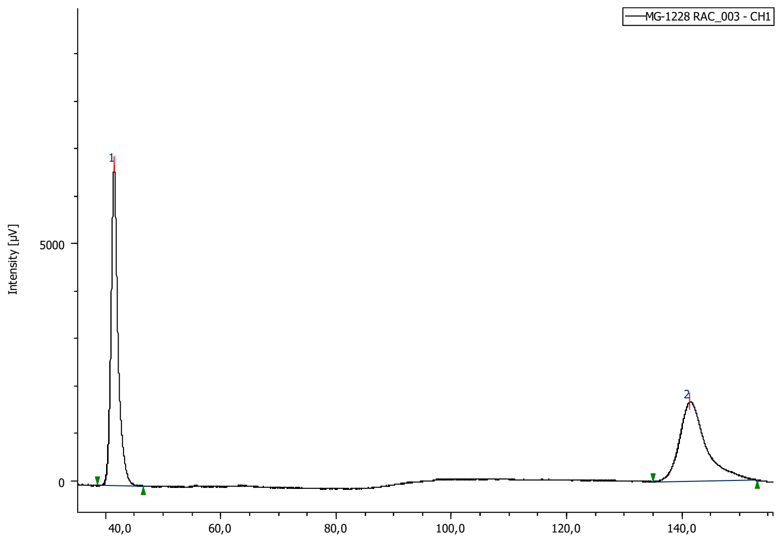

	Time	Area	Area%
1	41,467	563602	**51,411**
2	141,283	532673	**48,589**

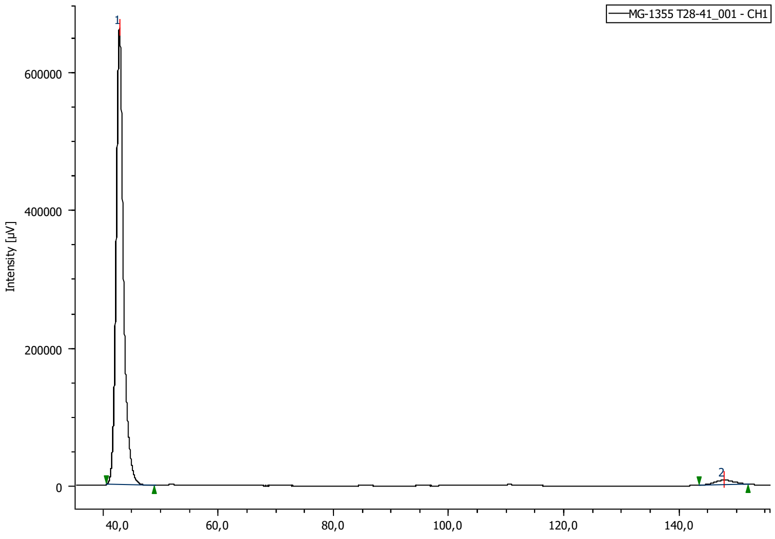

	Time	Area	Area%
1	42,875	58457355	**97,296**
2	147,683	1624379	**2,704**

Copies of ^{1}H NMR, ^{13}C{^{1}H} NMR and HPLC profiles of 3ak

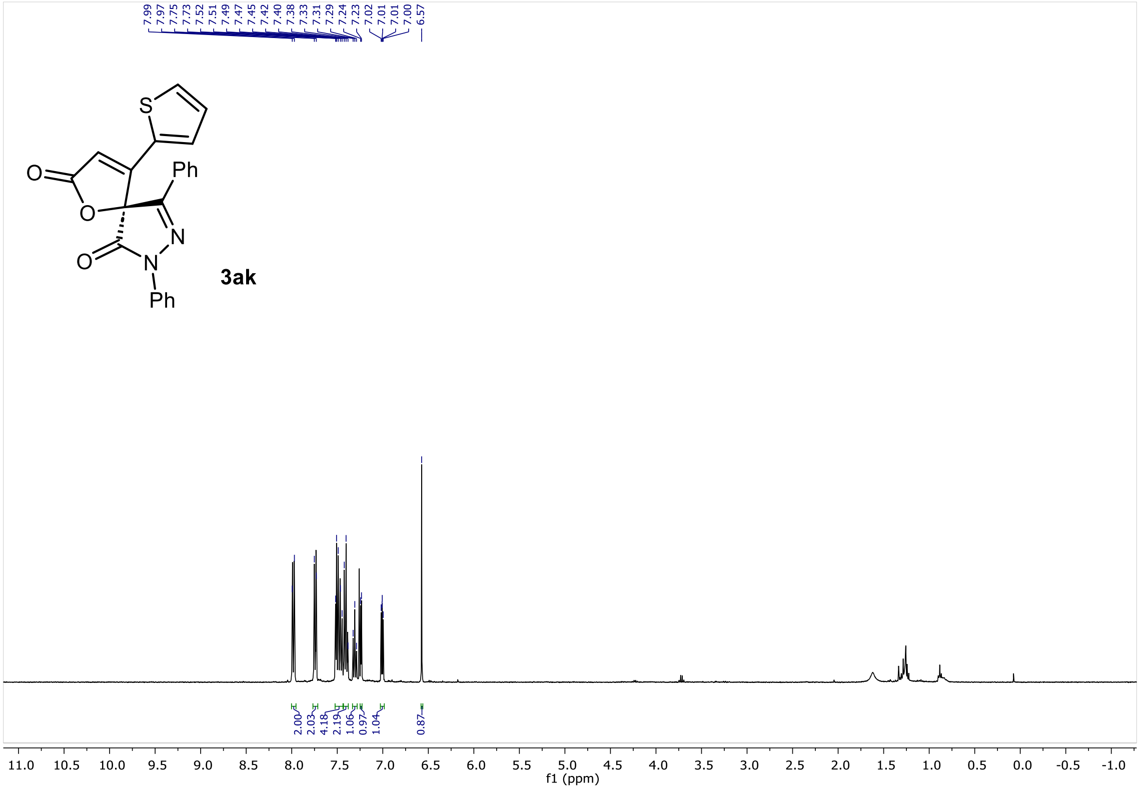

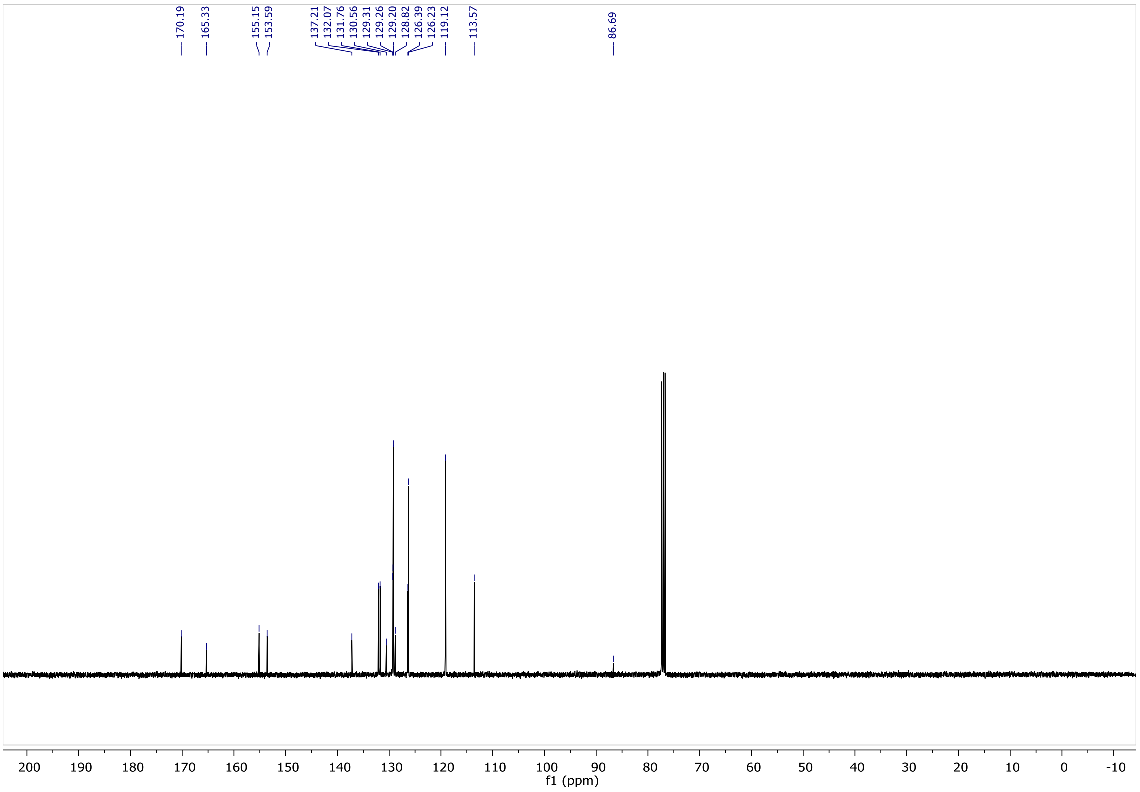

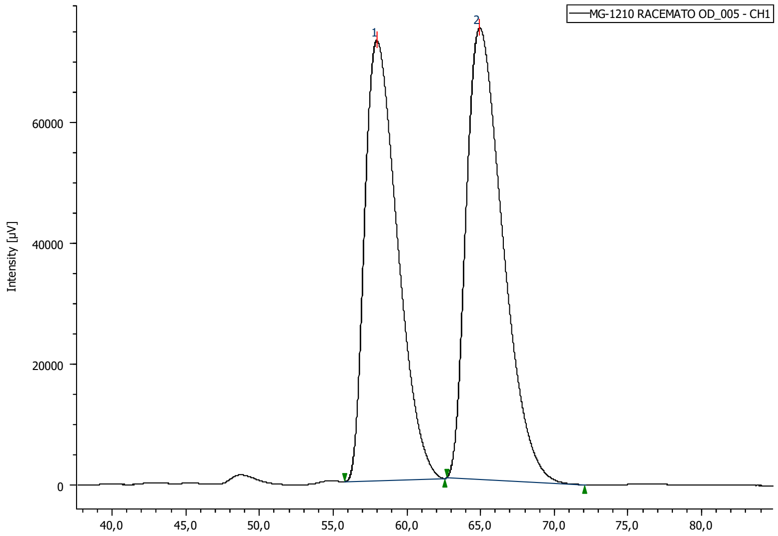

	Time	Area	Area%
1	57,942	11269998	**46,616**
2	64,900	12906201	**53,384**

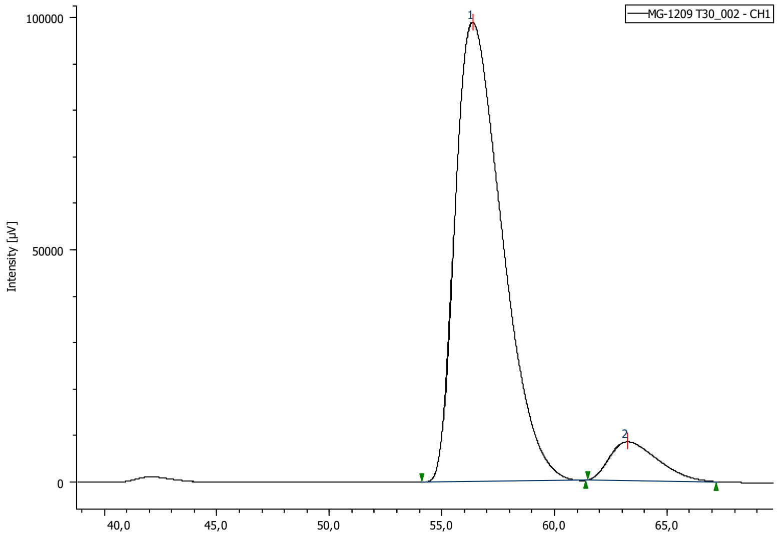

	Time	Area	Area%
1	56,342	14292691	**92,068**
2	63,217	1231385	**7,932**

Copies of ^{1}H NMR, ^{13}C{^{1}H} NMR and HPLC profiles of 3al

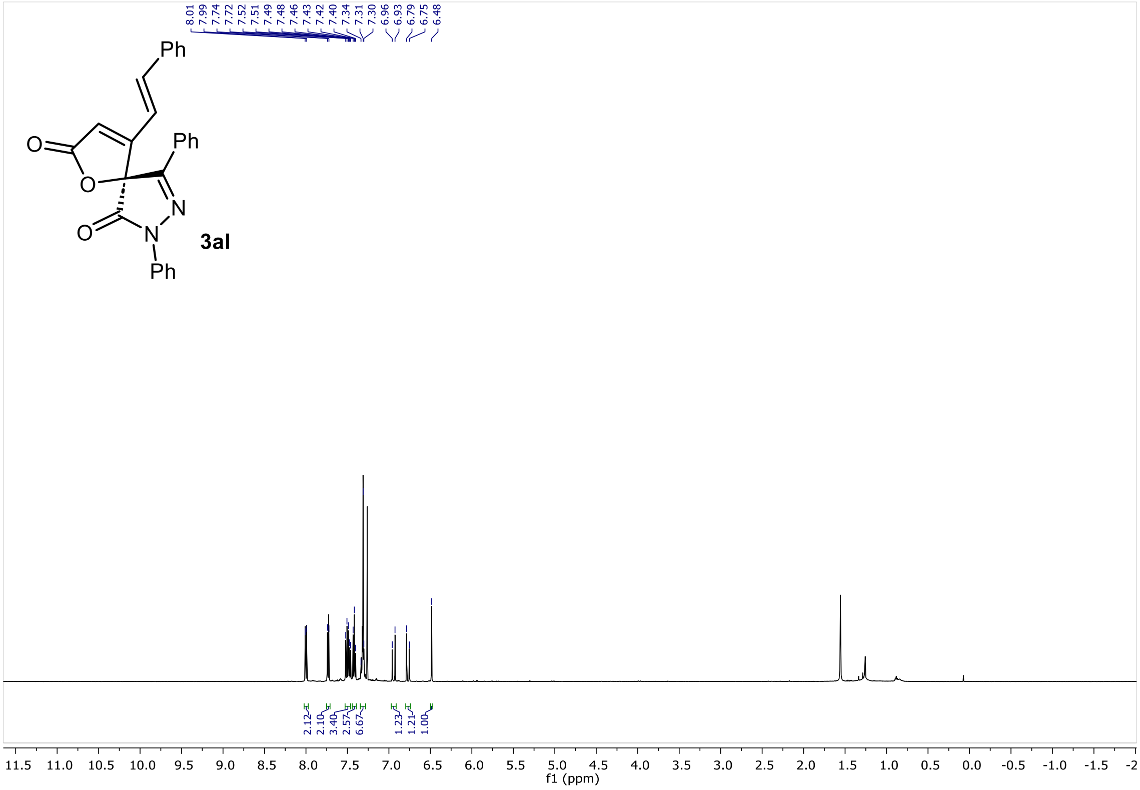

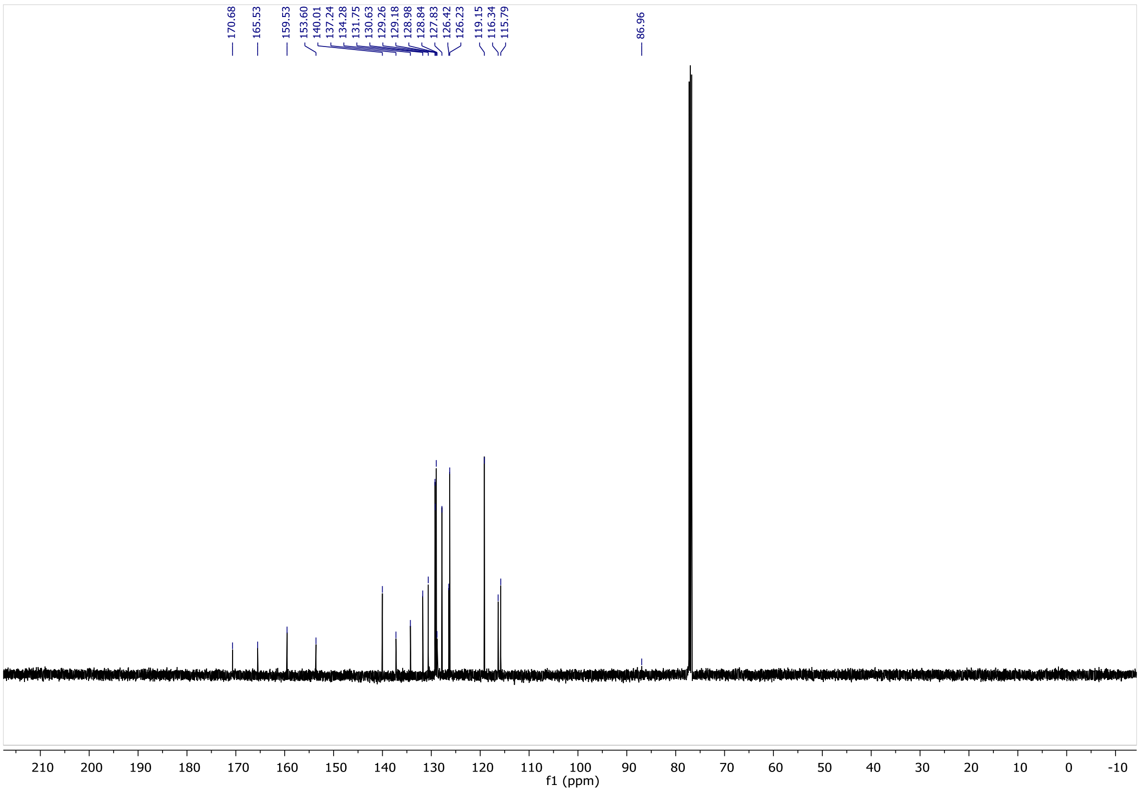

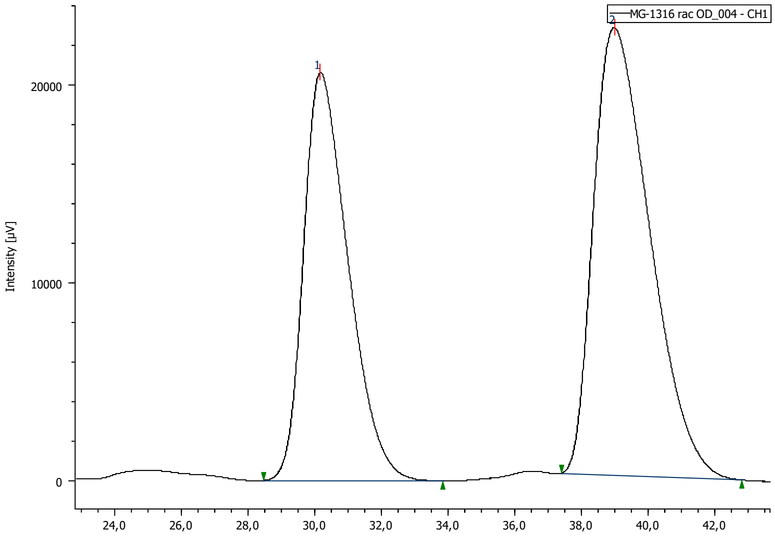

	Time	Area	Area%
1	30,158	1918677	**41,961**
2	38,967	2653820	**58,039**

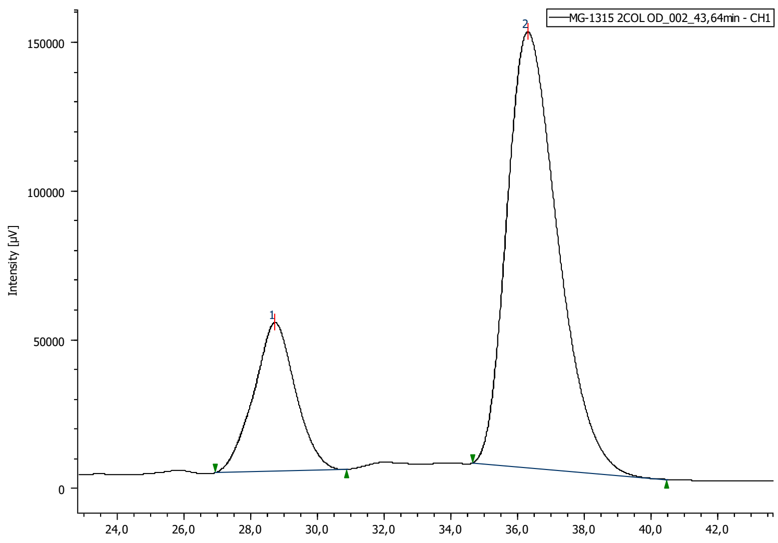

	Time	Area	Area%
1	28,725	4333432	**21,804**
2	36,292	15540784	**78,196**

Copies of ^{1}H NMR, ^{13}C{^{1}H} NMR, ^{19}F NMR and HPLC profiles of 3ce

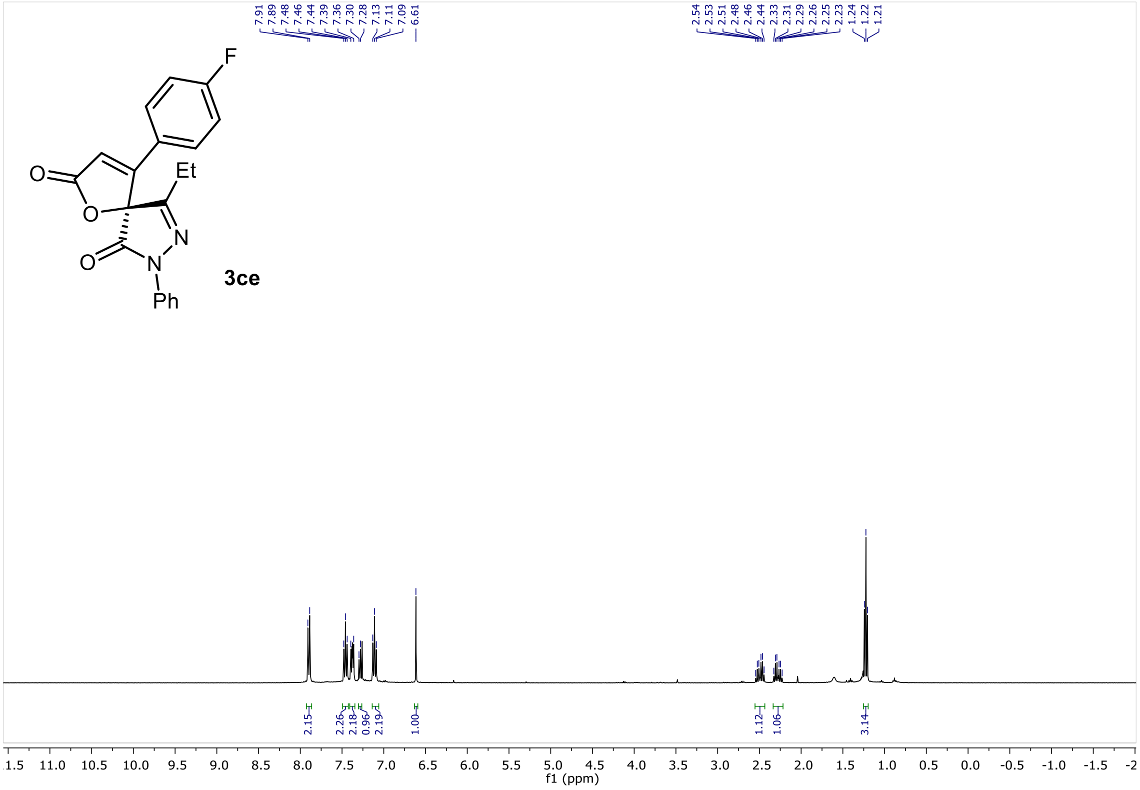

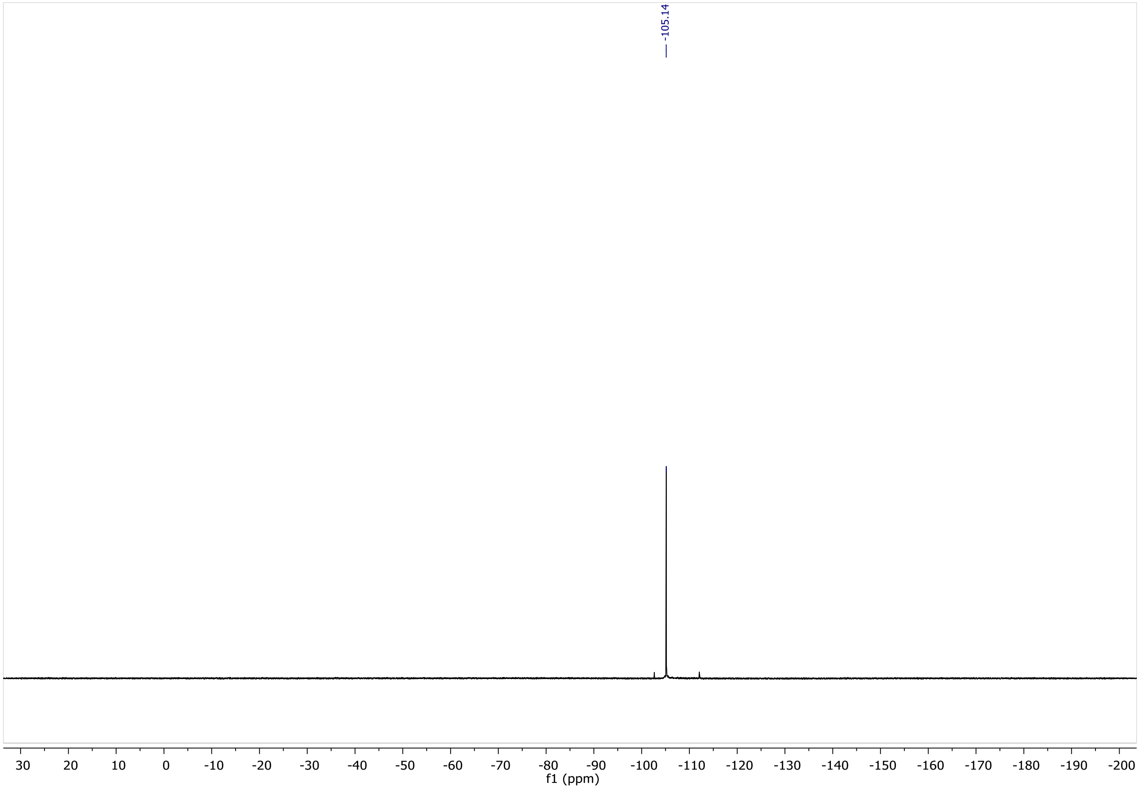

-105.14
30 20 10 0 -10 -20 -30 -40 -50 -60 -70 -80 -90 -100 -110 -120 -130 -140 -150 -160 -170 -180 -190 -200
f1 (ppm)

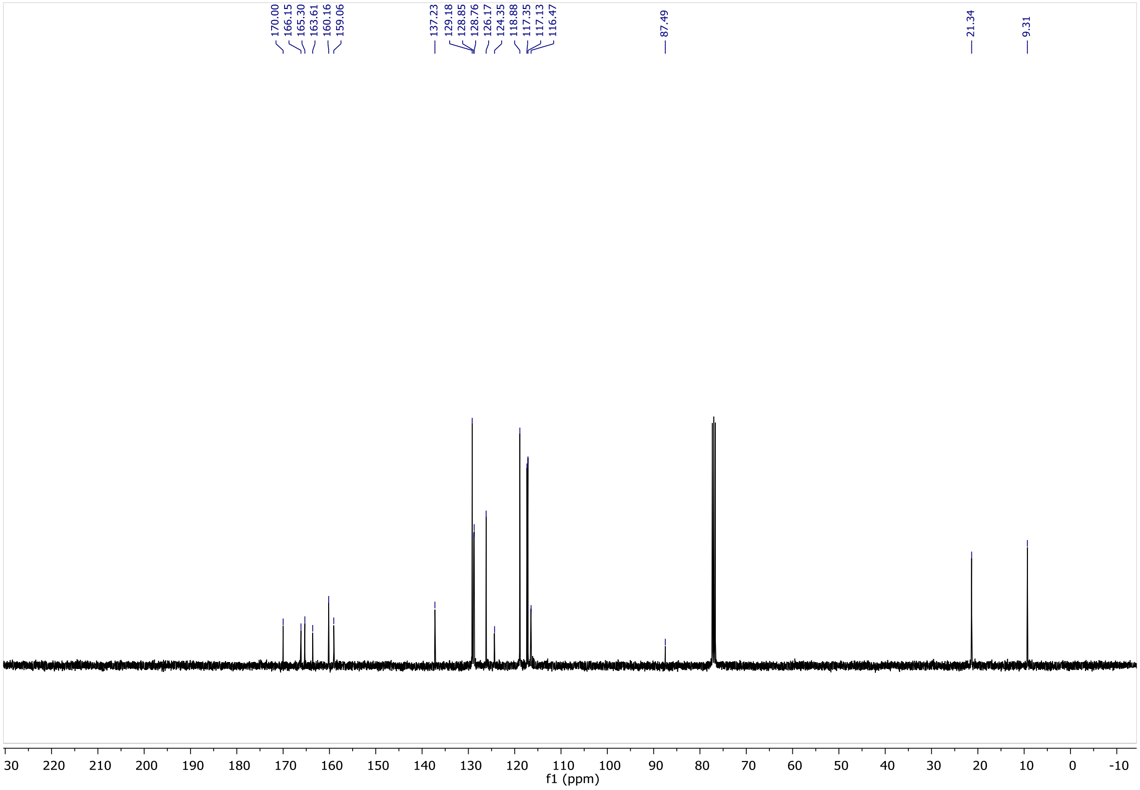

170.00
166.15
165.30
163.61
160.16
159.06
137.23
129.18
128.85
128.76
126.17
124.35
118.88
117.35
117.13
116.47
87.49
21.34
9.31
30 220 210 200 190 180 170 160 150 140 130 120 110 100 90 80 70 60 50 40 30 20 10 0 -10
f1 (ppm)

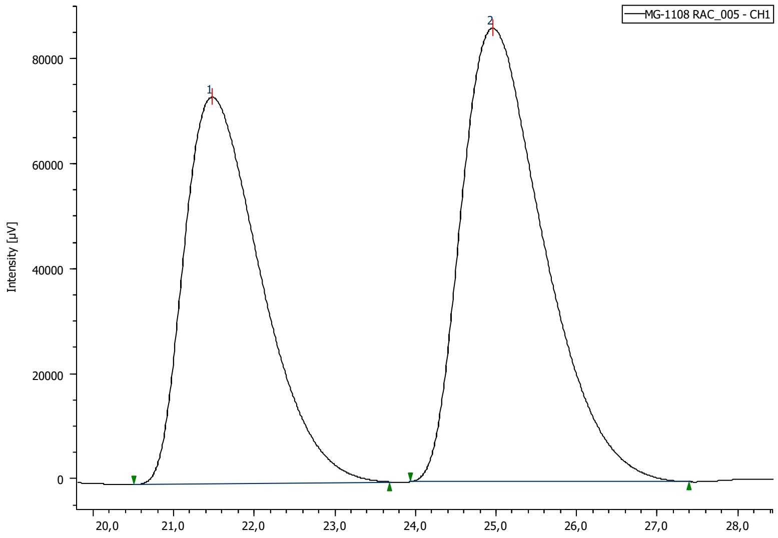

	Time	Area	Area%
1	21,475	4878584	**44,203**
2	24,950	6158087	**55,797**

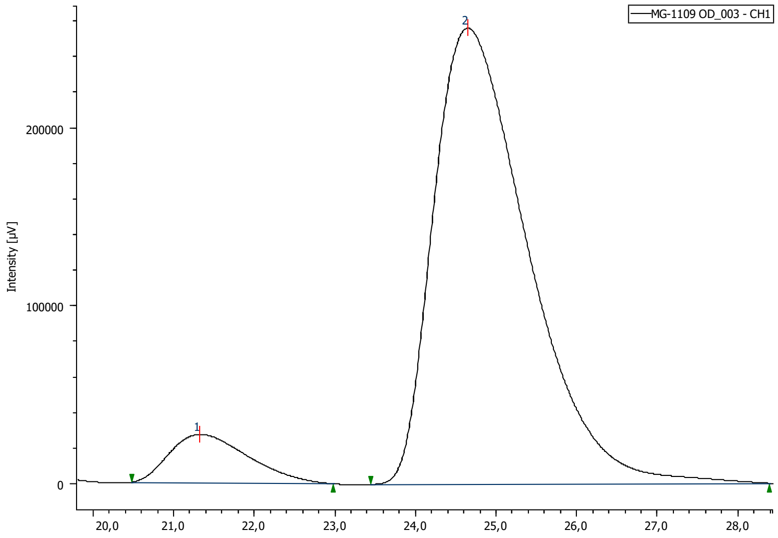

	Time	Area	Area%
1	21,325	1843137	**8,147**
2	24,642	20779722	**91,853**

Copies of ^{1}H NMR, $^{13}C\{^{1}H\}$ NMR and HPLC profiles of 3db

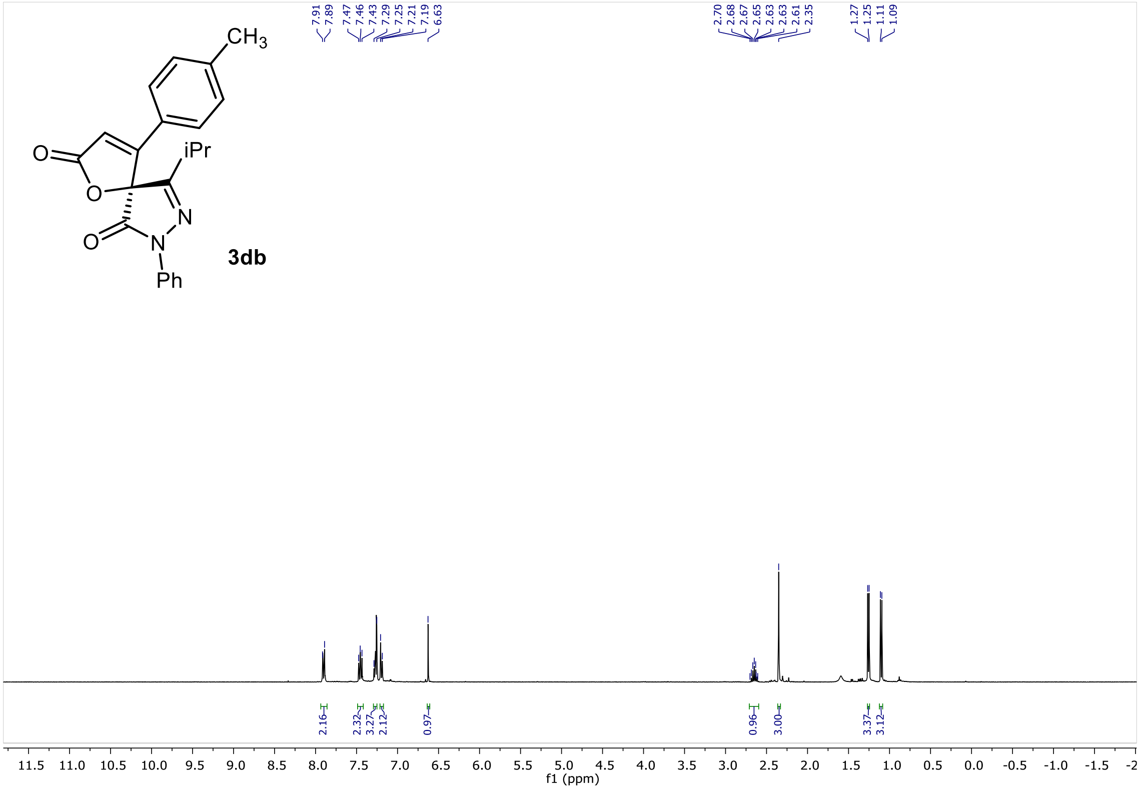

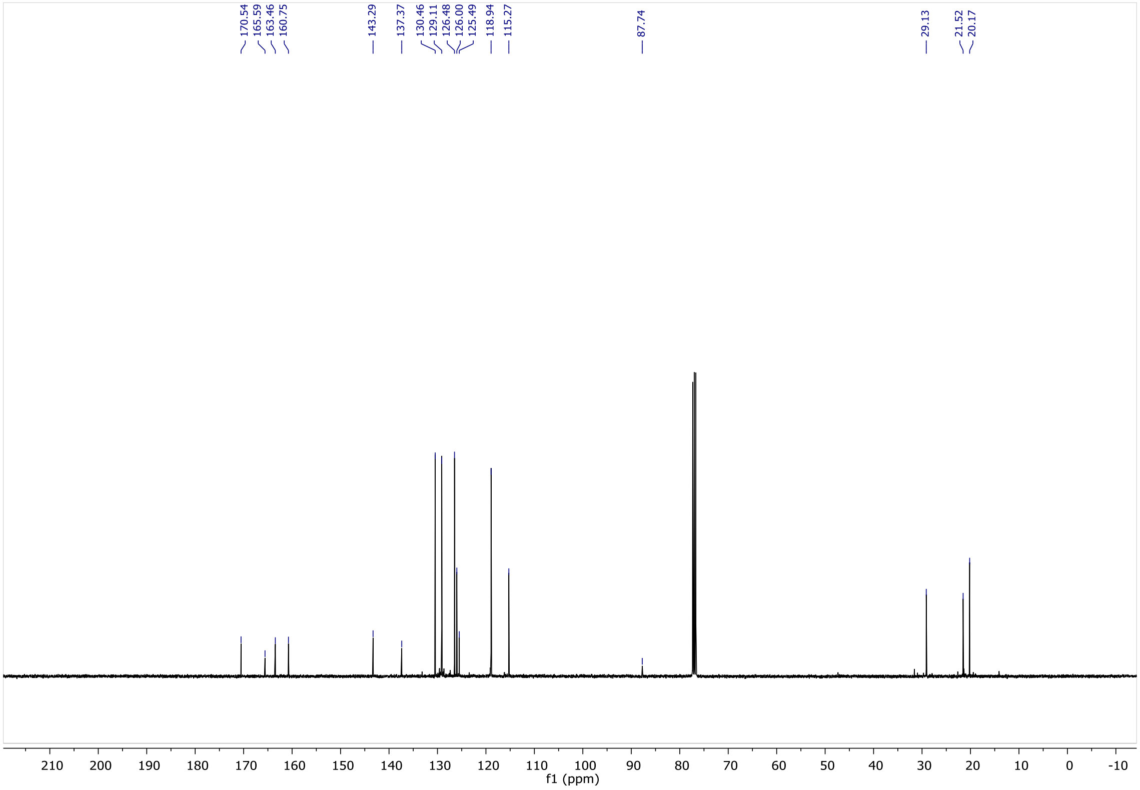

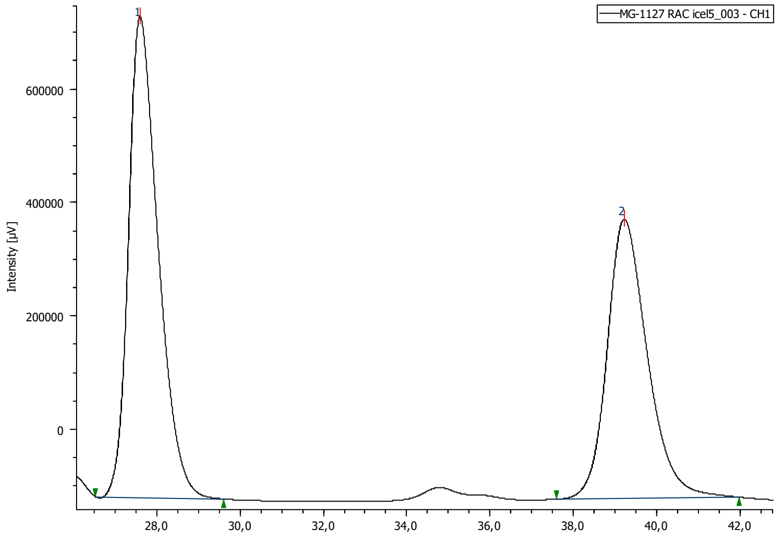

	Time	Area	Area%
1	27,600	41374349	**55,717**
2	39,200	32883630	**44,283**

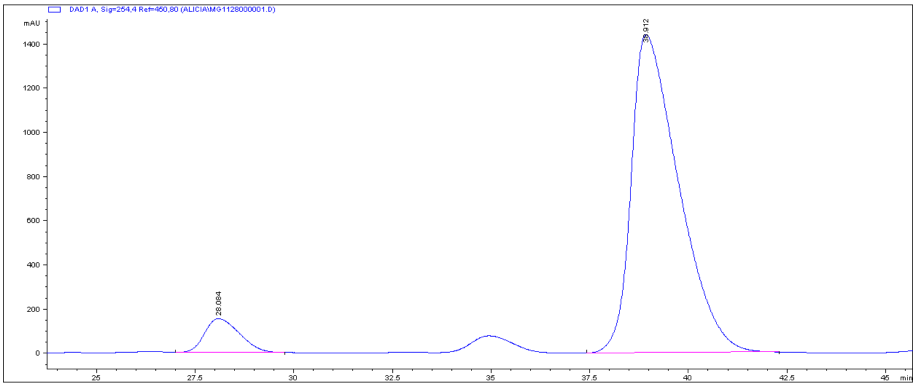

	Time	Area	Area%
1	28.084	9460.3	**7.279**
2	38.912	120509.7	**92.721**

Copies of ^{1}H NMR, $^{13}C\{^{1}H\}$ NMR and HPLC profiles of 3df

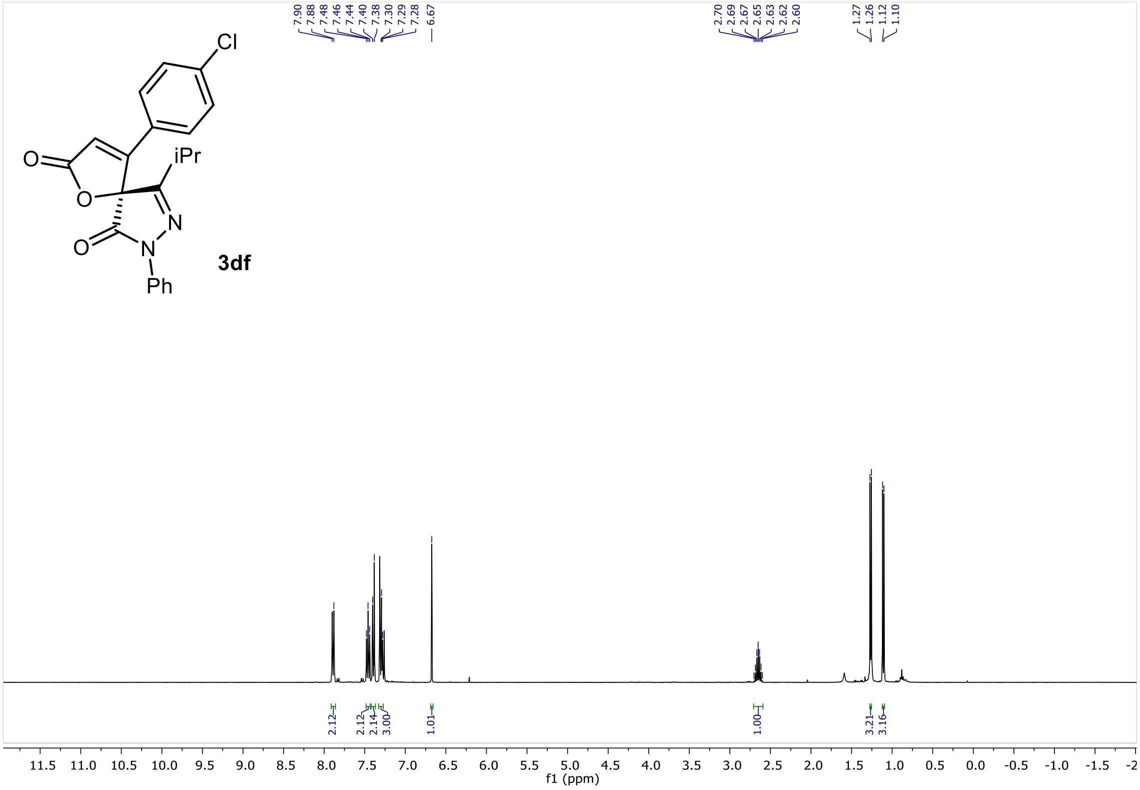

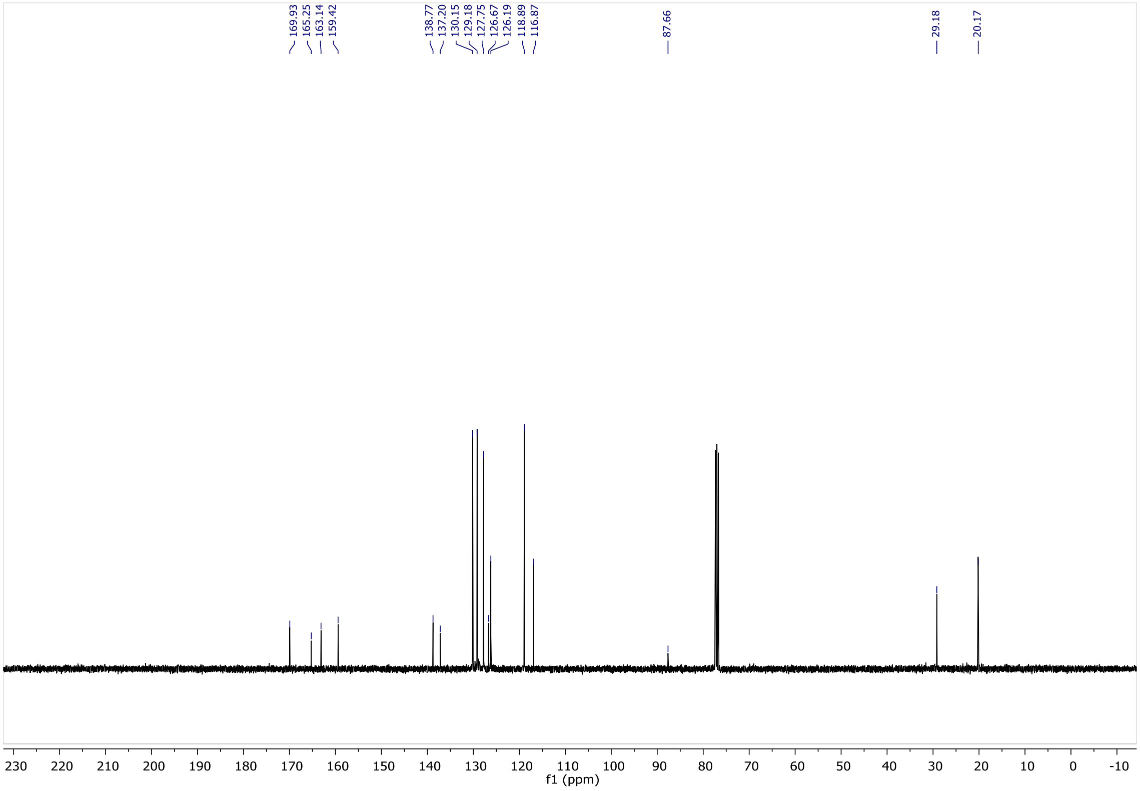

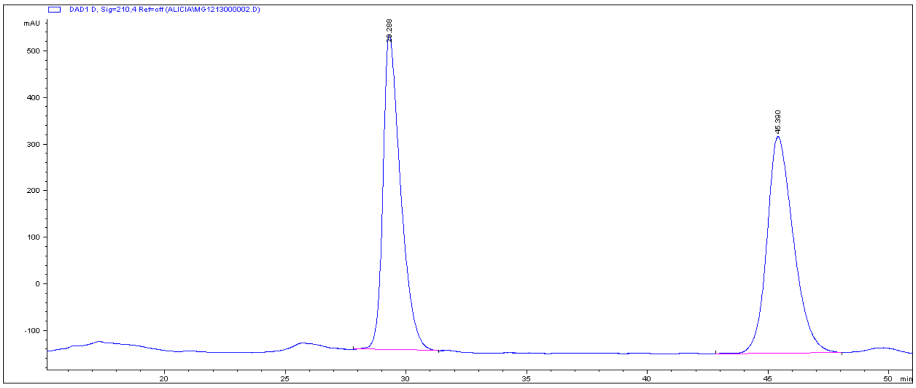

	Time	Area	Area%
1	29.288	34438.6	**49.168**
2	45.39	35604.5	**50.832**

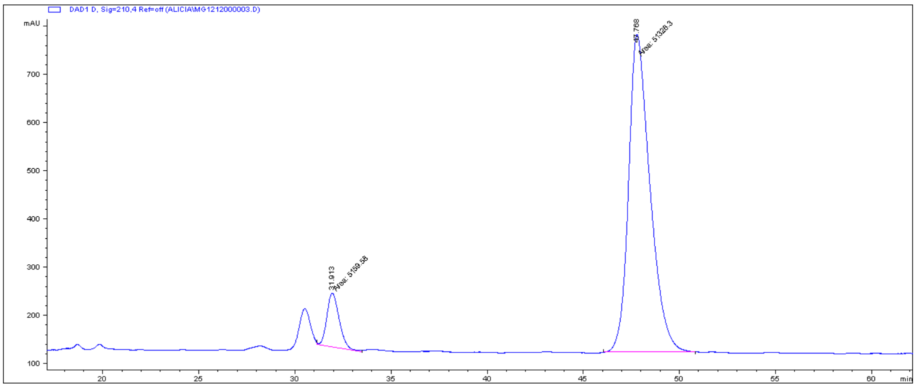

	Time	Area	Area%
1	31.913	5159.6	**9.134**
2	47.768	51326.3	**90.866**

Copies of ^{1}H NMR, ^{13}C{^{1}H} NMR and HPLC profiles of 4

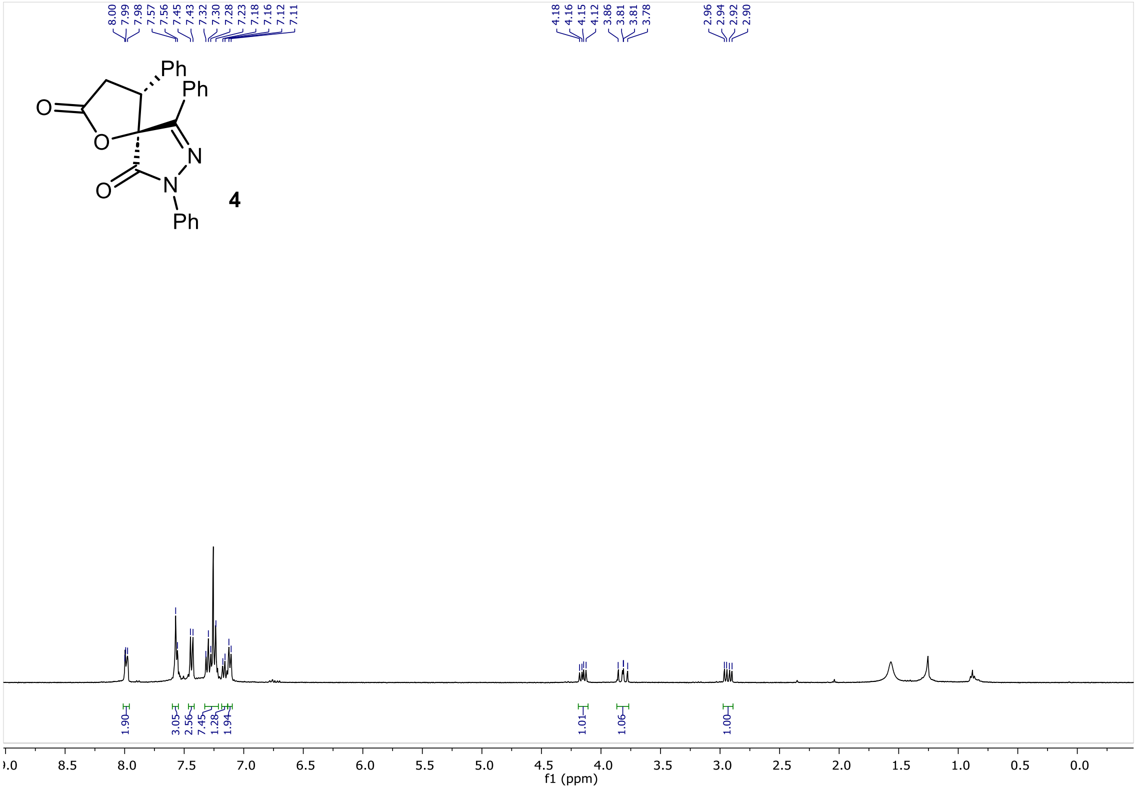

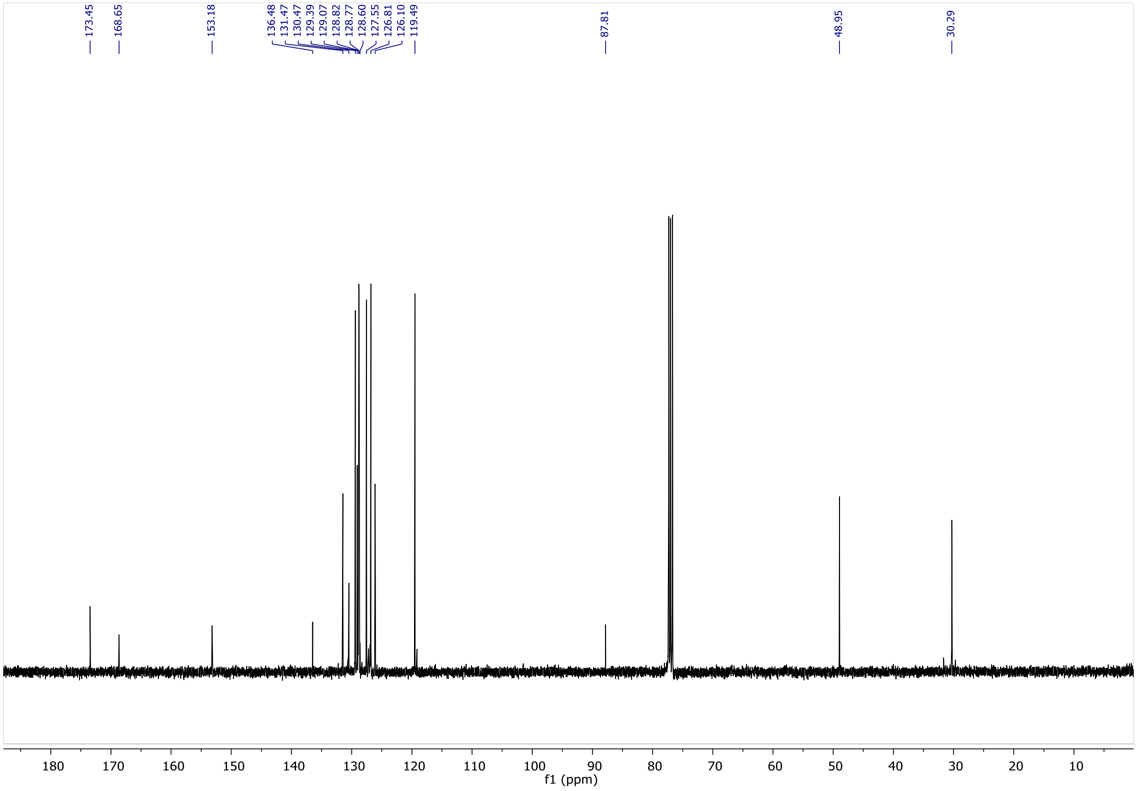

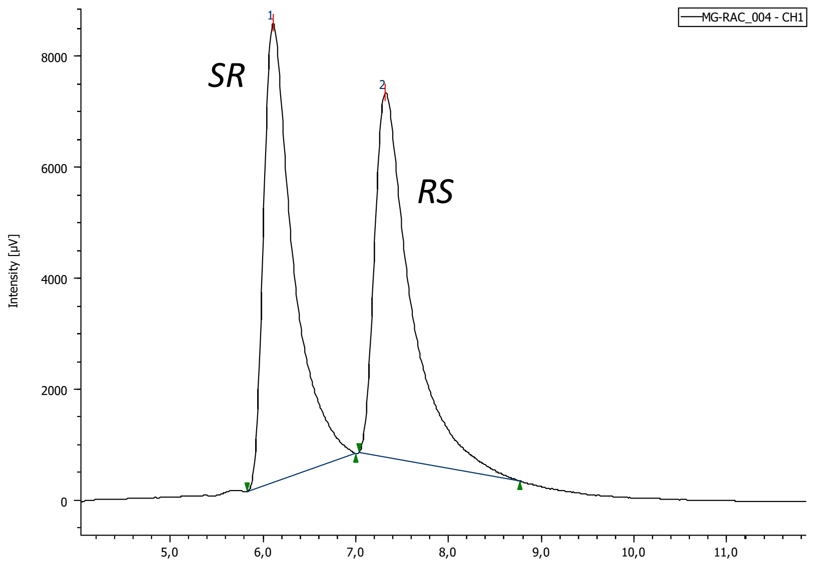

	Time	Area	Area%
1	6,108	184997	**51,011**
2	7,317	177663	**48,989**

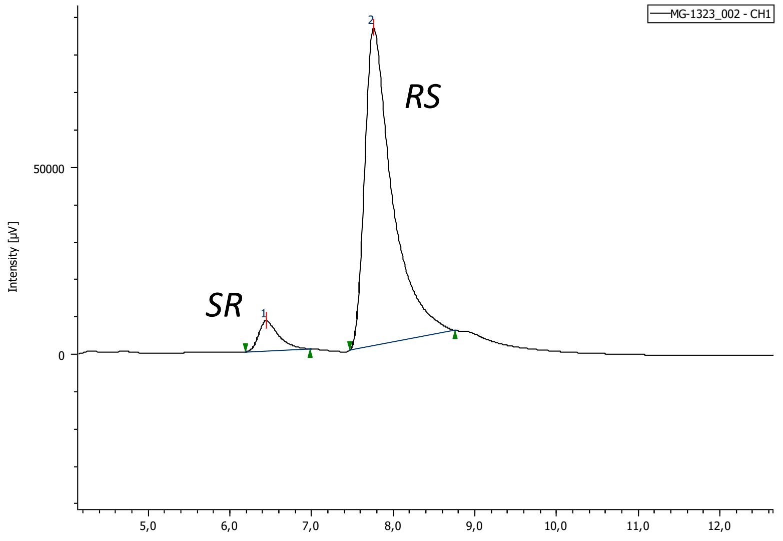

	Time	Area	Area%
1	6,442	138077	**6,436**
2	7,758	2007177	**93,564**

Copies of ^1H NMR, $^{13}C\{^1H\}$ NMR and HPLC profiles of 5

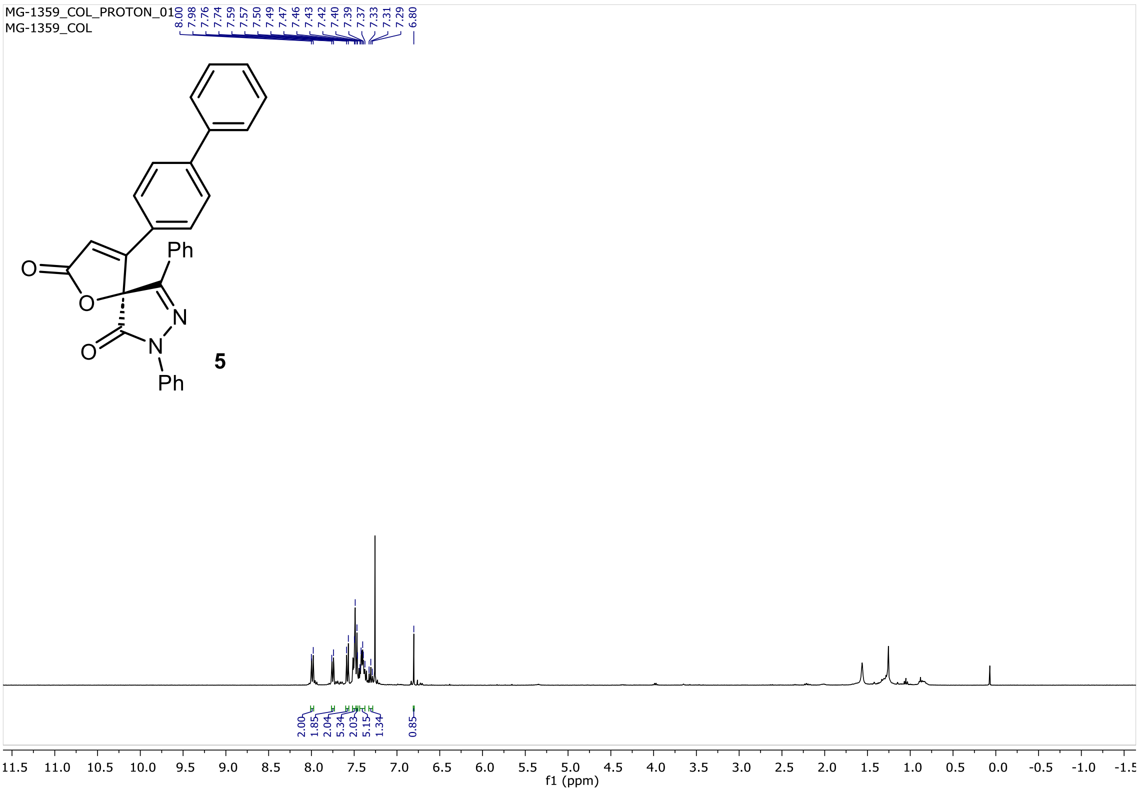

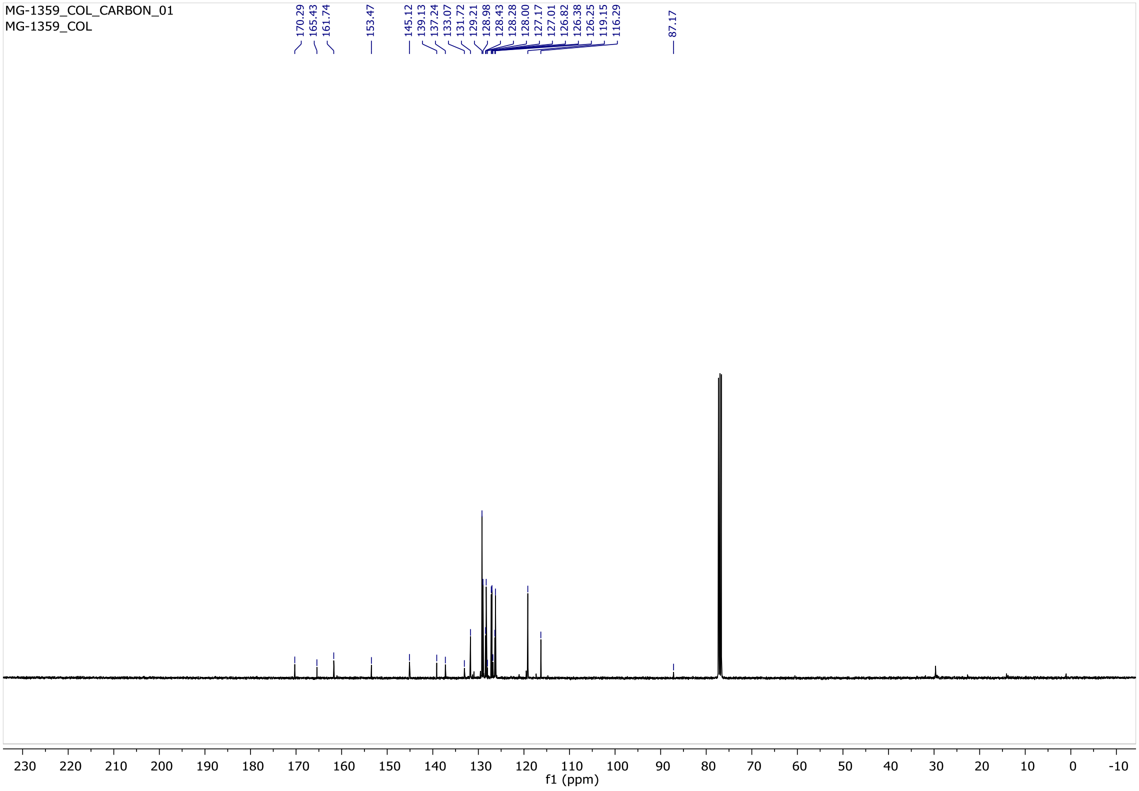

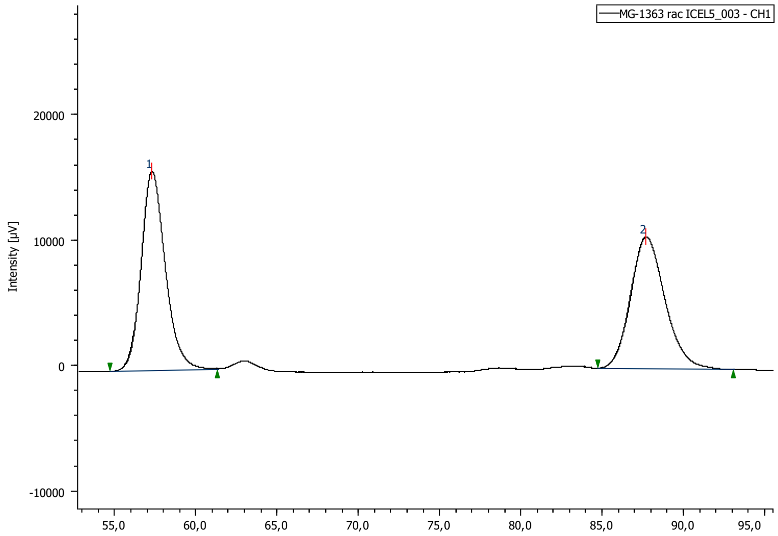

Entry	t_R	Area	Area%
1	57,308	1633106	**51,368**
2	87,658	1546109	**48,632**

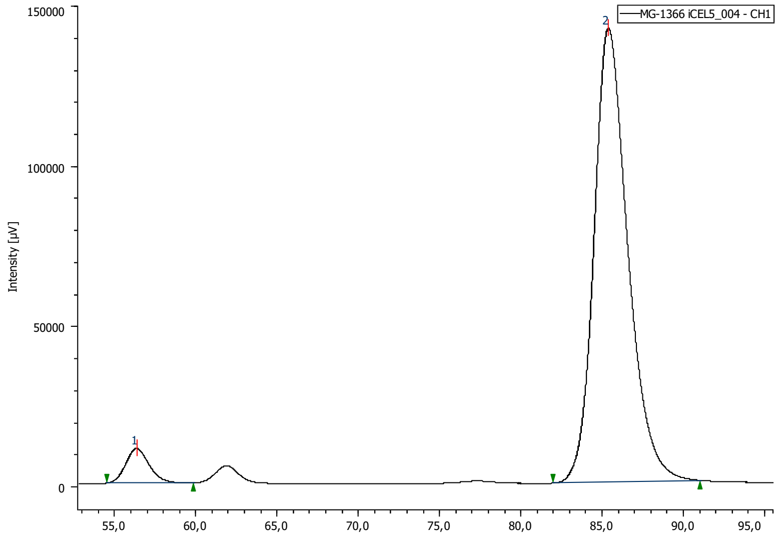

Entry	t_R	Area	Area%
1	56,358	1077196	**5,007**
2	85,350	20438163	**94,993**

Copies of ^{1}H NMR, ^{13}C{^{1}H} NMR and HPLC profiles of 7

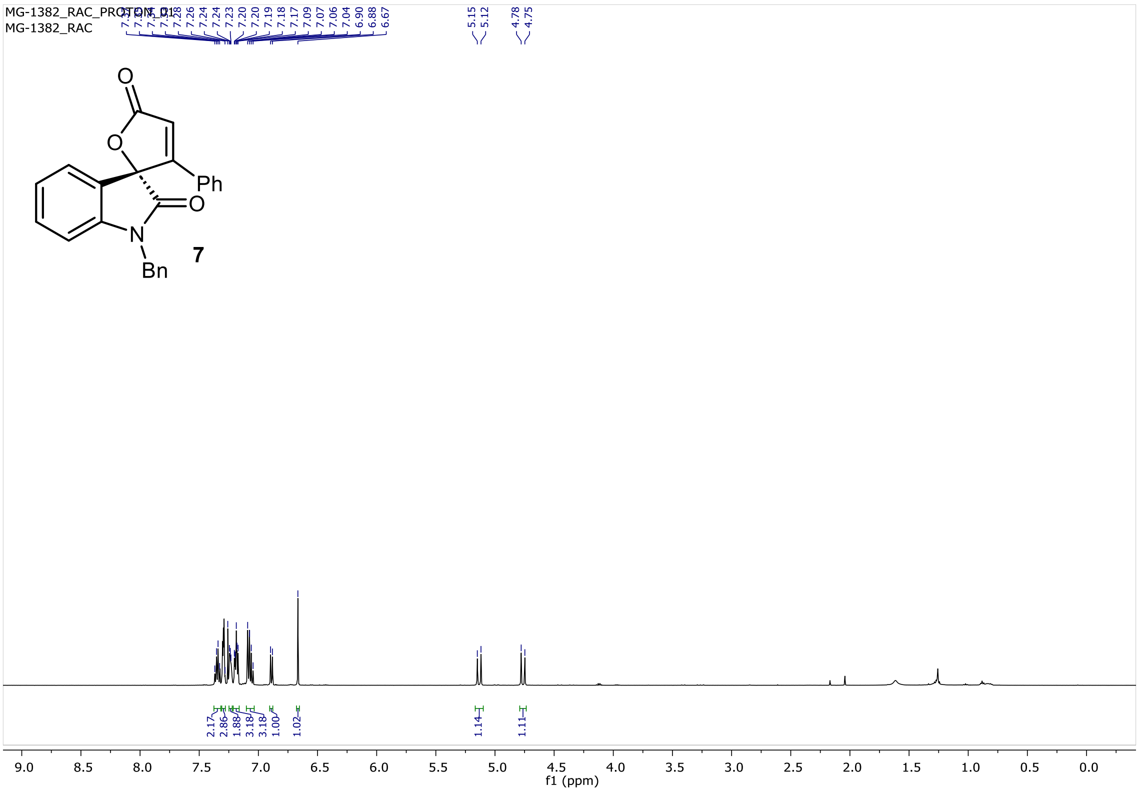

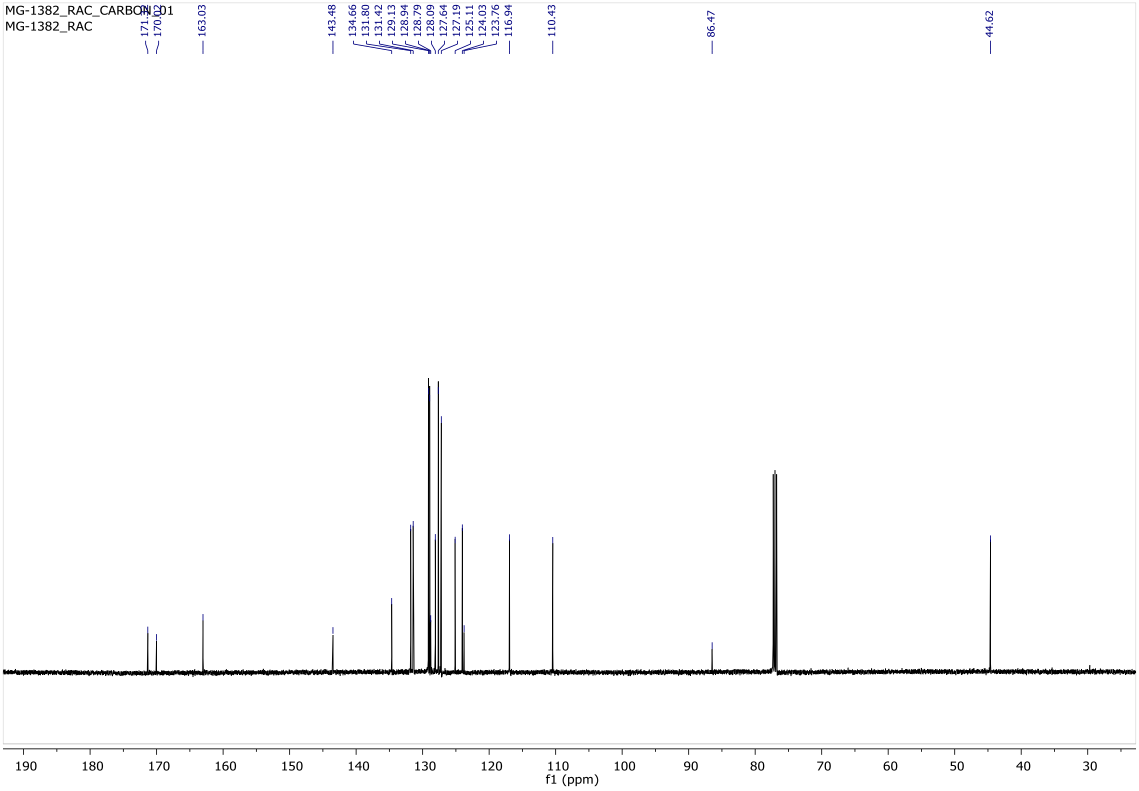

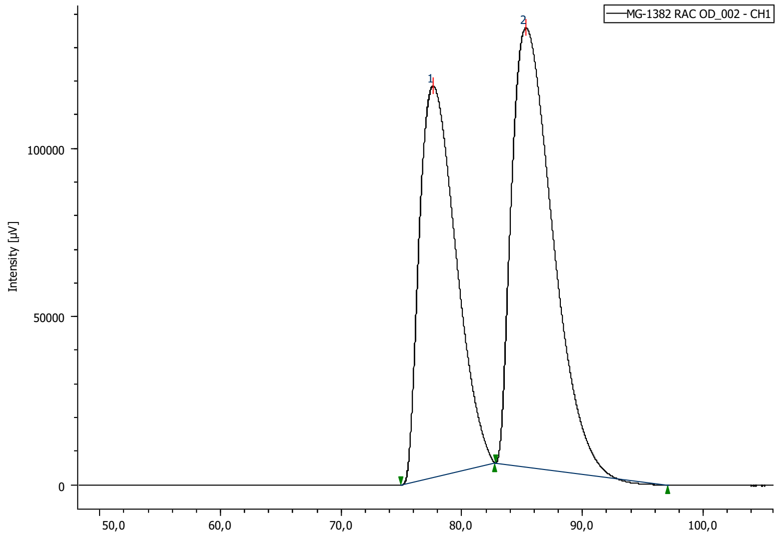

	t_R	Area	Area%
1	77,592	24500054	**44,187**
2	85,250	30946305	**55,813**

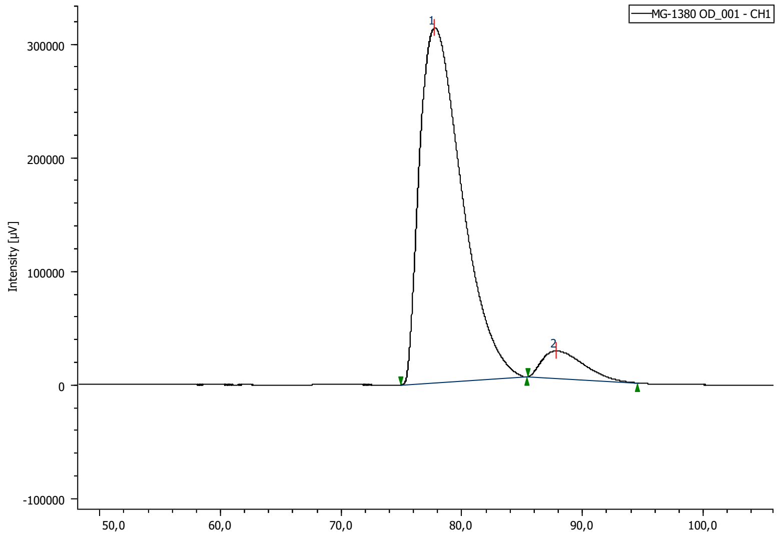

	t_R	Area	Area%
1	77,700	76283329	**92,909**
2	87,767	5821851	**7,091**
